# Cost-effectiveness of pramipexole augmentation for acute phase and maintenance therapy of treatment-resistant depression compared to placebo augmentation: economic evaluation of the PAX-D randomised controlled trial

**DOI:** 10.1016/j.lanepe.2025.101533

**Published:** 2025-11-17

**Authors:** Agata Łaszewska, Timea Helter, Ashley Baldwin, Anthony J. Cleare, Philip J. Cowen, Jonathan Evans, Quentin J.M. Huys, Micheal Kurkar, Alexander C. Lewis, Neil Nixon, Abhinav Rastogi, Stuart Watson, John R. Geddes, Michael Browning, Judit Simon

**Affiliations:** a Center for Public Health, Department of Health Economics, Medical University of Vienna, Vienna, Austria; b Mersey Care NHS Foundation Trust, UK; c Institute of Psychiatry, Psychology and Neuroscience, King's College London, Bristol, UK; d Department of Psychiatry, University of Oxford, Oxford, UK; e Oxford Health NHS Foundation Trust, Oxford, UK; f Bristol Medical School, Bristol Population Health Science Institute, University of Bristol, Bristol, UK; g Applied Computational Psychiatry Lab, Division of Psychiatry and Queen Square Institute of Neurology, University College London, UK; h Pennine Care NHS Foundation Trust, UK; i Mental Health and Clinical Neuroscience, School of Medicine, University of Nottingham, UK; j Midlands Partnership NHS Foundation Trust, UK; k Translational and Clinical Research Institute, Newcastle University, Newcastle upon Tyne, UK; l Cumbria, Northumberland, Tyne and Wear NHS Trust, UK

**Keywords:** Treatment-resistant depression, Pramipexole, Cost-effectiveness analysis, Health economics, Quality of life, Capabilities

## Abstract

**Background:**

Pramipexole augmentation of antidepressant treatment for treatment-resistant depression (TRD) has been shown to improve symptom burden over 12 weeks but with some adverse effects compared to placebo augmentation. We aimed to evaluate the cost-effectiveness of pramipexole augmentation for TRD.

**Methods:**

We conducted an economic evaluation as part of the PAX-D trial over 12 and 48 weeks. Two costing perspectives, National Health Service and Personal Social Services (NHS + PSS) and societal, were adopted. The primary outcome was quality-adjusted life year (QALY) based on the EQ-5D-5L. Secondary outcomes included year of full capability (YFC) based on the ICECAP-A, and capability-weighted life year (CWLY) based on the OxCAP-MH. Incremental cost-effectiveness ratios (ICERs), cost-effectiveness planes and cost-effectiveness acceptability curves were reported alongside sensitivity analyses. The trial was registered with ISCTRN (ISRCTN84666271) and EudraCT (2019-001023-13) and is complete.

**Findings:**

From the NHS + PSS perspective, mean incremental cost of pramipexole was £60 (95% CI: −£55, £176) over 12 weeks and £811 (95% CI: £110, £1513) over 48 weeks. The difference in QALY gained was 0.012 (95% CI: 0.003, 0.021) over 12 weeks and 0.090 (95% CI: 0.036, 0.144) over 48 weeks, equivalent to 4 (95% CI: 1, 8) and 33 (95% CI: 13, 52) days in perfect health. The ICER was £5069/QALY (95% CI: −£3642, £35,608) over 12 weeks and £9007/QALY (95% CI: £2,219, £27,258) over 48 weeks, representing over 90% probability of cost-effectiveness at £20,000/QALY threshold. From the societal perspective, pramipexole was on average cost saving and more effective over 48 weeks. Alternative analyses provided consistent conclusions.

**Interpretation:**

Pramipexole augmentation for TRD has demonstrated both clinical and cost-effectiveness. Further trials, directly comparing pramipexole to other augmentation strategies, will be useful in determining the position of this repurposed medication in the treatment pathway of depression.

**Funding:**

10.13039/501100000272National Institute for Health and Care Research, 10.13039/501100001922Efficacy and Mechanism Evaluation Programme.

**Research in context**

**Evidence before this study**

Treatment-resistant depression (TRD) has substantial economic burden associated with increased direct medical and broader societal costs due to lost productivity and high informal care needs. We searched Medline, PsycInfo, and Scopus between database inception and July 2025, for cost-effectiveness analyses of TRD treatments, with MeSH and free terms related to cost-effectiveness analyses (exp Cost-Benefit Analysis/ or “cost-effectiveness” or “cost-utility” or “economic evaluation∗” or “health economic∗”), and treatment-resistant depression (exp Depressive Disorder, Treatment-Resistant/ or “treatment-resistant depression” or TRD or (resistant adj3 depression)), with no language limits. We found studies published between 2003 and 2025 evaluating a range of interventions for TRD, including antidepressant and pharmacological augmentation strategies (esketamine, ketamine, lithium, quetiapine, and mirtazapine), repetitive transcranial magnetic stimulation, electroconvulsive therapy, deep brain stimulation, and cognitive behavioural therapy as an adjunct to antidepressants. Among studies examining antidepressant treatments and augmentation strategies, cost-effectiveness estimates varied considerably. A US model-based analysis showed that esketamine was unlikely to be cost-effective with an incremental cost-effectiveness ratio (ICER) well over $200,000 per quality-adjusted life year (QALY) when compared to oral antidepressants. Another US study reported that compared to intravenous ketamine, esketamine nasal spray was dominated over three years from the healthcare perspective. In Italy, esketamine augmentation had ICERs ranging from €16,314/QALY to €22,133/QALY from a societal perspective, and exceeded €100,000/QALY from a healthcare perspective, while a US model over 32 weeks found esketamine augmentation to be more cost-effective than quetiapine in both commercial and Medicaid settings. A trial-based economic evaluation of subcutaneous ketamine injections found it to be dominant over control with more than a 90% probability of cost-effectiveness from the Australian healthcare perspective. In the UK context, quetiapine augmentation was more cost-effective than augmentation with lithium over 12 months with a >90% probability of cost-effectiveness at £20,000/QALY threshold from a health and social care perspective, while in a different trial-based analysis, augmentation with mirtazapine was not cost-effective.

No previous studies have evaluated the cost-effectiveness of augmentation with pramipexole for TRD. However, one study assessed its cost-effectiveness relative to placebo for bipolar depression. The study found that pramipexole was on average more effective and saved costs from a health and social care perspective with 86% probability of being cost-effective at £30,000/QALY threshold over 12 weeks and 93% over 48 weeks. From a societal perspective, pramipexole remained on average more effective but was more expensive resulting in a lower probability of cost-effectiveness.

**Added value of this study**

To our knowledge, this is the first cost-effectiveness analysis of pramipexole augmentation in the treatment of TRD. Our findings based on an economic evaluation alongside the PAX-D randomised clinical trial show that pramipexole augmentation compared with placebo is cost-effective both over 12 weeks and 48 weeks. On average, treatment with pramipexole was associated with higher costs and greater health benefits than placebo from a health and social care perspective, with an over 90% probability of being cost-effective at the £20,000/QALY threshold. The study also found that pramipexole augmentation led to longer-term reductions in work absenteeism and informal care needs. From a broader societal perspective, pramipexole augmentation was on average less costly and more effective than placebo augmentation over 48 weeks.

**Implications of all the available evidence**

In patients with TRD, add-on pramipexole titrated to a target dose of 2.5 mg produces substantial benefits in terms of health-related quality of life and capability wellbeing over 12 and 48 weeks of treatment. Both from the perspective of the health and social care system and from the broader society, pramipexole augmentation is cost-effective and represents a good value treatment option despite intolerance issues for some patients.

## Introduction

Depressive disorders are the second highest cause of years lived with disability, showing an increase of 36.5% between 2010 and 2021.^1^ In a recent study, the prevalence of depressive symptoms among adults in the UK ranged from 11.3% for mild symptoms to 3.3% for severe symptoms.^2^ In Europe, the economic burden of mood disorders was estimated at approximately €113 billion in 2010,^3^ and the non-mental health hospital cost for patients with depressive disorders was estimated at €26.5 billion.^4^ Approximately one-third of depression patients suffer from treatment-resistant depression (TRD), which occurs when affected patients do not respond to at least two antidepressants despite the adequacy of the treatment trial and adherence to treatment.^5^ Considering the growing prevalence of depressive disorders, TRD poses an increasing public health challenge. Studies have highlighted especially high direct medical and indirect societal costs among patients with major depressive disorder who experience TRD compared to those who do not experience treatment resistance.^6^ Compared to non-treatment-resistant patients, those with TRD had higher use of healthcare services, including emergency visits and hospitalisations,^7^ higher risks of unemployment, decreased work productivity, and reduced health-related quality of life.^6^^,^^7^ In the UK, the mean societal cost associated with TRD has been estimated at £22,124 with 80% attributed to productivity losses and informal care.^8^

Several treatment options for managing TRD exist including switching or extending the current antidepressant treatment, combining or augmenting antidepressants, using ketamine, esketamine, and neurostimulation.^9^ There is limited evidence on the cost-effectiveness of TRD treatments.^10^ A recent study found that ketamine treatment was cost-effective from the Australian health sector's perspective.^11^ Studies on augmentation of selective serotonin reuptake inhibitor with lithium or atypical antipsychotic drugs, combining/switching antidepressants, cognitive-behavioural therapy, psychotherapy, transcranial magnetic stimulation, and esketamine nasal spray found mixed evidence of their cost-effectiveness (Appendix, ref.1-7). Cleare and colleagues (2025) conducted a head-to-head comparison of the two most commonly used first-line augmentation options for TRD and found that quetiapine augmentation therapy compared to lithium was cost-effective over 12 months.^12^ A recent study by Browning and colleagues (2025) suggested that pramipexole may offer an important advance in TRD therapy by enhancing dopaminergic activity, improving symptoms in patients, though with some adverse effects, in particular, nausea, headache, and sleep disturbance.^13^ The study found a significant reduction in self-reported depressive symptoms (Quick Inventory of Depressive Symptomatology self-report version 16, QIDS-SR16^14^) with an effect size of 3.9 (95% CI: −5.4 to −2.4) from baseline to week 12, also suggesting the potential cost-effectiveness of pramipexole augmentation.^13^ However, no study assessing the cost-effectiveness of pramipexole in TRD has been conducted thus far. This health economic evaluation aimed to assess whether treatment of patients with TRD with pramipexole (treatment) compared to placebo (control) added to their ongoing antidepressant medication is cost-effective over 12 and 48 weeks as part of the PAX-D trial.^14^

## Methods

### Study design

This cost-effectiveness analysis was conducted alongside the multi-site, randomised, double-blind PAX-D trial in which adults with TRD were randomly assigned to 48-week treatment with pramipexole or placebo added to their ongoing antidepressant medication. The trial protocol has previously been published.^15^ We compared pramipexole and placebo augmentation over 12 and 48 weeks, using quality-adjusted life years (QALYs) as the primary health economic outcome measure and years of full capability (YFC) and capability-weighted life years (CWLYs) as secondary measures of broader capability wellbeing. The economic evaluation was conducted in accordance with the National Institute for Health and Care Excellence's (NICE) health technology evaluations manual^16^ adopting the main costing perspective of the National Health Service and Personal Social Services (NHS + PSS) and an alternative broader societal perspective. Relevant health economic outcome and resource use data were collected electronically through the TrueColours clinical platform. The reporting of the findings followed the CHEERS guidelines (Appendix Table S1). The health economic analysis plan was published as part of the PAX-D trial protocol.^15^ Detailed clinical results have recently been published.^13^ The trial was registered with ISCTRN (ISRCTN84666271) and EudraCT (2019-001023-13) and is complete.

### Participants and procedures

Participants were recruited from nine NHS sites and randomised either to pramipexole or placebo. Participants were 18 years or older, met diagnostic criteria for DSM-5 Major Depression, scored >10 on QIDS-SR16,^14^ were taking antidepressant medication and had shown a lack of therapeutic response to an adequate course of at least two antidepressant treatments, defined as at least 4 weeks of treatment (unless limited by tolerability) at the minimum recommended dose.^13^ Recruitment took place between January 2021, and May 2024, with the maximum duration of randomised treatment of 48 weeks. Participants recruited before September 2023 were followed for 48 weeks; those enrolled after this date had progressively shorter follow-up, due to the study's planned end in August 2024. All participants had sufficient follow-up to reach the 12-week primary endpoint. Eligible participants were randomised (1:1) to pramipexole or identical placebo capsules. Pramipexole was administered as immediate-release single dose at night, starting at 0.25 mg, and titrated every 3 days to 2.5 mg over 4 weeks, with dose reductions allowed if side-effects developed.^13^ Placebo followed the same procedure. During the trial, patients received care as usual including antidepressant medication and other treatments for depression. Changes to antidepressant medication were discouraged before week 12 unless considered clinically required by the treating team. People with lived experience were involved in the design and oversight of the PAX-D study.^17^

### Outcomes

In the base-case analysis, QALY gained was selected as the primary outcome measure in accordance with NICE guidelines.^16^ QALYs were calculated using the EQ-5D-5L questionnaire, a preferred measure of health-related quality of life recommended by NICE.^16^ The EQ-5D-5L consists of five dimensions: mobility, self-care, usual activities, pain/discomfort, anxiety/depression. Responses to the EQ-5D-5L questionnaire were valued using the UK-specific crosswalk algorithm,^18^ which maps the EQ-5D-5L to the EQ-5D-3L (range: −0.594 to 1) as currently required by NICE.^16^ Results for the EQ-5D Visual Analogue Scale (EQ VAS) were also reported. To capture broader wellbeing outcomes reflecting challenges experienced by people with TRD in areas such as family life, social life, personal autonomy, freedom, engagement in work and other activities, secondary analyses were conducted using YFC and CWLYs. YFC represents the number of years an individual can expect to live in a state of full capability and was calculated using the ICEpop CAPability measure for Adults (ICECAP-A),^19^ a questionnaire covering five capability dimensions: stability, attachment, autonomy, achievement, and enjoyment with four response levels (1 = no capability; 4 = full capability). ICECAP-A responses were scored using the UK tariff^20^ (range: 0–1). CWLYs were calculated using the Oxford Capability Questionnaire–Mental Health (OxCAP-MH), a 16-item measure developed for mental health research, scored on a five-point scale with higher scores representing better capability wellbeing.^21^ Its standardised score (range: 0–100) was transformed into a 0–1 scale enabling CWLY calculation. All questionnaires have been validated in the mental health context (Appendix, ref. 8–10). Participants completed the questionnaires online using TrueColours at baseline, weeks 12, 24, 36 and 48. QALYs, YFC, and CWLYs were calculated using the area under the curve (AUC) method assuming linear interpolation, by multiplying the amount of time a patient spent in a particular health profile by the utility/capability weighted score associated with that health profile. For all health economic analyses, QALYs, YFC, and CWLYs calculated using the AUC method were adjusted to the EQ-5D-5L, ICECAP-A and OxCAP-MH baseline scores, and expressed their value as a change from baseline.^22^

### Costs

Information on health and social care resource use was collected using the Health Economic Questionnaire (HEQ), developed for mental health economic evaluations.^23^^,^^24^ The HEQ collected patient-reported information about health and social care resource use, medication, informal care, work absenteeism as well as sociodemographics at baseline, weeks 12, 24, 36 and 48 covering the past 12 weeks. Participants completed the HEQ online using TrueColours. Healthcare resource use data included inpatient, outpatient and community healthcare services, and mental-health or non-mental health medications. Hospitalisation and medication information from the HEQ was verified and supplemented using PAX-D medical records. Cost categories for the broader societal perspective included the cost of informal care provided by family or friends and the cost of work absenteeism for participants who were employed or self-employed. Quantities of resource use were multiplied by national UK unit costs in pounds (£) for year 2022/23 (Appendix Table S2) using standard sources. Healthcare resource use costs incurred in the private sector were valued using market prices. Lost productivity costs were estimated using the human capital approach for participants who were employed or self-employed. Where necessary, unit costs were inflated using the NHS Cost Inflation Index report (Appendix Table S2). Direct treatment costs included the cost of the pramipexole medication based on the actual dose taken plus prescription fees aligned to medication dispensing. Scheduled psychiatric visits (at weeks 2, 6, 12, and at study end), as well as unscheduled contacts with the trial clinician related to antidepressant medication or other psychiatric treatments were costed in both study arms. Extra contacts necessary due to pramipexole titration schedule deviations were costed for the treatment arm only.

### Statistical analysis

This within-trial economic evaluation compared patient-level costs, outcomes and incremental cost-effectiveness between the pramipexole and the placebo arms for an intention-to-treat sample over 12 and 48 weeks. All participants with available baseline and 12-week health economic data were included. Missing cost and outcome data at 24, 36, and 48 weeks were imputed using multiple imputation by chained equations (25 imputations matching the overall level of data missingness as reported in Appendix Table S3), with predictive mean matching, adjusted for age, sex (self-reported: male or female), baseline values and site of data collection for each trial arm separately using five donors, under the missing-at-random assumption.^25^^,^^26^ Estimates were pooled using Rubin's rule.^26^ The missing-at-random assumption was checked by comparing baseline health economic outcomes between participants with complete and incomplete data (Appendix Table S4). The imputation was checked for convergence by plotting means of imputed variables against the iteration number.^27^ Changes in health economic outcomes over time were analysed using a linear mixed-effects model, with change from baseline as dependent variable, adjusting for baseline scores, age, sex, follow-up time point, study arm, and a fixed interaction effect between follow-up time and treatment arm. Study site and individual participant ID were included as random effects. Model assumptions (linearity, homoscedasticity, and normality of residuals) were assessed by examining residual plots, Q–Q plots, and histograms. Incremental cost-effectiveness ratio (ICER) was calculated by dividing the mean difference in total costs (incremental costs) by the mean difference in QALYs/YFC/CWLYs gained (incremental effect) between arms. Non-parametric bootstrapping was used to estimate uncertainty, illustrated with cost-effectiveness planes and cost-effectiveness acceptability curves (CEACs). For the primary analysis, net monetary benefit (NMB=λ∗ΔE−ΔC), and net health benefit ($NHB=\Delta E-\left(\Delta C,\lambda \right)$) were calculated from bootstrapped samples at threshold values (λ) of £20,000 and £30,000, where ΔE and ΔC represent differences in outcomes and costs. A positive NMB indicates that at the given threshold, the monetary value of pramipexole's added health benefits is larger than the additional costs needed to achieve these, while a positive NHB suggests that the additional health benefits of pramipexole exceed the health opportunity costs of any displaced care. The intervention was considered cost-effective if the ICER was below £20,000–£30,000 per additional QALY gained.^16^ Secondary analyses for YFC and CWLYs were interpreted using the same thresholds due to the absence of established thresholds for these outcomes. Neither costs nor outcomes required discounting. A two-tailed p-value of <0.05 was used as the threshold for statistical significance. All analyses were undertaken using Stata 18 and Microsoft Excel.

### Sensitivity analyses

A sensitivity analysis was conducted including only participants who remained on the allocated treatment (per protocol, PP), excluding those participants who stopped study treatment at any point. No additional adherence thresholds were predefined. Further sensitivity analyses were conducted for participants who had no missing data on health economic outcomes or resource use over 48 weeks (complete cases) and based on the alternative costing for dispensed medication doses (alternative cost). The potential impact of linear interpolation in outcomes between time points was tested in a separate sensitivity analysis over 48 weeks by assuming that changes happened at the beginning of each given time period (alternative outcome). These sensitivity analyses were performed for all health economic outcome measures (QALYs, YFC, CWLYs) and for both costing perspectives (NHS + PSS, societal). Finally, a sensitivity analysis over 12 and 48 weeks for QALYs gained was performed using the most recent unit cost of pramipexole (current pramipexole cost) for both NHS + PSS and societal perspectives (Appendix Table S2).

### Ethics approval

The trial was approved by the NHS Health Research Authority South West-Central Bristol Research Ethics Committee (IRAS ID: 253,702, REC reference 19/SW/0216). Patients gave written informed consent before entering the study.

### Role of the funding source

The funder had no role in study design, data collection, data analysis, data interpretation, or writing of the report.

## Results

### Study population

From the 150 randomised participants (pramipexole n = 75, placebo n = 75), 124 had complete health economic outcomes and resource use data both at baseline and 12 weeks, and were included in the health economic analyses (pramipexole n = 61, placebo n = 63). Baseline participant characteristics are shown in Table 1 and in Appendix Table S5 for the sensitivity analysis samples. Health economics data missingness over time is reported in Appendix Table S3. A description of the health economic sample, the full randomised sample and the complete cases sample is provided in Appendix Table S4.

**Table 1:** Health economic sample characteristics at baseline.

Participant characteristics at baseline	Pramipexole (n = 61)		Placebo (n = 63)	
	n	% or mean (SD)	n	% or mean (SD)
**Sex**				
Female	33	54%	37	59%
Male	28	46%	26	41%
**Age**	61	43.7 (15.0)	63	45.6 (12.6)
**Depression severity (QIDS-SR16)**				
Mild (<11)	0	0%	0	0%
Moderate (11–15)	26	43%	34	54%
Severe (16–20)	30	49%	25	40%
Very severe (>20)	5	8%	4	6%
**Accommodation**				
Owner occupied/privately rented accommodation	52	85%	54	86%
Housing association/local authority accommodation	9	15%	6	9%
Residential facilities	0	0%	1	2%
Other	0	0%	2	3%
**Living situation**				
Living alone	18	29%	13	21%
Living with others	43	71%	50	79%
**Employment**				
Employed, self-employed or voluntary employed	39	64%	35	55%
Unemployed	8	13%	12	19%
Housewife/-husband	2	3%	2	3%
Student	2	3%	3	5%
Retired	6	10%	8	13%
Other	4	7%	3	5%
**HE outcomes**				
EQ-5D-5L utility index	61	0.50 (0.23)	63	0.49 (0.23)
EQ VAS	61	52.08 (19.19)	63	56.84 (17.58)
OxCAP-MH score	61	56.89 (12.54)	63	56.94 (10.90)
ICECAP-A index	61	0.46 (0.16)	63	0.47 (0.15)

Note: HE, health economics; QIDS-SR16, Quick Inventory of Depressive Symptomatology self-report version 16; EQ-5D-5L, European Quality of Life 5 Dimensions 5 Level; EQ VAS, European Quality of Life Visual Analogue Scale; ICECAP-A, ICEpop CAPability measure for Adults; OxCAP-MH, Oxford Capability Questionnaire—Mental Health.

### Outcome results

Health economic outcome measure results (observed and imputed) are shown for all time points in Appendix Table S6. Differences in changes from baseline between the arms indicated significant additional improvements by pramipexole for all health economic outcome measures for week 12 and 48 (Table 2, Appendix Figs. S1–S3).

**Table 2:** Outcome results (change from baseline in EQ-5D-5L, EQ VAS, ICECAP-A, and OxCAP-MH scores; n = 124).

	Pramipexole (n = 61)^a^	Placebo (n = 63)^a^	Treatment effect (95% CI)^b^	p-value
**Outcomes at week 12**				
Change from baseline to week 12 in EQ-5D-5L utility index score (mean [SD])	0.137 (0.198)	0.041 (0.192)	0.097 (0.043–0.152)	<0.0001
Change from baseline to week 12 in EQ VAS score (mean [SD])	8.93 (19.17)	−0.698 (17.96)	7.42 (1.38–13.46)	0.016
Change from baseline to week 12 in ICECAP-A score (mean [SD])	0.131 (0.199)	0.048 (0.152)	0.082 (0.021–0.144)	0.009
Change from baseline to week 12 in OxCAP-MH score (mean [SD])	9.09 (10.25)	3.82 (9.09)	5.28 (3.14–7.42)	<0.0001
**Outcomes at week 48**				
Change from baseline to week 48 in EQ-5D-5L utility index score (mean [SD])	0.103 (0.233)	0.039 (0.201)	0.067 (0.004–0.129)	0.036
Change from baseline to week 48 in EQ VAS score (mean [SD])	10.18 (17.62)	1.71 (15.64)	6.26 (4.51–8.00)	<0.0001
Change from baseline to week 48 in ICECAP-A score (mean [SD])	0.176 (0.172)	0.093 (0.169)	0.083 (0.044–0.122)	<0.0001
Change from baseline to week 48 in OxCAP-MH score (mean [SD])	7.93 (9.91)	4.95 (9.16)	2.99 (1.30–4.68)	0.001

Note: EQ-5D-5L, European Quality of Life 5 Dimensions 5 Level; EQ VAS, European Quality of Life Visual Analogue Scale; ICECAP-A, ICEpop CAPability measure for Adults; OxCAP-MH, Oxford Capability Questionnaire—Mental Health.

a Observed values.

b Adjusted results of the linear mixed effects regression model adjusted for randomised group, timepoint, age, sex, baseline score, an interaction between randomised group and timepoint, and baseline value of outcome, as fixed effects; site and participant as random effects. A positive mean difference indicates improvement in favour of pramipexole.

### Cost results

Healthcare resource utilisation is shown in Appendix Table S7. Baseline costs did not differ statistically between the study arms (Appendix Table S8) and there were no significant cost differences between the arms over 12 weeks either, except for the cost of pramipexole (Table 3). Over 48 weeks, costs from the NHS + PSS perspective were significantly higher for pramipexole (+£811, 95% CI: £110–£1513). The largest cost differences were observed for non-mental health inpatient services (£795 vs. £271), medication costs (£401 vs. £306), and primary care costs (£96 vs. £15), with the differences in non-mental health inpatient and primary care costs being significant. There were no significant broader societal cost differences between the arms, but absenteeism and informal care costs showed cost saving tendencies by pramipexole (£615 vs. £977 and £1398 vs. £2102, respectively).

**Table 3:** Cost results (in £, for year 2022/2023; n = 124).

	0–12 weeks					0–48 weeks				
	Pramipexole (n = 61)		Placebo (n = 63)		Δ costs [95% CI]	Pramipexole (n = 61)		Placebo (n = 63)		Δ costs [95% CI]
	Mean	SD	Mean	SD		Mean	SD	Mean	SD	
**Medication**	111.58	100.21	98.3	178.39	13.27 [−38.39 to 64.94]	401.37	313.23	306.3	318.37	95.07 [−17.23 to 207.39]
Pramipexole^a^	29.66	5.66	0	0	29.66∗∗∗ [28.25–31.07]	80.03	40.04	0	0	80.03∗∗∗ [70.04–90.01]
Antidepressants	34.67	48.8	27.83	36.88	6.84 [−8.50 to 22.18]	127	109.81	153.52	124.22	−26.51 [−68.25 to 15.21]
Other MH medication	3.23	14	4.03	21.49	−0.79 [−7.26 to 5.67]	7.11	20.03	12.29	29.61	−5.18 [−14.19 to 3.82]
NMH medication	44.01	86.83	66.45	176.5	−22.43 [−72.13 to 27.27]	187.23	271.68	140.48	276.06	46.74 [−50.65 to 144.15]
**Inpatient**	45.60	270	8.71	69.10	36.88 [−32.68 to 106.46]	794.62	1604.21	271.46	683.82	523.15∗ [87.16–959.14]
MH Inpatient	0	0	0	0	–	0	0	0	0	–
NMH Inpatient	45.60	270	8.71	69.1	36.88 [−32.68 to 106.46]	794.62	1604.21	271.46	683.82	523.15∗ [87.16–959.14]
**Outpatient services**	300.73	74.15	286.61	55.82	14.12 [−9.15 to 37.41]	621.52	336.29	562.39	290.03	59.12 [−52.39 to 170.65]
MH Outpatient care^b^	288.60	36.76	280.74	32.39	7.86 [−4.43 to 20.17]	495.82	111.22	505.06	202.53	−9.23 [−67.59 to 49.11]
NMH Outpatient care	12.13	62.81	5.87	46.61	6.25 [−13.36 to 25.87]	125.69	275.68	57.33	201.58	68.36 [−17.28 to 154.02]
**Community care services**	2.87	16.14	4.86	27.03	−1.98 [−9.93 to 5.95]	76.59	416.39	23.59	74.11	52.99 [−52.52 to 158.52]
MH Community care	0	0	1.08	8.57	−1.07 [−3.25 to 1.09]	22.15	160.21	15.41	56.91	6.73 [−35.73 to 49.21]
NMH Community care	2.87	16.14	3.78	21.99	−0.91 [−7.78 to 5.96]	54.44	257.56	8.18	27.55	46.25 [−18.34 to 110.86]
**Primary care**	1.46	8	3.41	16.07	−1.95 [−6.48 to 2.58]	95.87	194.14	15.35	43.08	80.51∗∗ [30.88–130.14]
**NHS + PSS Perspective**	462.24	402.32	401.89	229.51	60.34 [−55.62 to 176.32]	1989.97	2600.66	1179.09	1050.24	810.88∗ [109.82–1512.93]
Absenteeism	130.54	406.46	119.59	336.21	10.94 [−121.47 to 143.37]	615.28	1002.45	977.25	1127.44	−361.97 [−741.67 to 17.72]
Informal care	632.07	1652.53	653.14	2192.45	−21.07 [−712.96 to 670.81]	1398.41	1577.37	2102.19	2592.94	−703.78 [−1469.79 to 62.21]
**Societal perspective**	1224.84	1903.37	1174.62	2307.63	50.22 [−703.09 to 803.53]	4003.65	3898.70	4258.53	3862.23	−254.88 [−1621.18 to 1111.41]

MH, Mental health; NMH, Non-mental health; NHS + PSS, National Health Service and Personal Social Services; GP, general practice; CI, confidence interval.

∗p-value <0.05, ∗∗p-value <0.01, ∗∗∗p-value <0.001.

a Cost of pramipexole including prescription fee.

b Data on outpatient psychiatrist visits from the HEQ combined with the pre-schedule trial visits at week 2, 6 and 12, and non-scheduled visit when study participant changed dose or medication of pramipexole or antidepressants.

### Cost-effectiveness results

**Fig. 1:**
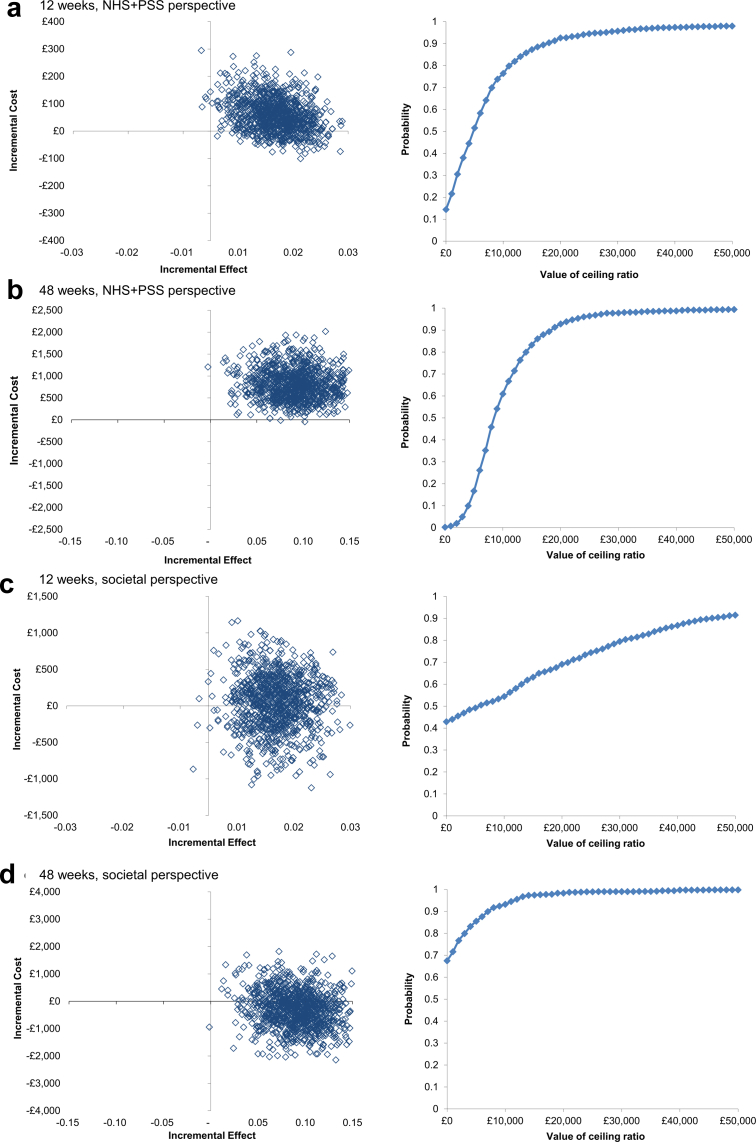
Primary cost-effectiveness analysis results: National Health Service and Personal Social Services (NHS + PSS) perspective and societal perspective. Note: Left: Cost-effectiveness plane with bootstrapped incremental cost-effectiveness ratios (ICERs) for pramipexole against placebo treatment presenting costs per quality-adjusted life year (QALY) gained: a) over 12 weeks from the NHS + PSS perspective; b) over 48 weeks from the NHS + PSS perspective; c) over 12 weeks from the societal perspective; d) over 48 weeks from the societal perspective. Right: Cost-Effectiveness Acceptability Curves showing the probability of pramipexole being cost-effective in comparison to placebo treatment at different willingness-to-pay thresholds for QALY gained.

From the NHS + PSS perspective, pramipexole was associated with significantly higher QALYs gained (+0.012 QALY, 95% CI: 0.003–0.021), representing four (95% CI: 1–8) additional days of perfect health, and an ICER of £5069/QALY (95% CI: −£3642 to £35,608) over 12 weeks (Table 4). Over 48 weeks, the incremental QALYs gained was +0.090 (95% CI: 0.036–0.144), equivalent to 33 (95% CI: 13–52) days in perfect health, resulting in an ICER of £9007/QALY (95% CI: £2219–£27,258). Pramipexole being more costly and more effective at 12 and 48 weeks was also reflected in the cost-effectiveness plane (Fig. 1) as the majority of the bootstrapped cost-effectiveness ratios were located in the northeast quadrant (Table 4). From the societal perspective, the ICER was £4218/QALY (95% CI: −£82,550 to £99,345) over 12 weeks, while pramipexole showed on average lower costs and higher effects with the majority of bootstrapped ICERs located in the southeast quadrant over 48 weeks (Table 4, Fig. 1). Overall, the probability of pramipexole being cost-effective from the NHS + PSS perspective was over 90% at a WTP threshold of £20,000/QALY gained at both timepoints. Secondary cost-effectiveness analyses showed similar results when the same theoretical thresholds were applied (Table 4, Appendix Figs. S4–S7). All sensitivity analyses arrived at similar conclusions as presented in the main cost-effectiveness analysis, although in the 48-week complete case analysis (n = 56) the incremental effectiveness of pramipexole was notably lower (0.038, 95% CI: −0.050 to 0.126) with larger uncertainty in cost-effectiveness (ICER £20,840/QALY, 95% CI: −£166,588 to £207,354) than in the main analysis (Appendix Table S10, Appendix Figs. S8–S19). Using the most recent pramipexole unit cost estimates for year 2024/25 increased the mean cost of pramipexole augmentation resulting in somewhat higher ICERs. Nevertheless, the mean ICERs remained below £20,000/QALY and the probability of cost-effectiveness between 82% and 96% (Appendix Table S10, Appendix Figs. S20 and S21).

**Table 4:** Main cost-effectiveness results (n = 124).

	ΔC [95% CI]	ΔE [95% CI]	ICER^a^ [95% CI]	Interpretation	Distribution on cost-effectiveness plane (in %)				Probability of CE at £20,000/£30,000 WTP
					NE	SE	SW	NW	
Primary outcome measure									
QALY (EQ-5D-5L): NHS + PSS perspective over 12 weeks	£60 [−£55 to £176]	0.012 [0.003–0.021]	£5069/QALY [−£3642 to £35,608]	Pramipexole on average is more expensive and more effective than placebo	85%	14.5%	0%	0.5%	93%/96%
QALY (EQ-5D-5L): NHS + PSS perspective over 48 weeks	£811 [£109–£1513]	0.090 [0.036–0.144]	£9007/QALY [£2219–£27,258]	Pramipexole on average is more expensive and more effective than placebo	99.7%	0.2%	0%	0.1%	93%/98%
QALY (EQ-5D-5L): Societal perspective over 12 weeks	£50 [−£693 to £794]	0.012 [0.003–0.021]	£4218/QALY [−£82,550 to £99,345]	Pramipexole on average is more expensive and more effective than placebo	57%	42.8%	0%	0.2%	69%/79%
QALY (EQ-5D-5L): Societal perspective over 48 weeks	−£255 [−£1621 to £1111]	0.090 [0.036–0.144]	−£2831/QALY [−£20,189 to £15,815]	Pramipexole on average saves costs and is more effective than placebo	33%	67%	0%	0%	98%/99%
Secondary outcome measures									
YFC (ICECAP-A): NHS + PSS perspective over 12 weeks	£60 [−£55 to £176]	0.010 [0.002–0.018]	£5852/YFC [−£5155 to £34,401]	Pramipexole on average is more expensive and more effective than placebo	85.5%	14.2%	0%	0.3%	92%/96%
YFC (ICECAP-A): NHS + PSS perspective over 48 weeks	£811 [£109–£1513]	0.064 [0.019–0.109]	£12,604/YFC [£3372–£48,586]	Pramipexole on average is more expensive and more effective than placebo	99.7%	0.1%	0%	0.2%	79%/92%
CWLY (OxCAP-MH): NHS + PSS perspective over 12 weeks	£60 [−£55 to £176]	0.007 [0.002–0.011]	£9155/CWLY [−£7639 to £43,768]	Pramipexole on average is more expensive and more effective than placebo	86.8%	13.2%	0%	0%	84%/93%
CWLY (OxCAP-MH): NHS + PSS perspective over 48 weeks	£811 [£109–£1513]	0.038 [0.012–0.064]	£21,320/CWLY [£5222–£62,592]	Pramipexole on average is more expensive and more effective than placebo	99.7%	0.1%	0%	0.2%	44%/75%
YFC (ICECAP-A): societal perspective over 12 weeks	£50 [−£693 to £794]	0.010 [0.002–0.018]	£4870/YFC [−£105,686 to £114,390]	Pramipexole on average is more expensive and more effective than placebo	54.2%	45.3%	0.1%	0.4%	65%/75%
YFC (ICECAP-A): societal perspective over 48 weeks	−£255 [−£1621 to £1111]	0.064 [0.019–0.109]	−£3962/YFC [−£31,283 to £23,999]	Pramipexole on average saves costs and is more effective than placebo	35.7%	64%	0.2%	0.1%	97%/98%
CWLY (OxCAP-MH): societal perspective over 12 weeks	£50 [−£693 to £794]	0.007 [0.002–0.011]	£7618/CWLY [−£148,086 to £135,157]	Pramipexole on average is more expensive and more effective than placebo	53.8%	46.2%	0%	0%	60%/67%
CWLY (OxCAP-MH): societal perspective over 48 weeks	−£255 [−£1621 to £1111]	0.038 [0.012–0.064]	−£6702/CWLY [−£66,865 to £34,634]	Pramipexole on average saves costs and is more effective than placebo	36.8%	63.2%	0%	0%	91%/96%

The discrepancy between the table values and the ICERs is due to this rounding. Confidence intervals for the ICERs were calculated using bootstrap methods; QALY, quality-adjusted life year; YFC, year of full capability; CWLY, capability-weighted life year; ICER, incremental cost-effectiveness ratio; CI, confidence interval; NHS + PSS, National Health Service and Personal Social Services; NE: Δ costs >0, Δ effect >0; SE: Δ costs <0, Δ effect >0; SW: Δ costs <0, Δ effect <0; NW: Δ costs >0, Δ effect <0; EQ-5D-5L, European Quality of Life 5 Dimensions 5 Level; ICECAP-A, ICEpop CAPability measure for Adults; OxCAP-MH, Oxford Capability Questionnaire—Mental Health.

a ICERs presented in the table are calculated from the unrounded cost and outcome differences (including decimal points), while the values in the table are rounded for simplicity.

The NMB and NHB analyses showed positive values both at 12 weeks (NMB: £177, 95% CI −£68 to £401; NHB: 0.009, 95% CI −0.003 to 0.020) and 48 weeks (NMB: £989, 95% CI −£360 to £2210; NHB: 0.050, 95% CI −0.018 to 0.111) from the NHS + PSS costing perspective, confirming an overall population health gain by TRD augmentation therapy with pramipexole compared to placebo at the current UK cost-effectiveness threshold range of £20,000-£30,000/QALY (Appendix Table S11).

## Discussion

This study investigated the cost-effectiveness of pramipexole augmentation compared to placebo augmentation for patients with TRD. Using the NHS + PSS perspective, pramipexole was on average more costly and more effective than placebo with mean ICERs below the £20,000/QALY threshold across most scenarios, and probability of cost-effectiveness of 93% over 12 and 48 weeks based on the primary outcome measure. In the analyses conducted from the societal perspective pramipexole was more cost-effective and on average less costly and more effective over 48 weeks, suggesting pramipexole being a dominant alternative. However, this finding needs to be interpreted with caution due to wide confidence intervals. Sensitivity analyses were consistent with the main analysis, with the largest decrease in the probability of cost-effectiveness to 54% observed in the 48-week complete case analysis from the NHS + PSS perspective.

While no comparable health economic study has been identified, previous trials examining the efficacy of pramipexole in TRD showed promising results in terms of its effectiveness (Appendix, ref. 11–13). A recent study of augmentation strategies in TRD found quetiapine to be more cost-effective compared to lithium from the NHS + PPS perspective, with a 99% probability of cost-effectiveness considering a £20,000/QALY threshold,^12^ which is comparable to the main estimates derived in our study. Other augmentation or stand-alone therapies including mirtazapine^28^ or esketamine^29^ were not cost-effective. Our study showed more favourable ICER estimates compared to the cost-effectiveness of non-pharmacological approaches to treat TRD. A model-based study of deep brain stimulation vs. treatment as usual has shown the ICER being between $31,878 and $254,719/QALY over a 5-year time horizon from the healthcare sector perspective.^30^ In another trial-based economic evaluation of long-term psychoanalytic psychotherapy vs. treatment as usual, the ICER was £33,000/QALY with only 18% probability of being cost-effective at a £20,000/QALY threshold over 3.5 years.^31^ Similarly to our findings, a recent study in bipolar depression found pramipexole to be cost-effective compared to placebo in the UK with variations according to the perspective: pramipexole was on average more effective and less costly from the NHS + PSS perspective and more effective and more costly from the societal perspective.^32^

Further aspects of our findings are worth additional considerations. On average, pramipexole significantly improved all health-related quality of life and broader wellbeing outcomes compared to placebo. Total costs from the NHS + PSS perspective were higher in the pramipexole arm than the placebo arm. Higher costs were related to the cost of pramipexole itself, primary care visits, and non-mental health inpatient admissions. The latter costs were infrequently reported with a single participant accounting for 69% of these costs in the pramipexole arm, and were not related to any (serious) adverse event linked to the treatment. At the same time, the cost of antidepressants was lower in the treatment group than in the control group. While this difference was not statistically significant, it may still reflect a potential benefit of pramipexole also through the reduction in necessary antidepressant treatment. Other health care costs were similar in the two study arms. In the analysis from the societal perspective, the beneficial tendencies of pramipexole outweighed the increased health care costs by reducing informal care needs and improving productivity. Although pramipexole is known to cause side effects,^33^ we found that the overall improvement in quality of life and broader wellbeing and the reduction in work absenteeism and informal care together with its cost-effectiveness supports potential implementation as augmentation treatment for TRD.

In terms of methods, our study followed trial-based economic evaluation guidelines ensuring that best practice methods were used.^16^^,^^34^ The 12-week analysis was conducted on fully complete data with no missing values. Over 48 weeks, an additional complete case sensitivity analysis was conducted to provide some inference on the impact of imputed missing data in the main analysis. Multiple other sensitivity analyses were conducted, including one based on costs driven by NHS dispensing dose regimes, to explore further cost-effectiveness implications of potential real-world implementation. The inclusion of scheduled visits with the trial psychiatrist in the main analysis at weeks 2, 6, 12 and trial end, and unscheduled visits for dose adjustment and medication changes, likely reflected routine monitoring that would occur following pramipexole rollout. These clinical consultations were not strictly research-related and appear to have replaced standard mental health outpatient visits in both arms, making the associated costs broadly representative of real-world healthcare use. Finally, multiple outcome measures including measures of capability wellbeing were used making sure that broader wellbeing benefits that are of a high importance for TRD patients were also considered.

Several limitations should be considered when interpreting the main cost-effectiveness results. Although missing data were assumed to be missing at random, we cannot verify this, and the 48-week results should be interpreted with care. While withdrawal rates were similar in both study arms, it is possible that patients who withdrew had worse overall health-related quality of life compared to those who remained in the trial, which was not feasible to account for in the analysis. Additionally, due to the extension of the recruitment period but not the entire trial period, participants recruited after September 2023 were not able to reach 48-week follow-up. To address this issue, a sensitivity analysis was conducted using complete 48-week data only. Baseline health economic outcomes were found to be comparable between the complete cases sample and the main analysis sample (Appendix Table S4), suggesting it is unlikely that this aspect of the trial would have differed between the two arms. Nevertheless, the smaller sample size at 48 weeks stemming from the lower number of complete cases and varying follow-up periods, reduced the precision of our estimates and increased the uncertainty around incremental costs and effects impacting also the probability of cost-effectiveness. Overall, 20% of the participants in the pramipexole arm discontinued study medication, mostly due to intolerance, compared with 5% of those receiving placebo.^13^ While the main analysis reflects average effectiveness across all study participants on an intention-to-treat basis, the PP sensitivity analysis, which showed more favourable outcomes, reflects cost-effectiveness among those who likely tolerate pramipexole. Finally, generalisability of the study findings beyond the UK is limited given differences in the organisation and cost of mental health care across countries.

In conclusion, pramipexole augmentation is a cost-effective treatment for patients with TRD, demonstrating consistent benefits across multiple outcomes and perspectives. We found that the overall improvement in quality of life and broader wellbeing along with reductions in work absenteeism and informal care and its overall cost-effectiveness all support the potential implementation of pramipexole as augmentation treatment for TRD.

## Contributors

JS, MB, JRG, AJC, PJC, SW conceptualised the study and acquired funding. MB, AB, JE, QJMH, MK, NN, AR supervised the PAX-D clinical study and were involved in data acquisition. JS supervised the health economic study. JS, TH and AŁ were involved in the methodology and data acquisition. AŁ and JS directly accessed and verified the data reported in this manuscript and completed the analyses. JS, AŁ and MB interpreted the results. ACL and TH were involved in project administration and data acquisition. AŁ and JS wrote the manuscript. All authors were involved in the reviewing and editing of the manuscript. All authors confirm that they had full access to all the data in the study and accept responsibility to submit for publication.

## Data sharing statement

De-identified individual clinical trial participant-level data will be made available for sharing in ethically approved individual patient data synthesis and meta-analyses on receipt of an appropriate application and subject to approval by the PAX-D chief investigator (michael.browning@psych.ox.ac.uk).

## Declaration of interests

AJC reports grants from the ADM Protexin, Beckley Psytech; consulting fees from Compass Pathways, Otsuka, Janssen; payment or honoraria for presentations or lectures from Compass Pathways, Otsuka, Viatris, and Medscape; and is President of the International Society for Affective Disorders. PJC reports grants from National Institute for Health and Care Research (NIHR) Efficacy and Mechanism Evaluation (EME) scheme. QJMH has obtained support by the NIHR University College London Hospitals NHS Foundation Trust (UCLH) Biomedical Research Centre, NIHR EME scheme; research grants from Wellcome Trust, NIHR, Carigest SA, Koa Health; consultancy fees and options from Aya Technologies and Alto Neuroscience. ACL reports salary support from the NIHR EME scheme. NN reports grants from the Wellcome Trust, NIHR, and BlueSkeye AI; consulting fees from National Institute for Health and Care Excellence (NICE) Scientific; sponsored attendance at meetings from Compass Pathways. MB reports grants from the NIHR EME scheme, NIHR Oxford Health Biomedical Research Centre, Office for Life Science and NIHR Mental Health Translational Research, NIHR Oxford Health Clinical Research Facility Collaboration, Wellcome Trust, Medical Research Council; consulting fees from Engrail Therapeutics, Jansen Research, Boehringer, CHDR, P1vital, Alto Neuroscience, and Empyrean Therapeutics; and was previously employed by P1vital. JS reports support from NIHR; grants from WWTF (Vienna Science and Technology Fund), EC (the European Commission), NIHR, FWF (Austrian Science Fund; payments or honoraria from EBC (the European Brain Council). All other authors declare no competing interests.
