# Supplementary material for: Cost-effectiveness of pramipexole augmentation for acute phase and maintenance therapy of treatment-resistant depression compared to placebo augmentation: economic evaluation of the PAX-D randomised controlled trial
Source: Lancet Reg Health Eur. 2025 Nov 17;61:101533. doi: 10.1016/j.lanepe.2025.101533 (PMC12666355; doi:10.1016/j.lanepe.2025.101533)
Supplement: Appendix Figures and Tables [file mmc1.docx]

**Supplementary appendix**

**Cost-effectiveness of** **pramipexole augmentation for acute phase and maintenance therapy of Treatment-Resistant Depression compared to placebo augmentation: economic evaluation of the PAX-D randomised controlled trial**

Agata Łaszewska, PhD^1^, Timea Helter, PhD^1^, Prof Ashley Baldwin, MD^2^, Prof Anthony J Cleare, PhD^3^, Prof Phil J Cowen, MD^4,5^, Jonathan Evans, MD^6^, Prof Quentin J M Huys, MD^7^, Micheal Kurkar, MD^8^, Alexander C Lewis, BSc^4^, Neil Nixon, MD^9^, Abhinav Rastogi, FRCPsych^10^, Stuart Watson, MD^11,12^, Prof John R Geddes, MD^4,5^, Prof Michael Browning, DPhil, ^4,5^ Prof Judit Simon, DPhil^1,4^

^1^Department of Health Economics, Center for Public Health, Medical University of Vienna, Vienna, Austria; ^2^Mersey Care NHS Foundation Trust, UK; ^3^Institute of Psychiatry, Psychology and Neuroscience, King's College London, Bristol, UK; ^4^Department of Psychiatry, University of Oxford, Oxford, UK; ^5^Oxford Health NHS Foundation Trust, Oxford, UK; ^6^Bristol Medical School, Bristol Population Health Science Institute, University of Bristol, Bristol, UK; ^7^University College London, UK; ^8^Pennine Care NHS Foundation Trust, UK; ^9^Mental Health and Clinical Neuroscience, School of Medicine, University of Nottingham, UK; ^10^Midlands Partnership NHS Foundation Trust, UK; ^11^Translational and Clinical Research Institute, Newcastle University, Newcastle upon Tyne, UK; ^12^Cumbria, Northumberland, Tyne and Wear NHS Trust, UK

Correspondence: Judit Simon, Department of Health Economics, Center for Public Health, Medical University of Vienna, Kinderspitalgasse 15/1, 1090 Vienna, Austria; [judit.simon@meduniwien.ac.at](mailto:judit.simon@meduniwien.ac.at)

**Table of Contents**

[Supplementary references 4](#_Toc211862891)

[Tables 6](#_Toc211862892)

[Appendix Table 1. CHEERS 2022 Checklist 6](#_Toc211862893)

[Appendix Table 2. Unit costs (in £ for year 2022/2023) 8](#_Toc211862894)

[Appendix Table 3. Data availability by treatment arm (n=124) 10](#_Toc211862895)

[Appendix Table 4. Baseline characteristics of the full randomised sample, health economic sample, and complete cases sample 11](#_Toc211862896)

[Appendix Table 5. Sensitivity analysis sample characteristics at baseline 12](#_Toc211862897)

[Appendix Table 6. Health outcome measure results over time (n=124) 13](#_Toc211862898)

[Appendix Table 7. Resource use results: available cases 15](#_Toc211862899)

[Appendix Table 8. Baseline cost results (in £, for year 2022/2023) 17](#_Toc211862900)

[Appendix Table 9. Sensitivity analyses: Imputed EQ-5D-5L, EQ VAS, ICECAP-A, OxCAP-MH results for per protocol (PP) sample (n=103) and complete cases sample (n=56) 18](#_Toc211862901)

[Appendix Table 10. Sensitivity analyses: Mean incremental costs, QALYs, YFC, CWLYs and ICERs (pramipexole vs. placebo) 20](#_Toc211862902)

[Appendix Table 11. Results of primary net monetary benefit (NMB) and net health benefit (NHB) analyses 22](#_Toc211862903)

[Figures 23](#_Toc211862904)

[Appendix Figure 1 Health economic outcome measure results over time (n=124) 23](#_Toc211862905)

[Appendix Figure 2. Sensitivity analysis: health economic outcomes for PP sample (n=103) 24](#_Toc211862906)

[Appendix Figure 3. Sensitivity analysis: health economic outcomes for complete cases sample (n=56) 25](#_Toc211862907)

[Appendix Figure 4. Secondary cost-effectiveness analysis for YFC (NHS+PSS perspective) 26](#_Toc211862908)

[Appendix Figure 5. Secondary cost-effectiveness analysis for CWLY (NHS+PSS perspective) 27](#_Toc211862909)

[Appendix Figure 6. Secondary cost-effectiveness analysis for YFC (societal perspective) 28](#_Toc211862910)

[Appendix Figure 7. Secondary cost-effectiveness analysis for CWLY (societal perspective) 29](#_Toc211862911)

[Appendix Figure 8. Sensitivity analysis: Cost-effectiveness analysis for QALY (per protocol) 30](#_Toc211862912)

[Appendix Figure 9. Sensitivity analysis: Cost-effectiveness for YFC (per protocol) 31](#_Toc211862913)

[Appendix Figure 10. Sensitivity analysis: Cost-effectiveness for CWLY (per protocol) 32](#_Toc211862914)

[Appendix Figure 11. Sensitivity analysis: Cost-effectiveness for QALY (complete cases) 33](#_Toc211862915)

[Appendix Figure 12. Sensitivity analysis: Cost-effectiveness for YFC (complete cases) 34](#_Toc211862916)

[Appendix Figure 13. Sensitivity analysis: Cost-effectiveness for CWLY (complete cases) 35](#_Toc211862917)

[Appendix Figure 14. Sensitivity analysis: Cost-effectiveness for QALY (alternative cost) 36](#_Toc211862918)

[Appendix Figure 15. Sensitivity analysis: Cost-effectiveness for YFC (alternative cost) 37](#_Toc211862919)

[Appendix Figure 16. Sensitivity analysis: Cost-effectiveness for CWLY (alternative cost) 38](#_Toc211862920)

[Appendix Figure 17. Sensitivity analysis: Cost-effectiveness for QALY (alternative outcome) 39](#_Toc211862921)

[Appendix Figure 18. Sensitivity analysis: Cost-effectiveness for YFC (alternative outcome) 40](#_Toc211862922)

[Appendix Figure 19. Sensitivity analysis: Cost-effectiveness for CWLY (alternative outcome) 41](#_Toc211862923)

[Appendix Figure 20. Sensitivity analysis: Cost-effectiveness for QALY over 12 weeks (current pramipexole cost) 42](#_Toc211862924)

[Appendix Figure 21. Sensitivity analysis: Cost-effectiveness for QALY over 48 weeks (current pramipexole cost) 43](#_Toc211862925)

## Supplementary references

1. Edwards SJ et al. Lithium or an atypical antipsychotic drug in the management of treatment-resistant depression: a systematic review and economic evaluation. Health Technol Assess. 2013;17(54):1-190. doi: 10.3310/hta17540.
2. Olgiati P et al. Challenging sequential approach to treatment resistant depression: cost-utility analysis based on the Sequenced Treatment Alternatives to Relieve Depression (STAR(⁎)D) trial. Eur Neuropsychopharmacol. 2013;23(12):1739-46. doi: 10.1016/j.euroneuro.2013.08.008.
3. Hollinghurst S et al. Cost-effectiveness of cognitive-behavioural therapy as an adjunct to pharmacotherapy for treatment-resistant depression in primary care: economic evaluation of the CoBalT Trial. Br J Psychiatry. 2014;204(1):69-76. doi: 10.1192/bjp.bp.112.125286.
4. Town JM et al. Efficacy and cost-effectiveness of intensive short-term dynamic psychotherapy for treatment resistant depression: 18-Month follow-up of the Halifax depression trial. J Affect Disord. 2020;273:194-202. doi: 10.1016/j.jad.2020.04.035.
5. Koeser L et al. Cost-effectiveness of long-term psychoanalytic psychotherapy for treatment-resistant depression: RCT evidence from the Tavistock Adult Depression Study (TADS). J Affect Disord. 2023;335:313-321. doi: 10.1016/j.jad.2023.04.109.
6. Nguyen KH, Gordon LG. Cost-Effectiveness of Repetitive Transcranial Magnetic Stimulation versus Antidepressant Therapy for Treatment-Resistant Depression. Value Health. 2015;18(5):597-604. doi: 10.1016/j.jval.2015.04.004.
7. Ross EL, Soeteman DI. Cost-Effectiveness of Esketamine Nasal Spray for Patients With Treatment-Resistant Depression in the United States. Psychiatr Serv. 2020;71(10):988-997. doi: 10.1176/appi.ps.201900625.
8. Bilbao A et al. Psychometric properties of the EQ-5D-5L in patients with major depression: factor analysis and Rasch analysis. J Ment Health. 2022;31(4):506-516. doi: 10.1080/09638237.2021.1875422.
9. Mitchell PM et al. Assessing the validity of the ICECAP-A capability measure for adults with depression. BMC Psychiatry. 2017;17(1):46. doi: 10.1186/s12888-017-1211-8.
10. Vergunst F et al. Psychometric validation of a multi-dimensional capability instrument for outcome measurement in mental health research (OxCAP-MH). Health Qual Life Outcomes. 2017;15(1):250. doi: 10.1186/s12955-017-0825-3.
11. Tundo A et al. Efficacy and safety of 24-week pramipexole augmentation in patients with treatment resistant depression.A retrospective cohort stud.Prog Neuropsychopharmacol Biol Psychiatry.2022;112:110425. doi:10.1016/j.pnpbp.2021.110425
12. Cusin C, Iovieno N, Iosifescu DV, Nierenberg AA, Fava M, Rush AJ, Perlis RH. A randomized, double-blind, placebo-controlled trial of pramipexole augmentation in treatment-resistant major depressive disorder. J Clin Psychiatry. 2013 Jul;74(7):e636-41. doi: 10.4088/JCP.12m08093.
13. Fawcett J, Rush AJ, Vukelich J, Diaz SH, Dunklee L, Romo P, Yarns BC, Escalona R. Clinical Experience With High-Dosage Pramipexole in Patients With Treatment-Resistant Depressive Episodes in Unipolar and Bipolar Depression. Am J Psychiatry. 2016 Feb 1;173(2):107-11. doi: 10.1176/appi.ajp.2015.15060788.

## Tables

Appendix Table 1. CHEERS 2022 Checklist

| **Topic** | **No.** | **Item** | **Location where item is reported** |
| --- | --- | --- | --- |
| **Title** |  |  |  |
|  | 1 | Identify the study as an economic evaluation and specify the interventions being compared. | Reported in the title |
| **Abstract** |  |  |  |
|  | 2 | Provide a structured summary that highlights context, key methods, results, and alternative analyses. | Reported in the abstract |
| **Introduction** |  |  |  |
| **Background and objectives** | 3 | Give the context for the study, the study question, and its practical relevance for decision making in policy or practice. | Paragraphs 1-2 in the Introduction, page 3 |
| **Methods** |  |  |  |
| **Health economic analysis plan** | 4 | Indicate whether a health economic analysis plan was developed and where available. | 1^st^ paragraph in the Methods (Study design), page 4 |
| **Study population** | 5 | Describe characteristics of the study population (such as age range, demographics, socioeconomic, or clinical characteristics). | Table 1, Appendix Table 5 |
| **Setting and location** | 6 | Provide relevant contextual information that may influence findings. | 2^nd^ paragraph in the Methods (Participants and procedures), page 4 |
| **Comparators** | 7 | Describe the interventions or strategies being compared and why chosen. | Paragraphs 1 and 2 in the Methods (Study design & Participants and procedures), page 4 |
| **Perspective** | 8 | State the perspective(s) adopted by the study and why chosen. | 1^st^ paragraph in the Methods (Study design), page 4 |
| **Time horizon** | 9 | State the time horizon for the study and why appropriate. | 1^st^ paragraph in the Methods (Study design), page 4 |
| **Discount rate** | 10 | Report the discount rate(s) and reason chosen. | 5^th^ paragraph in the Methods (Statistical analysis), page 6 |
| **Selection of outcomes** | 11 | Describe what outcomes were used as the measure(s) of benefit(s) and harm(s). | 3^rd^ paragraph in the Methods (Outcomes), page 4-5 |
| **Measurement of outcomes** | 12 | Describe how outcomes used to capture benefit(s) and harm(s) were measured. | 3^rd^ paragraph in the Methods (Outcomes), page 4-5 |
| **Valuation of outcomes** | 13 | Describe the population and methods used to measure and value outcomes. | 3^rd^ paragraph in the Methods (Outcomes), page 4-5 |
| **Measurement and valuation of resources and costs** | 14 | Describe how costs were valued. | 4^th^ paragraph in the Methods (Costs), page 5 |
| **Currency, price date, and conversion** | 15 | Report the dates of the estimated resource quantities and unit costs, plus the currency and year of conversion. | 4^th^ paragraph in the Methods (Costs), page 5, Appendix Table 2 |
| **Rationale and description of model** | 16 | If modelling is used, describe in detail and why used. Report if the model is publicly available and where it can be accessed. | not applicable |
| **Analytics and assumptions** | 17 | Describe any methods for analysing or statistically transforming data, any extrapolation methods, and approaches for validating any model used. | 5^th^ paragraph in the Methods (Statistical analysis), page 6 |
| **Characterising heterogeneity** | 18 | Describe any methods used for estimating how the results of the study vary for subgroups. | not applicable |
| **Characterising distributional effects** | 19 | Describe how impacts are distributed across different individuals or adjustments made to reflect priority populations. | not applicable |
| **Characterising uncertainty** | 20 | Describe methods to characterise any sources of uncertainty in the analysis. | Paragraphs 5 and 6 in the Methods (Statistical analysis & Sensitivity analyses), page 6-7 |
| **Approach to engagement with patients and others affected by the study** | 21 | Describe any approaches to engage patients or service recipients, the general public, communities, or stakeholders (such as clinicians or payers) in the design of the study. | 2^nd^ paragraph in the Methods (Participants and procedures), page 4 |
| **Results** |  |  |  |
| **Study parameters** | 22 | Report all analytic inputs (such as values, ranges, references) including uncertainty or distributional assumptions. | Results, page 7-8, Tables 2-4, Appendix Tables 6-11 |
| **Summary of main results** | 23 | Report the mean values for the main categories of costs and outcomes of interest and summarise them in the most appropriate overall measure. | Paragraphs 2-4 in the Results, page 7-8 |
| **Effect of uncertainty** | 24 | Describe how uncertainty about analytic judgments, inputs, or projections affect findings. Report the effect of choice of discount rate and time horizon, if applicable. | 4^th^ paragraph in the Results (Cost-effectiveness results), page 8, Appendix Tables 9-10, Appendix Figures 8-21) |
| **Effect of engagement with patients and others affected by the study** | 25 | Report on any difference patient/service recipient, general public, community, or stakeholder involvement made to the approach or findings of the study | not applicable |
| **Discussion** |  |  |  |
| **Study findings, limitations, generalisability, and current knowledge** | 26 | Report key findings, limitations, ethical or equity considerations not captured, and how these could affect patients, policy, or practice. | Paragraphs 1-5 in the Discussion, page 9-10 |
| **Other relevant information** |  |  |  |
| **Source of funding** | 27 | Describe how the study was funded and any role of the funder in the identification, design, conduct, and reporting of the analysis | Reported on page 12 |
| **Conflicts of interest** | 28 | Report authors conflicts of interest according to journal or International Committee of Medical Journal Editors requirements. | Reported on page 11 |

Source: Husereau D, Drummond M, Augustovski F, et al. Consolidated Health Economic Evaluation Reporting Standards 2022 (CHEERS 2022) Explanation and Elaboration: A Report of the ISPOR CHEERS II Good Practices Task Force. Value Health 2022;25. <doi:10.1016/j.jval.2021.10.008>

Appendix Table 2. Unit costs (in £ for year 2022/2023)

| **Resource use** | **Unit cost (£)** | **Unit of measurement** | **Source of estimate** |
| --- | --- | --- | --- |
| Pramipexole 1mg | £0·07  £0·52 | per prescribed daily dose | Prescription Cost Analysis - England 2022/2023^1^ & 2024/2025^2^ |
| Pramipexole 0.25mg | £0·10  £0·19 | per prescribed daily dose | Prescription Cost Analysis - England 2022/2023^1^  & 2024/2025^2^ |
| NHS prescription fee | £2·17 | per prescription | Expert advice |
| Antidepressants | £0·02-£0·69 | per prescribed dose | Prescription Cost Analysis - England 2022/2023^1^ |
| Other MH medication | £0·02-£1·22 | per prescribed dose | Prescription Cost Analysis - England 2022/2023^1^ |
| NMH medication | £0·01-£4·53 | per prescribed dose | Prescription Cost Analysis - England 2022/2023^1^ |
| General medical ward | £392·47 | per day | Scottish Costs Book 2022/23^3^ |
| General surgical wards | £1,029·26 | per day | Scottish Costs Book 2022/23^3^ |
| Psychiatric outpatient department (hospital) visits | £231·00 | per contact | National Schedule of NHS Costs Year: 2021/22^4^ |
| Trial clinician face-to-face contact | £90·63 | per 20 min contact | National Schedule of NHS Costs Year: 2021/22^4^ |
| Trial clinician phone contact | £40·78 | per 15 min contact | National Schedule of NHS Costs Year: 2021/22^4^ |
| Psychiatric outpatient contact (public sector) | £150·59 | per contact | National Schedule of NHS Costs Year: 2021/22^4^ |
| Psychiatric outpatient contact (private sector) | £180·00 | per contact (30 min) | https://psychiatry-uk.com/fees/ |
| Other hospital outpatient visits | £225·95 | per contact | National Schedule of NHS Costs Year: 2021/22 (Total Outpatient Attendances Data, except: 180 Accident & Emergency & 722 Liaison Psychiatry)^4^ |
| A&E visit | £144·00 | per contact | National Schedule of NHS Costs Year: 2021/22 (Total Outpatient Attendances Data, Service Code 180 Accident & Emergency)^4^ |
| Psychologist outpatient contact (public sector) | £208·49 | per contact | National Schedule of NHS Costs Year: 2021/22^4^ |
| Psychologist outpatient contact (private sector) | £270·00 | per contact | https://theprivatetherapyclinic.co.uk/fees/ |
| Community psychiatric nurse/Case manager | £68·00 | per hour | PSSRU Unit Cost of Health and Social Care 2023^5^ |
| Community mental health centre | £61·00 | per contact | PSSRU Unit Cost of Health and Social Care 2023^5^ |
| Group therapy | £21·00 | per session | PSSRU Unit Cost of Health and Social Care 2023^5^ |
| Specialist education | £22·00 | per client contact | PSSRU Unit Cost of Health and Social Care 2023^5^ |
| Self-help/support group | £17·00 | per contact | PSSRU Unit Cost of Health and Social Care 2023^5^ |
| Community/district nurse/Case | £47·00 | per hour | PSSRU Unit Cost of Health and Social Care 2023^5^ |
| Occupational therapy | £106·00 | per contact | PSSRU Unit Cost of Health and Social Care 2023^5^ |
| Alternative practice (public sector) | £107·89 | per contact | National Schedule of NHS Costs Year: 2021/22^4^ |
| Alternative practice (private sector) | £67·00 | per contact | https://www.surreyhillsacupuncture.co.uk/treatment-fees/ |
| Emergency services/ Ambulance | £143·74 | per contact | National Schedule of NHS Costs Year: 2021/22^4^ |
| General practitioner (public sector) | £42·00 | per surgery consultation lasting 10 minutes | PSSRU Unit Cost of Health and Social Care 2023^5^ |
| General practitioner (private sector) | £90·00 | per surgery consultation lasting 20 minutes | https://www.theprivategpclinic.co.uk/our-fees/ |
| Primary care practice nurse (public sector) | £47·00 | per hour | PSSRU Unit Cost of Health and Social Care 2023^5^ |
| Primary care practice nurse (private sector) | £45·00 | per consultation up to 15 min | https://www.theprivategpclinic.co.uk/our-fees/ |
| Informal care | £27·00 | per hour | PSSRU Unit Cost of Health and Social Care 2023^5^ |
| Absenteeism | £21·65 | per working hour | Office for National Statistics. Employee earnings in the UK. Table 1.5a Hourly pay - Gross (£) - For all employee jobs: United Kingdom |
| Inflation index |  |  | NHS Cost Inflation Index (NHSCII); PSSRU Unit Cost of Health and Social Care 2023^5^ |

Note: MH - Mental health, NMH - Non-mental health; ^1^ NHS. Prescription Cost Analysis – England – 2022-23. 2023;^2^ NHS. Prescription Cost Analysis – England – 2024-25. 2025 ^3^ Public Health Scotland. Scottish health service costs. Summary for financial year 2022/23. Public Health Scotland 2023; ^4^ NHS. National Cost Collection: National schedule of NHS costs - Year 2021/22. NHS trusts and NHS foundation trusts. 2022; ^5^ Jones KC, Weatherly H, Birch S, Castelli A, Chalkley M, Dargan A, et al. Unit Costs of Health and Social Care 2022 Manual. Technical report. Personal Social Services Research Unit (University of Kent) & Centre for Health Economics (University of York); 2023. doi:10.22024/UniKent/01.02.100519.

Appendix Table 3. Data availability by treatment arm (n=124)

|  | Baseline  n (%) complete | | 12 weeks  n (%) complete | | 24 weeks  n (%) complete | | 36 weeks  n (%) complete | | 48 weeks  n (%) complete | | Available data across all time points  (%) | |
| --- | --- | --- | --- | --- | --- | --- | --- | --- | --- | --- | --- | --- |
|  | Pramipexole  (n=61) | Placebo  (n=63) | Pramipexole  (n=61) | Placebo  (n=63) | Pramipexole  (n=61) | Placebo  (n=63) | Pramipexole  (n=61) | Placebo  (n=63) | Pramipexole  (n=61) | Placebo  (n=63) | Pramipexole  (n=61) | Placebo  (n=63) |
| EQ-5D-5L | 61 (100%) | 63 (100%) | 61 (100%) | 63 (100%) | 46 (75%) | 44 (70%) | 40 (66%) | 35 (56%) | 33 (54%) | 36 (57%) | 79% | 76.6% |
| EQ VAS | 61 (100%) | 63 (100%) | 61 (100%) | 63 (100%) | 45 (74%) | 44 (70%) | 40 (66%) | 35 (56%) | 33 (54%) | 36 (57%) | 78.8% | 76.6% |
| ICECAP-A | 61 (100%) | 63 (100%) | 61 (100%) | 63 (100%) | 46 (75%) | 44 (70%) | 40 (66%) | 35 (56%) | 33 (54%) | 36 (57%) | 79% | 76.6% |
| OxCAP-MH | 61 (100%) | 63 (100%) | 61 (100%) | 63 (100%) | 46 (75%) | 43 (68%) | 40 (66%) | 35 (56%) | 33 (54%) | 36 (57%) | 79% | 76.2% |
| HEQ | 61 (100%) | 63 (100%) | 61 (100%) | 63 (100%) | 46 (75%) | 43 (68%) | 40 (66%) | 35 (56%) | 32 (52%) | 35 (56%) | 78.6% | 76% |

Note: HEQ – Health Economic Questionnaire

**Extent and patterns of missing data**

All participants (n=124) had complete baseline and 12-week follow-up data across all health economic outcomes. Missingness emerged from 24-week follow-up onwards, and increased progressively over time. The level of overall missingness across all outcomes and time points was 22%. The pattern of missingness was broadly comparable across treatment arms and outcome measures, and was characterised predominantly by attrition over time rather than sporadic item-level non-response, suggesting a largely monotone missing data structure. There were no systematic differences in missingness patterns between treatment groups.

### Appendix Table 4. Baseline characteristics of the full randomised sample, health economic sample, and complete cases sample

|  | **Full randomized sample** | | | | **Health economic sample (main analysis)** | | | | **Complete cases sample** | | | |
| --- | --- | --- | --- | --- | --- | --- | --- | --- | --- | --- | --- | --- |
|  | **Pramipexole**  **(n=75)** | | **Placebo**  **(n=75)** | | **Pramipexole**  **(n=61)** | | **Placebo**  **(n=63)** | | **Pramipexole**  **(n=29)** | | **Placebo**  **(n=27)** | |
|  | n | % or Mean (SD) | n | % or Mean (SD) | n | % or Mean (SD) | n | % or Mean (SD) | n | % or Mean (SD) | n | % or Mean (SD) |
| **Sex** |  |  |  |  |  |  |  |  |  |  |  |  |
| Female | 41 | 55% | 43 | 57% | 33 | 54% | 37 | 59% | 16 | 55% | 14 | 52% |
| Male | 34 | 45% | 32 | 43% | 28 | 46% | 26 | 41% | 13 | 45% | 13 | 48% |
| **Age** | 75 | 44·0 (15·2) | 75 | 46·0 (12·7) | 61 | 43·7 (15·0) | 63 | 45·6 (12·6) | 29 | 40·3 (13·8) | 27 | 44·1 (13·8) |
| **QIDS-SR16** | 75 | 16·4 (3·4) | 75 | 16·2 (3·5) | 61 | 16·3 (3·4) | 63 | 15·6 (3·1) | 29 | 16·5 (3·6) | 27 | 15·3 (2·4) |
| **HE outcomes** |  |  |  |  |  |  |  |  |  |  |  |  |
| EQ-5D-5L utility index | 74 | 0·47 (0·25) | 75 | 0·47 (0·24) | 61 | 0·50 (0·23) | 63 | 0·49 (0·23) | 29 | 0·49 (0·20) | 27 | 0·49 (0·25) |
| EQ VAS | 74 | 52·3 (19·6) | 75 | 55·4 (18·2) | 61 | 52·1 (19·2) | 63 | 56·8 (17·6) | 29 | 51·1 (20·3) | 27 | 56·8 (13·8) |
| OxCAP-MH score | 75 | 55·0 (12·6) | 75 | 56·1 (11·4) | 61 | 56·9 (12·5) | 63 | 56·9 (10·9) | 29 | 57·2 (11·4) | 27 | 56·2 (11·1) |
| ICECAP-A index | 75 | 0·45 (0·16) | 75 | 0·45 (0·15) | 61 | 0·46 (0·16) | 63 | 0·47 (0·15) | 29 | 0·48 (0·15) | 27 | 0·49 (0·15) |

Note: QIDS-SR16 - Quick Inventory of Depressive Symptomatology self-report version 16; HE – health economics; EQ-5D-5L - European Quality of Life 5 Dimensions 5 Level; EQ VAS - European Quality of Life Visual Analogue Scale, ICECAP-A - ICEpop CAPability measure for Adults; OxCAP-MH - Oxford Capability Questionnaire – Mental Health

Appendix Table 5. Sensitivity analysis sample characteristics at baseline

|  | **PP sample imputed (48 weeks)** | | | | **Complete cases sample (48 weeks)** | | | |
| --- | --- | --- | --- | --- | --- | --- | --- | --- |
| **Participant characteristics at baseline** | **Pramipexole**  **(n=48)** | | **Placebo**  **(n=55)** | | **Pramipexole**  **(n=29)** | | **Placebo**  **(n=27)** | |
|  | n | % or Mean (SD) | n | % or Mean (SD) | n | % or Mean (SD) | n | % or Mean (SD) |
| **Sex** |  |  |  |  |  |  |  |  |
| Female | 27 | 56% | 20 | 36% | 16 | 55% | 14 | 52% |
| Male | 21 | 44% | 35 | 64% | 13 | 45% | 13 | 48% |
| **Age** | 48 | 44·7 (15·1) | 55 | 45·1 (12·4) | 29 | 40·3 (13·8) | 27 | 44·1 (13·8) |
| **Depression severity (QIDS-SR16)** |  |  |  |  |  |  |  |  |
| Mild (<11) | 0 | 0% | 0 | 0% | 0 | 0% | 0 | 0% |
| Moderate (11 – 15) | 22 | 46% | 29 | 53% | 11 | 38% | 14 | 52% |
| Severe (16-20) | 22 | 46% | 22 | 40% | 15 | 52% | 13 | 48% |
| Very severe (>20) | 4 | 8% | 4 | 7% | 3 | 10% | 0 | % |
| **Accommodation** |  |  |  |  |  |  |  |  |
| Owner occupied/privately rented accommodation | 40 | 83% | 48 | 87% | 27 | 93% | 24 | 89% |
| Housing association/local authority accommodation | 8 | 17% | 6 | 11% | 2 | 7% | 1 | 4% |
| Residential facilities | 0 | 0% | 1 | 2% | 0 | 0% | 0 | 0% |
| Other | 0 | 0% | 0 | 0% | 0 | 0% | 2 | 7% |
| **Living Situation** |  |  |  |  |  |  |  |  |
| Living alone | 14 | 29% | 11 | 20% | 7 | 24% | 5 | 18% |
| Living with others | 34 | 71% | 44 | 80% | 22 | 76% | 22 | 78% |
| **Employment** |  |  |  |  |  |  |  |  |
| Employed, self-employed or voluntary employed | 30 | 63% | 32 | 56% | 18 | 63% | 15 | 56% |
| Unemployed | 7 | 15% | 8 | 15% | 5 | 17% | 5 | 18% |
| Housewife/-husband | 2 | 4% | 2 | 4% | 1 | 3% | 1 | 4% |
| Student | 1 | 2% | 3 | 6% | 2 | 7% | 1 | 4% |
| Retired | 6 | 12% | 7 | 13% | 2 | 7% | 5 | 18% |
| Other | 2 | 4% | 3 | 6% | 1 | 3% | 0 | 0% |
| **Quality-of-life and capability wellbeing** |  |  |  |  |  |  |  |  |
| EQ-5D-5L utility index | 48 | 0·50 (0·21) | 55 | 0·51 (0·23) | 29 | 0·49 (0·20) | 27 | 0·49 (0·25) |
| EQ-5D VAS | 48 | 52·77 (19·07) | 55 | 57·54 (17·71) | 29 | 51·13 (20·27) | 27 | 56·85 (13·84) |
| OxCAP-MH score | 48 | 57·87 (11·54) | 55 | 57·92 (10·15) | 29 | 57·21 (11·39) | 27 | 56·19 (11·12) |
| ICECAP-A index | 48 | 0·48 (0·15) | 55 | 0·48 (0·15) | 29 | 0·48 (0·15) | 27 | 0·49 (0·15) |

Note: PP - Per protocol (participants who remained on the allocated treatment); QIDS-SR16 - Quick Inventory of Depressive Symptomatology-Self Report version 16; HE – health economics; EQ-5D-5L - European Quality of Life 5 Dimensions 5 Level; EQ VAS - European Quality of Life Visual Analogue Scale, ICECAP-A - ICEpop CAPability measure for Adults; OxCAP-MH - Oxford Capability Questionnaire – Mental Health

Appendix Table 6. Health outcome measure results over time (n=124)

| **Outcome measure** | **Baseline** | **Trial week 12** | **Trial week 24** | **Trial week 36** | **Trial week 48** |
| --- | --- | --- | --- | --- | --- |
| **EQ-5D-5L utility index observed** |  |  |  |  |  |
| **Pramipexole**  Mean (SD) [no. of obs] | 0·503 (0·229) [n=61] | 0·640 (0·233) [n=61] | 0·668 (0·195) [n=45] | 0·687 (0·230) [n=40] | 0·586 (0·291) [n=33] |
| **Placebo**  Mean (SD) [no. of obs] | 0·494 (0·231) [n=63] | 0·535 (0·235) [n=63] | 0·544 (0·257) [n=44] | 0·589 (0·250) [n=35] | 0·526 (0·248) [n=36] |
| **Difference**  Mean difference [95%CI] | 0·009  [-0·072 to 0·091] | 0·105*  [0·021 to 0·188] | 0·127**  [0·127 to 0·223] | 0·097  [-0·013 to 0·208] | 0·059  [-0·070 to 0·189] |
| **EQ-5D-5L utility index imputed** |  |  |  |  |  |
| **Pramipexole**  Mean (SE) [no. of obs] |  |  | 0·666 (0·026)  [n=61] | 0·696 (0·030)  [n=61] | 0·617 (0·039)  [n=61] |
| **Placebo**  Mean (SE) [no. of obs] |  |  | 0·543 (0·034)  [n=63] | 0·563 (0·036)  [n=63] | 0·543 (0·037)  [n=63] |
| **Difference**  Mean difference [95%CI] |  |  | 0·123**  [0·035 to 0·211] | 0·132**  [0·037 to 0·226] | 0·064  [-0·037 to 0·166] |
| **EQ-5D VAS observed** |  |  |  |  |  |
| **Pramipexole**  Mean (SD) [no. of obs] | 52·08 (19·19)  [n=61] | 61·01 (19·38)  [n=61] | 59·48 (20·30)  [n=45] | 63·77 (19·35)  [n=40] | 61·06 (17·92)  [n=33] |
| **Placebo**  Mean (SD) [no. of obs] | 56·84 (17·58)  [n=63] | 56·14 (18·98)  [n=63] | 55·36 (18·90)  [n=44] | 63·2 (17·78)  [n=35] | 58·5 (19·01)  [n=36] |
| **Difference**  Mean difference [95%CI] | -4·75  [-11·29 to 1·78] | 4·87  [-1·94 to 11·69] | 4·12  [-4·14 to 12·39] | 0·57  [-8·02 to 9·17] | 2·56  [-6·33 to 11·46] |
| **EQ-5D VAS imputed** |  |  |  |  |  |
| **Pramipexole**  Mean (SE) [no. of obs] |  |  | 59·80 (3·08)  [n=61] | 62·69 (2·87)  [n=61] | 62·27 (2·38)  [n=61] |
| **Placebo**  Mean (SE) [no. of obs] |  |  | 54·36 (2·57)  [n=63] | 61·75 (2·61)  [n=63] | 58·56 (2·92)  [n=63] |
| **Difference**  Mean difference [95%CI] |  |  | 5·16  [-3·11 to 13·44] | 0·93  [-6·99 to 8·87] | 3·71  [-3·92 to 11·34] |
| **ICECAP-A index observed** |  |  |  |  |  |
| **Pramipexole**  Mean (SD) [no. of obs] | 0·463 (0·162)  [n=61] | 0·593 (0·217)  [n=61] | 0·614 (0·225)  [46] | 0·636 (0·208)  [40] | 0·626 (0·202)  [33] |
| **Placebo**  Mean (SD) [no. of obs] | 0·467 (0·146)  [n=63] | 0·516 (0·205)  [n=63] | 0·555 (0·198)  [44] | 0·591 (0·200)  [35] | 0·558 (0·226)  [36] |
| **Difference**  Mean difference [95%CI] | -0·004  [-0·059 to 0·050] | 0·077*  [0·002 to 0·152] | 0·058  [-0·030 to 0·148] | 0·044  [-0·050 to 0·138] | 0·067  [-0·036 to 0·171] |
| **ICECAP-A index imputed** |  |  |  |  |  |
| **Pramipexole**  Mean (SE) [no. of obs] |  |  | 0·622 (0·029)  [n=61] | 0·646 (0·028)  [n=61] | 0·639 (0·029)  [n=61] |
| **Placebo**  Mean (SE) [no. of obs] |  |  | 0·553 (0·025)  [n=63] | 0·587 (0·026)  [n=63] | 0·567 (0·030)  [n=63] |
| **Difference**  Mean difference [95%CI] |  |  | 0·074  [-0·007 to 0·156] | 0·050  [-0·035 to 0·137] | 0·075  [-0·017 to 0·168] |
| **OxCAP-MH score observed** |  |  |  |  |  |
| **Pramipexole**  Mean (SD) [no. of obs] | 56·89 (12·54)  [n=61] | 65·98 (13·84)  [n=61] | 66·23 (14·19)  [46] | 67·46 (13·99)  [40] | 64·72 (15·42)  [33] |
| **Placebo**  Mean (SD) [no. of obs] | 56·94 (10·90)  [n=63] | 60·76 (12·42)  [n=63] | 60·79 (13·43)  [43] | 63·12 (12·93)  [35] | 61·54 (13·70)  [36] |
| **Difference**  Mean difference [95%CI] | -0·05  [-4·22 to 4·11] | 5·21*  [0·54 to 9·89] | 5·44  [-0·38 to 11·27] | 4·33  [-1·89 to 10·56] | 3·18  [-3·81 to 10·17] |
| **OxCAP-MH score imputed** |  |  |  |  |  |
| **Pramipexole**  Mean (SE) [no. of obs] |  |  | 65·20 (1·91)  [n=61] | 66·71 (1·92)  [n=61] | 64·82 (1·93)  [n=61] |
| **Placebo**  Mean (SE) [no. of obs] |  |  | 60·95 (1·73)  [n=63] | 62·09 (1·86)  [n=63] | 61·89 (1·85)  [n=63] |
| **Difference**  Mean difference [95%CI] |  |  | 4·25  [-0·84 to 9·34] | 4·62  [-0·68 to 9·92] | 2·93  [-2·34 to 8·20] |

Note: SD – standard deviation; SE – standard error; CI – confidence interval; EQ-5D-5L - European Quality of Life 5 Dimensions 5 Level; EQ VAS - European Quality of Life Visual Analogue Scale, ICECAP-A - ICEpop CAPability measure for Adults; OxCAP-MH - Oxford Capability Questionnaire – Mental Health; *p<0·05, **p<0·01, ***p<0·001

Appendix Table 7. Resource use results: available cases

|  | **Weeks -12 - 0** | | | | **Weeks 1 - 12** | | | | **Weeks 13-24** | | | | **Weeks 25-36** | | | | **Weeks 37-48** | | | |
| --- | --- | --- | --- | --- | --- | --- | --- | --- | --- | --- | --- | --- | --- | --- | --- | --- | --- | --- | --- | --- |
|  | **Pramipexole**  **61** | | **Placebo**  **63** | | **Pramipexole**  **61** | | **Placebo**  **63** | | **Pramipexole**  **46** | | **Placebo**  **43** | | **Pramipexole**  **40** | | **Placebo**  **42** | | **Pramipexole**  **32** | | **Placebo**  **35** | |
| **Resource use** | Mean (SD) | n of users | Mean (SD) | n of users | Mean (SD) | n of users | Mean (SD) | n of users | Mean (SD) | n of users | Mean (SD) | n of users | Mean (SD) | n of users | Mean (SD) | n of users | Mean (SD) | n of users | Mean (SD) | n of users |
| **Medication** |  |  |  |  |  |  |  |  |  |  |  |  |  |  |  |  |  |  |  |  |
| Antidepressant drugs |  | 57 |  | 59 |  | 55 |  | 60 |  | 45 |  | 47 |  | 37 |  | 41 |  | 30 |  | 32 |
| Other MH medication |  | 10 |  | 13 |  | 10 |  | 14 |  | 7 |  | 9 |  | 7 |  | 9 |  | 6 |  | 9 |
| NMH medication |  | 34 |  | 41 |  | 38 |  | 44 |  | 31 |  | 29 |  | 24 |  | 24 |  | 20 |  | 21 |
| **Inpatient services** |  |  |  |  |  |  |  |  |  |  |  |  |  |  |  |  |  |  |  |  |
| MH inpatient | 0 | 0 | 0 | 0 | 0 | 0 | 0 | 0 | 0 | 0 | 0 | 0 | 0 | 0 | 0 | 0 | 0 | 0 | 0 | 0 |
| NMH Medical wards^c^ | 0·02 (0·13) | 1 | 0·05 (0·28) | 2 | 0·02 (0·13) | 1 | 0·02 (0·13) | 1 | - | 0 | 0·09 (0·61) | 1 | 0·05 (0·32) | 1 | - | 0 | 0·06 (0·35) | 1 | - | 0 |
| NMH Surgical wards^c^ | 0·02 (0·13) | 1 | 0·02 (0·13) | 1 | 0·02 (0·13) | 1 | - | 0 | 0·07 (0·33) | 2 | 0·02 (0·15) | 1 | 0·03 (0·16) | 1 | - | 0 | 0·13 (0·34) | 4 | 0·06 (0·24) | 2 |
| NMH Other hospital ward^c^ | 0 | 0 | 0·05 (0·39) | 1 | 0·03 (0·26) | 1 | 0·03 (0·25) | 1 | - | 0 | - | 0 | 0·08 (0·35) | 2 | 0·03 (0·17) | 1 | 0·31 (1·12) | 3 | - | 0 |
| **MH outpatient services** |  |  |  |  |  |  |  |  |  |  |  |  |  |  |  |  |  |  |  |  |
| Psychiatric outpatient service^a^ | 0·34 (0·98) | 9 | 0·32 (1·57) | 6 | - | 0 | 0·02 (0·13) | 1 | 0·02 (0·15) | 1 | 0·12 (0·63) | 2 | - | 0 | - | 0 | 0·25 (1·41) | 1 | 0·2 (0·87) | 3 |
| Psychiatrist^a^ | 0·44 (1·43) | 9 | 0·16 (0·55) | 6 | - | 0 | - | 0 | 0·02 (0·15) | 1 | - | 0 | - | 0 | 0·03 (0·17) | 1 | 0·06 (0·35) | 1 | 0·06 (0·24) | 2 |
| **NMH outpatient services** |  |  |  |  |  |  |  |  |  |  |  |  |  |  |  |  |  |  |  |  |
| Hospital outpatient visits^a^ | 0·08 (0·65) | 1 | - | 0 | - | 0 | 0·03 (0·18) | 2 | - | 0 | - | 0 | 0·05 (0·22) | 2 | - | 0 | 0·13 (0·71) | 1 | 0·03 (0·17) | 1 |
| A&E visit^a^ | 0·02 (0·13) | 1 | 0·03 (0·18) | 2 | 0·03 (0·18) | 2 | - | 0 | 0·11 (0·53) | 2 | - | 0 | 0·08 (0·27) | 3 | 0·03 (0·17) | 1 | 0·06 (0·35) | 1 | 0·09 (0·28) | 3 |
| Other hospital outpatient service^a^ | 0·21 (0·66) | 8 | 0·52 (1·99) | 10 | 0·03 (0·26) | 1 | 0·02 (0·13) | 1 | - | 0 | 0·12 (0·63) | 2 | 0·18 (0·84) | 2 | 0·06 (0·24) | 2 | 0·25 (0·8) | 4 | 0·09 (0·37) | 2 |
| **MH-specific community care and day-care services** |  |  |  |  | - | 0 | - | 0 |  |  |  |  |  |  |  |  |  |  |  |  |
| Psychologist^a^ | 0·8 (2·61) | 7 | 0·5 (2·05) | 5 | - | 0 | - | 0 | - | 0 | - | 0 | - | 0 | - | 0 | 0·19 (1·06) | 1 | 0 | 0 |
| Psychiatric nurse^a^ | - | 0 | 0·05 (0·28) | 2 | - | 0 | 0·02 (0·13) |  | - | 0 | 0·09 (0·61) | 1 | - | 0 | - | 0 | - | 0 | 0·03 (0·17) | 1 |
| Community mental health centre^a^ | 0·16 (0·78) | 3 | 0·21 (1·27) | 4 | - | - | - | 0 | - | 0 | 0·07 (0·34) | 2 | - | 0 | - | 0 | - | 0 | 0·03 (0·17) | 1 |
| Group therapy^b^ | - | 0 | 0·19 (1·51) | 1 | - | - | - | 0 | - | - | - | 0 | - | 0 | - | 0 | - | 0 | - | 0 |
| **NMH-specific community care** |  |  |  |  |  |  |  |  |  |  |  |  |  |  |  |  |  |  |  |  |
| Community/district nurse^a^ | - | 0 | 0 |  | - | 0 | 0·02 (0·13) | 1 | - | 0 | - | 0 | - | 0 | - | 0 | - | 0 | - | 0 |
| Specialist education^a^ | - | 0 | 0·02 (0·13) | 1 | - | 0 | 0·05 (0·38) | 1 | - | 0 | 0·19 (1·22) | 1 | - | 0 | - | 0 | - | 0 | - | 0 |
| Self-help/support group^a^ | 0·69 (4·66) | 2 | 0·29 (1·4) | 4 | - | 0 | 0·02 (0·13) | 1 | 0·04 (0·29) | 1 | 0·23 (1·25) | 2 | 0·08 (0·47) | 1 | - | 0 | 0·16 (0·88) | 1 | - | 0 |
| Occupational therapy^a^ | 0·03 (0·26) | 1 | 0 |  | - | 0 | - | 0 | - | 0 | - | 0 | - | 0 | 0·03 (0·17) | 1 | - | 0 | - | 0 |
| Alternative practice^a^ | 0·13 (0·53) | 4 | 0·08 (0·45) | 2 | 0·03 (0·18) | 2 | 0·02 (0·13) | 1 | - | 0 | - | 0 | 0·13 (0·79) | 1 | - | 0 | 0·31 (1·77) | 1 | - | 0 |
| Emergency services^a^ | - | 0 | 0·02 (0·13) | 1 | - | 0 | - | 0 | 0·04 (0·29) | 1 | - | 0 | - | 0 | - | 0 | 0·06 (0·25) | 2 | - | 0 |
| Other community care^a^ | 0·62 (4·61) | 2 | 0·24 (1·55) | 2 | - | - | - | 0 | - | - | - | 0 | - | 0 | - | 0 | - | 0 | - | 0 |
| **Primary care** |  |  |  |  |  |  |  |  |  |  |  |  |  |  |  |  |  |  |  |  |
| GP^a^ | 0·89 (1·74) | 18 | 0·47 (0·9) | 17 | 0·02 (0·13) | 1 | 0·06 (0·3) | 3 | 0·02 (0·15) | 1 | 0·21 (0·74) | 4 | 0·25 (0·95) | 3 | 0·09 (0·37) | 2 | 0·5 (1·85) | 4 | 0·11 (0·4) | 3 |
| GP practice nurse^a^ | 0·07 (0·31) | 3 | 0·15 (0·51) | 6 | 0·02 (0·13) | 1 | 0·02 (0·13) | 1 | 0·13 (0·88) | 1 | 0·05 (0·3) | 1 | 0·1 (0·5) | 2 | - | 0 | 0·91 (4·43) | 3 | 0·06 (0·24) | 2 |
| **Social care** |  |  |  |  |  |  |  |  |  |  |  |  |  |  |  |  |  |  |  |  |
| Social worker^a^ | - | 0 | - | 0 | - | 0 | - | 0 | - | 0 | - | 0 | - | 0 | - | 0 | - | 0 | - | 0 |
| **Informal care** |  |  |  |  |  |  |  |  |  |  |  |  |  |  |  |  |  |  |  |  |
| Hours per week of informal care received | 8·66 (10·96) | 21 | 10·33 (13·71) | 30 | 6·06 (7·18) | 15 | 9·66 (12·19) | 15 | 5·10 (6·11) | 10 | 6·09 (4·54) | 11 | 187·80 (461·24) | 40 | 307·95 (734·38) | 35 | 428·69 (1156·83) | 32 | 1231·83 (3982·27) | 35 |
| **Lost productivity** |  |  |  |  |  |  |  |  |  |  |  |  |  |  |  |  |  |  |  |  |
| Absenteeism (days absent from work) | 28·61 (31·71) | 21 | 29·15 (31·87) | 19 | 19·00 (30·65) | 12 | 19·92 (29·60) | 13 | 30·25 (37·01) | 4 | 26·00 (35·42) | 12 | 35·25 (39·44) | 8 | 17·18 (27·79) | 8 | 28·6 (31·22) | 10 | 9·88 (9·34) | 9 |

Note: HEQ - health economic questionnaire, MH - Mental health, NMH - Non-mental health, NHS+PSS - National Health Service and Personal Social Services, GP - general practice, CI - Confidence Interval; ^a^ per contact, ^b^ per session, ^c^ per day

Appendix Table 8. Baseline cost results (in £, for year 2022/2023)

|  | **-12 to 0 weeks** | | | | |
| --- | --- | --- | --- | --- | --- |
|  | **Pramipexole**  **(n=61)** | | **Placebo**  **(n=63)** | |  |
|  | **Mean** | **SD** | **Mean** | **SD** | **Δ costs**  **[95% CI]** |
| **Medication** | 84·2 | 119·17 | 73·76 | 91·05 | 10·43  [-27·19 to 48·06] |
| Antidepressants | 43·92 | 89·07 | 22·84 | 32·62 | 21·08  [-2·61 to 44·78] |
| Other MH medication | 2·73 | 10·06 | 4 | 21·49 | -1·27  [-7·27 to 4·72] |
| NMH medication | 37·55 | 78·55 | 46·92 | 80·75 | -9·36  [-37·69 to 18·96] |
| **Inpatient care** | 23·31 | 140·25 | 35·03 | 233·07 | -11·71  [-80·37 to 56·93] |
| MH inpatient | 0 | 0 | 0 | 0 | - |
| NMH inpatient | 23·31 | 140·25 | 35·03 | 233·07 | -11·71  [-80·37 to 56·93] |
| **Outpatient care** | 196·69 | 425·66 | 212·99 | 561·65 | -16·30  [-193·89 to 161·29] |
| MH Outpatient care | 146·18 | 349·13 | 90·07 | 368·88 | 56·11  [-71·65 to 183·87] |
| NMH Outpatient care | 50·51 | 149·76 | 122·93 | 449·31 | -72·41  [-192·27 to 47·45] |
| **Community and community-based day services** | 127·77 | 405·25 | 111·71 | 318·04 | 16·05  [-113·22 to 145·33] |
| MH Community care | 92·03 | 366·47 | 82·7 | 299·45 | 9·32  [-109·47 to 128·12] |
| NMH Community care | 35·74 | 183·8 | 29·01 | 112·47 | 6·72  [-47·24 to 60·70] |
| **Primary care** | 40·26 | 75·26 | 23·14 | 46·96 | 17·11  [-5·10 to 39·34] |
| **NHS+PSS Perspective** | 472·22 | 650·72 | 456·63 | 846·65 | 15·59  [-253·46 to 284·65] |
| Healthcare use in the private sector | 35·46 | 144·9 | 59·4 | 184·46 | -23·94  [-83·03 to 35·15] |
| Absenteeism | 438·89 | 1383·26 | 186·26 | 624·72 | 252·63  [-126·92 to 632·19] |
| Informal care | 1046·36 | 2501·59 | 1676·57 | 3508·72 | -630·21  [-1716·61 to 456·19] |
| **Societal perspective** | 1992·93 | 3155·5 | 2378·86 | 3618·99 | -385·92  [-1594·56 to 822·71] |

Note: MH - Mental health, NMH - Non-mental health, NHS+PSS - National Health Service and Personal Social Services, GP – general practice, CI - Confidence Interval

Appendix Table 9. Sensitivity analyses: Imputed EQ-5D-5L, EQ VAS, ICECAP-A, OxCAP-MH results for per protocol (PP) sample (n=103) and complete cases sample (n=56)

| **Outcome measure** | **Baseline** | **Trial week 12** | **Trial week 24** | **Trial week 36** | **Trial week 48** |
| --- | --- | --- | --- | --- | --- |
| **PP sample (n=103)** | | | | | |
| **EQ-5D-5L utility index** |  |  |  |  |  |
| **Pramipexole**  Mean (SD) [n=48] | 0·50 (0·21)  [n=48] | 0·66 (0·22)  [n=48] | 0·68 (0·15)  [n=48] | 0·70 (0·18)  [n=48] | 0·62 (0·20)  [n=48] |
| **Placebo**  Mean (SD) [no. of obs] | 0·51 (0·23)  [n=55] | 0·56 (0·23)  [n=55] | 0·55 (0·22)  [n=55] | 0·58 (0·2)  [n=55] | 0·55 (0·18)  [n=55] |
| **Difference**  Mean difference [95%CI] | -0·01  [-0·10 to 0·08] | 0·10*  [0·01 to 0·19] | 0·12**  [0·05 to 0·20] | 0·12**  [0·05 to 0·20] | 0·07  [-0·01 to 0·14] |
| **EQ-5D VAS** |  |  |  |  |  |
| **Pramipexole**  Mean (SD) [no. of obs] | 52·77 (18·91)  [n=48] | 62·96 (17·56)  [n=48] | 62·83 (15·91)  [n=48] | 65·21 (13·87)  [n=48] | 64·28 (10·79)  [n=48] |
| **Placebo**  Mean (SD) [no. of obs] | 57·55 (17·59)  [n=55] | 57·58 (18·87)  [n=55] | 54·8 (16·66)  [n=55] | 62·17 (13·63)  [n=55] | 58·61 (14·98)  [n=55] |
| **Difference**  Mean difference [95%CI] | -4·77  [-11·96 to 2·42] | 5·37  [-1·83 to 12·5] | 8·03*  [1·58 to14·47] | 3·04  [-2·38 to 8·46] | 5·67*  [0·46 to 10·88] |
| **ICECAP-A index** |  |  |  |  |  |
| **Pramipexole**  Mean (SD) [no. of obs] | 0·48 (0·15)  [n=48] | 0·64 (0·21)  [n=48] | 0·65 (0·19)  [n=48] | 0·68 (0·16)  [n=48] | 0·66 (0·15)  [n=48] |
| **Placebo**  Mean (SD) [no. of obs] | 0·48 (0·15)  [n=55] | 0·53 (0·21)  [n=55] | 0·57 (0·17)  [n=55] | 0·62 (0·14)  [n=55] | 0·58 (0·17)  [n=55] |
| **Difference**  Mean difference [95%CI] | 0·00  [-0·06 to 0·05] | 0·11*  [0·02 to 0·19] | 0·08*  [0·01 to 0·15] | 0·06*  [0·00 to 0·12] | 0·07*  [0·02 to 0·14] |
| **OxCAP-MH score** |  |  |  |  |  |
| **Pramipexole**  Mean (SD) [no. of obs] | 57·88 (11·45)  [n=48] | 68·03 (12·82)  [n=48] | 66·64 (11·66)  [n=48] | 69·07 (9·21)  [n=48] | 66·35 (10·73)  [n=48] |
| **Placebo**  Mean (SD) [no. of obs] | 57·93 (10·08)  [n=55] | 62·16 (11·95)  [n=55] | 62·46 (10·95)  [n=55] | 63·68 (9·08)  [n=55] | 63·34 (10·52)  [n=55] |
| **Difference**  Mean difference [95%CI] | -0·04  [-4·28 to 4·19] | 5·87*  [·99 to 10·75] | 4·18  [-·27 to 8·63] | 5·38**  [1·77 to 8·99] | 3·01  [-1·17 to 7·21] |
| **Complete cases sample (n=56)** | | | | | |
| **EQ-5D-5L utility index** |  |  |  |  |  |
| **Pramipexole**  Mean (SD) [no. of obs] | 0·49 (0·21)  [n=29] | 0·63 (0·22)  [n=29] | 0·64 (0·2)  [n=29] | 0·65 (0·24)  [n=29] | 0·62 (0·23)  [n=29] |
| **Placebo**  Mean (SD) [no. of obs] | 0·49 (0·25)  [n=27] | 0·58 (0·24)  [n=27] | 0·62 (0·23)  [n=27] | 0·60 (0·27)  [n=27] | 0·59 (0·24)  [n=27] |
| **Difference**  Mean difference [95%CI] | 0·00  [-0·12 to 0·12] | 0·05  [-0·07 to 0·18] | 0·02  [-0·09 to 0·13] | 0·05  [-0·09 to 0·19] | 0·03  [-0·09 to 0·16] |
| **EQ-5D VAS** |  |  |  |  |  |
| **Pramipexole**  Mean (SD) [no. of obs] | 51·14 (20·28)  [n=29] | 58·66 (18·87)  [n=29] | 62·46 (18·87)  [n=29] | 61·28 (19·51)  [n=29] | 63·14 (15·6)  [n=29] |
| **Placebo**  Mean (SD) [no. of obs] | 56·85 (13·84)  [n=27] | 59·19 (16·86)  [n=27] | 58·22 (17·93)  [n=27] | 63·85 (19·64)  [n=27] | 62·78 (18·43)  [n=27] |
| **Difference**  Mean difference [95%CI] | -5·71  [-15·08 to 3·65] | -0·53  [-10·14 to 9·08] | 4·24  [-5·72 to 14·20] | -2·57  [-13·06 to 7·91] | 0·36  [-8·76 to 9·48] |
| **ICECAP-A index** |  |  |  |  |  |
| **Pramipexole**  Mean (SD) [no. of obs] | 0·48 (0·16)  [n=29] | 0·62 (0·2)  [n=29] | 0·62 (0·24)  [n=29] | 0·62 (0·22)  [n=29] | 0·64 (0·2)  [n=29] |
| **Placebo**  Mean (SD) [no. of obs] | 0·49 (0·15)  [n=27] | 0·60 (0·2)  [n=27] | 0·60 (0·18)  [n=27] | 0·60 (0·21)  [n=27] | 0·61 (0·23)  [n=27] |
| **Difference**  Mean difference [95%CI] | -0·01  [-0·09 to 0·07] | 0·02  [-0·08 to 0·13] | 0·02  [-0·10 to 0·12] | ·02  [-0·09 to 0·13] | 0·03  [-0·07 to 0·15] |
| **OxCAP-MH score** |  |  |  |  |  |
| **Pramipexole**  Mean (SD) [no. of obs] | 57·22 (11·39)  [n=29] | 67·56 (13·25)  [n=29] | 66·22 (14·71)  [n=29] | 67·19 (14·17)  [n=29] | 66·54 (13·68)  [n=29] |
| **Placebo**  Mean (SD) [no. of obs] | 56·19 (11·13)  [n=27] | 62·15 (11·71)  [n=27] | 63·02 (13·39)  [n=27] | 62·21 (12·55)  [n=27] | 63·43 (14·08)  [n=27] |
| **Difference**  Mean difference [95%CI] | 1·02  [-5·01 to 7·06] | 5·41  [-1·30 to 12·13] | 3·19  [-4·35 to 10·75] | 4·97  [-2·21 to 12·16] | 3·11  [-4·32 to 10·55] |

Note: PP - per protocol; CI - confidence interval; SD - standard deviation; EQ-5D-5L - European Quality of Life 5 Dimensions 5 Level; ICECAP-A - ICEpop CAPability measure for Adults; OxCAP-MH - Oxford Capability Questionnaire – Mental Health; *p<0·05, **p<0·01, ***p<0·001

Appendix Table 10. Sensitivity analyses: Mean incremental costs, QALYs, YFC, CWLYs and ICERs (pramipexole vs. placebo)

|  | ΔC [95% CI] | ΔE [95% CI] | ICER† [95% CI] | Probability of CE at £20,000/£30,000 WTP |  |
| --- | --- | --- | --- | --- | --- |
| **Sensitivity analysis 1: PP** | | | | | |
| **NHS+PSS perspective** over 48 weeks | |  |  |  |  |
| QALY *(*EQ-5D-5L) | £834  [-£35 to £1,702] | 0·092  [0·033 to 0·151] | £9,065/QALY  [£1,390 to £39,038] | 88%/96% |  |
| YFC (ICECAP-A) | £834  [-£35 to £1,702] | 0·068  [0·020 to 0·117] | £12,205/YFC  [£2,118 to £47,588] | 80%/91% |  |
| CWLY (OxCAP-MH) | £834  [-£35 to £1,702] | 0·041  [0·013 to 0·069] | £20,333/CWLY  [£2,700 to £63,584] | 51%/76% |  |
| **Societal perspective** over 48 weeks | |  |  |  |  |
| QALY *(*EQ-5D-5L) | -£859  [-£2,328 to £611] | 0·092  [0·033 to 0·151] | -£9,336/QALY  [-£32,440 to £10,126] | 99%/99·8% |  |
| YFC (ICECAP-A) | -£859  [-£2,328 to £611] | 0·068  [0·020 to 0·117] | -£12,569/YFC  [-£44,259 to £13,857] | 99%/99·4% |  |
| CWLY (OxCAP-MH) | -£859  [-£2,328 to £611] | 0·041  [0·013 to 0·069] | -£20,939/CWLY  [-£105,158 to £20,812] | 97%/99% |  |
| **Sensitivity analysis 2: Complete cases** | | | | | |
| **NHS+PSS perspective** over 48 weeks | |  |  |  |  |
| QALY *(*EQ-5D-5L) | £796  [-£677 to £2,269] | 0·038  [-0·050 to 0·126] | £20,840/QALY  [-£166,588 to £207,354] | 54%/62% |  |
| YFC (ICECAP-A) | £796  [-£677 to £2,269] | 0·030  [-0·044 to 0·104] | £26,484/YFC  [-£233,539 to £295,605] | 51%/63% |  |
| CWLY (OxCAP-MH) | £796  [-£677 to £2,269] | 0·029  [-0·012 to 0·070] | £27,586/CWLY  [-£37,321 to £287,288] | 44%/59% |  |
| **Societal perspective** over 48 weeks | |  |  |  |  |
| QALY *(*EQ-5D-5L) | -£681  [-£3,619 to £2,256] | 0·038  [-0·050 to 0·126] | -£17,827/QALY  [-£235,157 to £254,673] | 83%/84% |  |
| YFC (ICECAP-A) | -£681  [-£3,619 to £2,256] | 0·030  [-0·044 to 0·104] | -£22,654/YFC  [-£241,588 to £216,286] | 82%/83% |  |
| CWLY (OxCAP-MH) | -£681  [-£3,619 to £2,256] | 0·029  [-0·012 to 0·070] | -£23,597/CWLY  [-£369,712 to £161,828] | 86%/90% |  |
| **Sensitivity analysis 3: Alternative costs** | | | | | |
| **NHS+PSS perspective** over 48 weeks | |  |  |  |  |
| QALY *(*EQ-5D-5L) | £871  [£165 to £1,577] | 0·090  [0·036 to 0·144] | £9,677/QALY  [£3,062 to £28,677] | 91%/98% |  |
| YFC (ICECAP-A) | £871  [£165 to £1,577] | 0·064  [0·019 to 0·109] | £13,542/YFC  [£3,749 to £45,056] | 77%/93% |  |
| CWLY (OxCAP-MH) | £871  [£165 to £1,577] | 0·038  [0·012 to 0·064] | £22,906/CWLY  [£7,205 to £68,230] | 41%/70% |  |
| **Societal perspective** over 48 weeks | |  |  |  |  |
| QALY *(*EQ-5D-5L) | -£241  [-£1,597 to £1,116] | 0·090  [0·036 to 0·144] | -£2,674/QALY  [-£20,593 to £18,134] | 98%/99% |  |
| YFC (ICECAP-A) | -£241  [-£1,597 to £1,116] | 0·064  [0·019 to 0·109] | -£3,742/YFC  [-£28,840 to £24,333] | 97%/99% |  |
| CWLY (OxCAP-MH) | -£241  [-£1,597 to £1,116] | 0·038  [0·012 to 0·064] | -£6,329/CWLY  [-£62,481 to £30,725] | 92%/97% |  |
| **Sensitivity analysis 4: Alternative outcomes** | | | | | |
| **NHS+PSS perspective** over 48 weeks | |  |  |  |  |
| QALY *(*EQ-5D-5L) | £871  [£165 to £1,577] | 0·097  [0·035 to 0·159] | £8,363/QALY  [£2,023 to £27,530] | 94%/98% |  |
| YFC (ICECAP-A) | £871  [£165 to £1,577] | 0·074  [0·024 to 0·125] | £10,910  [£2,496 to £34,057] | 89%/96% |  |
| CWLY (OxCAP-MH) | £871  [£165 to £1,577] | 0·041  [0·012 to 0·071] | £19,652  [£5,816 to £61,518] | 53%/81% |  |
| **Societal perspective** over 48 weeks | |  |  |  |  |
| QALY *(*EQ-5D-5L) | -£241  [-£1,597 to £1,116] | 0·097  [0·035 to 0·159] | -£2,629/QALY  [-£17,251 to £15,211] | 98%/99% |  |
| YFC (ICECAP-A) | -£241  [-£1,597 to £1,116] | 0·074  [0·024 to 0·125] | -£3,429  [-£25,158 to £27,858] | 97%/98% |  |
| CWLY (OxCAP-MH) | -£241  [-£1,597 to £1,116] | 0·041  [0·012 to 0·071] | -£6,177  [-£58,772 to £30,041] | 93%/97% |  |
| **Sensitivity analysis 5: Current pramipexole cost** | | | | |  |
| **NHS+PSS perspective** |  |  |  |  |  |
| QALY *(*EQ-5D-5L) over 12 weeks | £130  [£15 to £246] | 0·012  [0·003 to 0·021] | £10,933  [£1,944 to £46,784 | 82%/93% |  |
| QALY *(*EQ-5D-5L) over 48 weeks | £1,053  [£376 to £1,731] | 0·090  [0·036 to 0·144] | £11,700  [£4,388 to £35,100] | 87%/95% |  |
| **Societal perspective** |  |  |  |  |  |
| QALY *(*EQ-5D-5L) over 12 weeks | £120  [-£623 to £863 | 0·012  [0·003 to 0·021] | £10,082  [-£66,716 to £135,213] | 82%93% |  |
| QALY *(*EQ-5D-5L) over 48 weeks | -£12  [-£1,360 to £1,335] | 0·090  [0·036 to 0·144] | -£138  [-£16,078 to £23,392] | 96%/98% |  |

Note: † The ICERs presented in this table are calculated from the unrounded cost and outcome differences (including decimal points), while the values in the table are rounded for simplicity. The discrepancy between the table values and the ICERs is due to this rounding. Confidence intervals for the ICERs were calculated using bootstrap methods; PP – per protocol; QALY – quality-adjusted life year; YFC – year of full capability; CWLY – capability-weighted life year; ICER - Incremental cost-effectiveness ratio; CI - Confidence interval; NHS+PSS: National Health Service and Personal Social Services; CE – cost-effectiveness; WTP – willingness-to-pay; EQ-5D-5L - European Quality of Life 5 Dimensions 5 Level; ICECAP-A - ICEpop CAPability measure for Adults; OxCAP-MH - Oxford Capability Questionnaire – Mental Health

Appendix Table 11. Results of primary net monetary benefit (NMB) and net health benefit (NHB) analyses

| Perspective | WTP threshold  £20,000 | | WTP threshold  £30,000 | |
| --- | --- | --- | --- | --- |
|  | 12 weeks | 48 weeks | 12 weeks | 48 weeks |
|  | **NMB (95% CI)** | **NMB (95% CI)** | **NMB (95% CI)** | **NMB (95% CI)** |
| NHS+PSS | £177  (-£68 to £401) | £989  (-£360 to £2,210) | £296  (-£32 to £597) | £1,889  (£101 to £3,566) |
| Societal | £187  (-£573 to £1,006) | £2,055  (£305 to £3,869) | £306  (-£494 to £1,151) | £2,955  (£804 to £5,224) |
|  | **NHB (95% CI)** | **NHB (95% CI)** | **NHB (95% CI)** | **NHB (95% CI)** |
| NHS+PSS | 0·009 QALY (-0·003 to 0·020) | 0·050 QALY (-0·018 to 0·111) | 0·010 QALY (-0·001 to 0·020) | 0·063 QALY (0·003 to 0·119) |
| Societal | 0·009 QALY (-0·029 to 0·050) | 0·103 QALY (0·015 to 0·193) | 0·01 QALY (-0·016 to 0·038) | 0·099 QALY (0·027 to 0·174) |

Note: WTP - Willingness-to-pay threshold, NHS+PSS - National Health Service and Personal Social Services, CI - confidence interval, NHB – net health benefit, NMB – net monetary benefit, QALY – quality-adjusted life year

## Figures

Appendix Figure 1 Health economic outcome measure results over time (n=124)

Note: The following health economic outcomes are visible on the graph: EQ-5D-5L (NW), EQ-5D Visual Analog Scale (NE), OxCAP-MH (SW), ICECAP-A (SE); lb/up – lower/upper bound of the 95% confidence interval

Appendix Figure 2. Sensitivity analysis: health economic outcomes for PP sample (n=103)

Note: The following health economic outcomes are visible on the graph: EQ-5D-5L (NW), EQ-5D Visual Analog Scale (NE), OxCAP-MH (SW), ICECAP-A (SE); lb/up – lower/upper bound of the 95% confidence interval

Appendix Figure 3. Sensitivity analysis: health economic outcomes for complete cases sample (n=56)

Note: The following health economic outcomes are visible on the graph: EQ-5D-5L (NW), EQ-5D Visual Analog Scale (NE), OxCAP-MH (SW), ICECAP-A (SE); lb/up – lower/upper bound of the 95% confidence interval

| 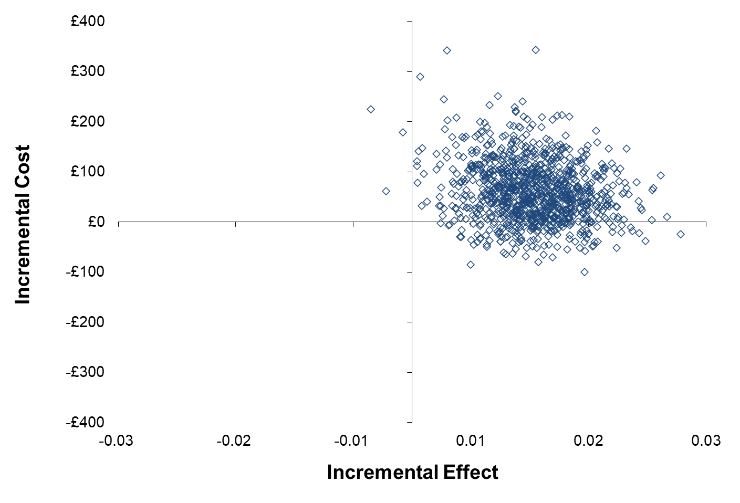  a | 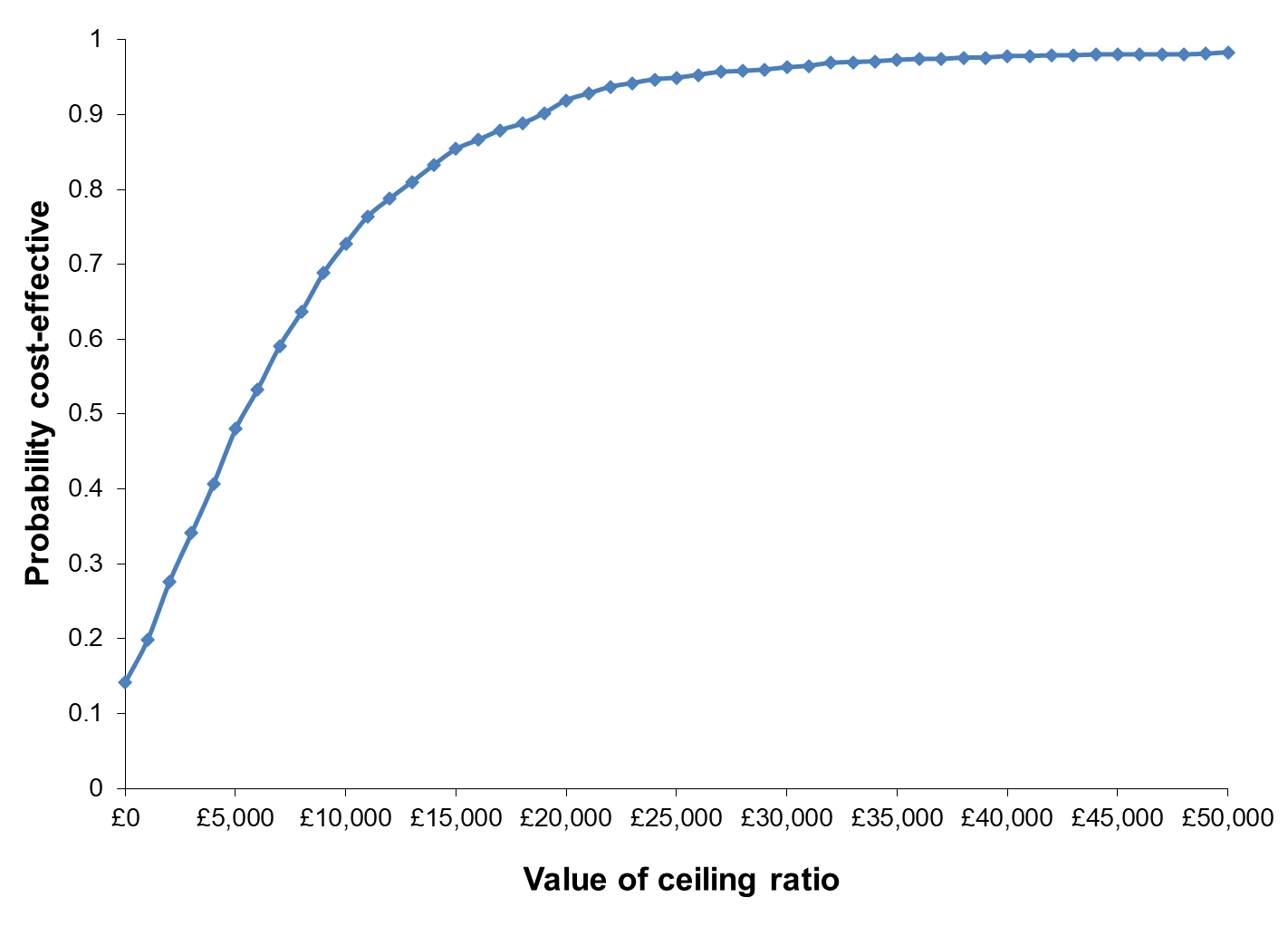 |
| --- | --- |
| 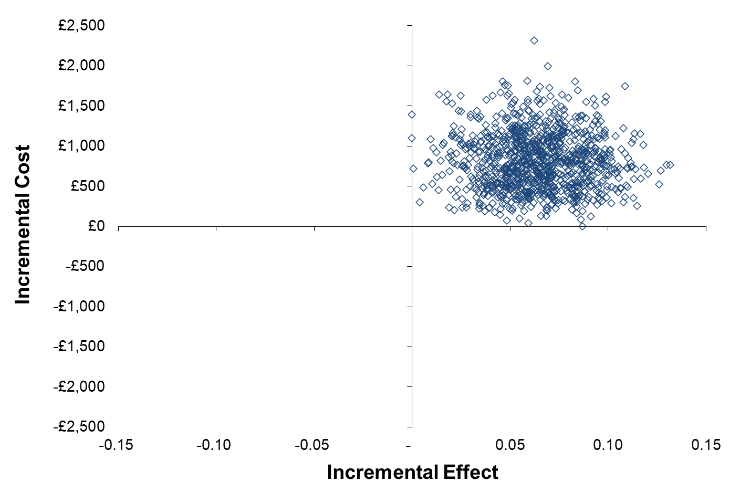  b | 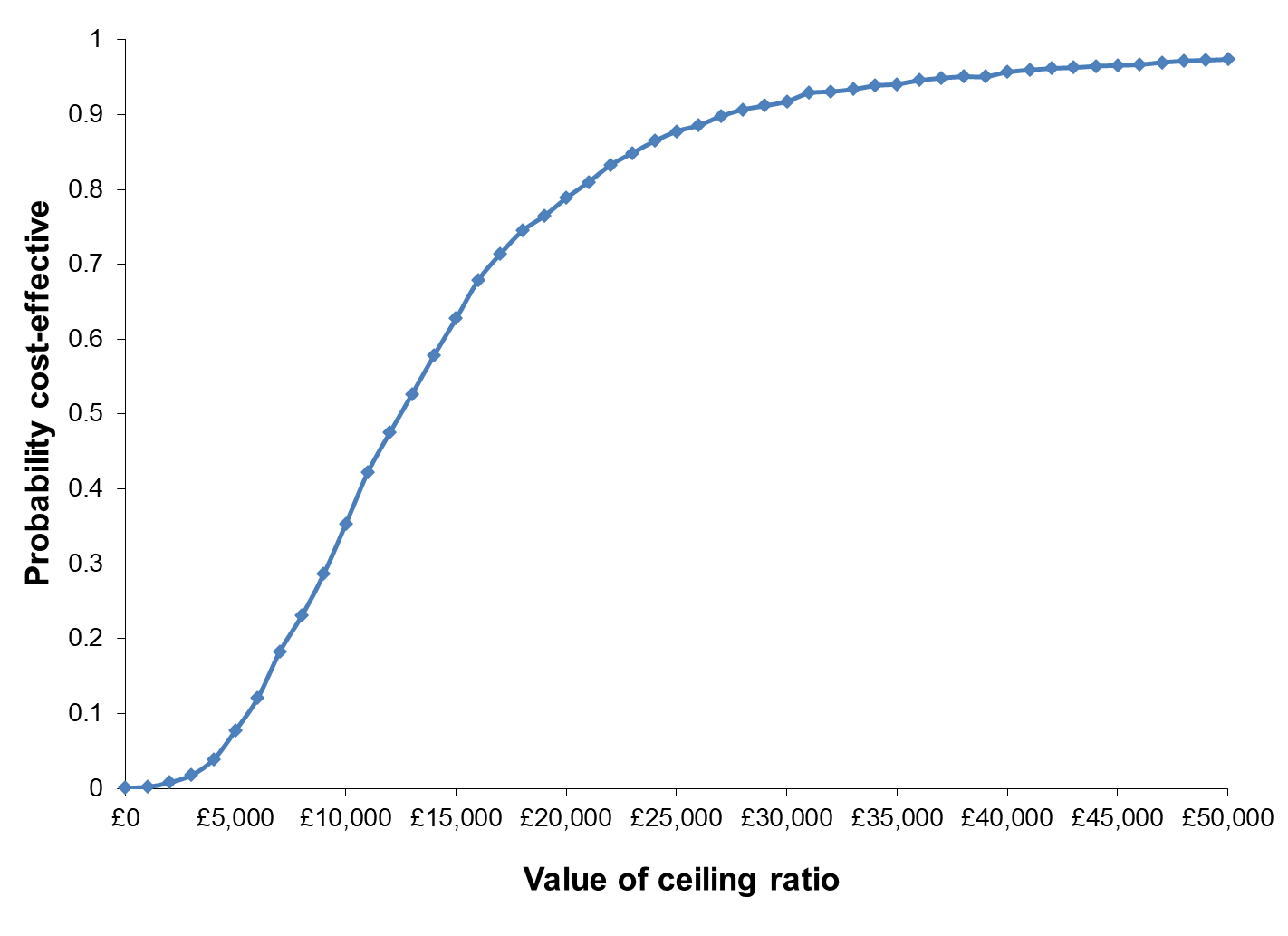 |

Appendix Figure 4. Secondary cost-effectiveness analysis for YFC (NHS+PSS perspective)

Note: Left: Cost-effectiveness plane with bootstrapped ICERs for pramipexole against placebo treatment presenting cost from the NHS+PSS perspective per year of full capability (YFC) gained over a) 12 weeks, and b) 48 weeks;

Right: Cost-Effectiveness Acceptability Curve (CEAC) showing the probability of pramipexole being cost-effective in comparison to placebo treatment at different willingness-to-pay thresholds for YFC gained.

a

| 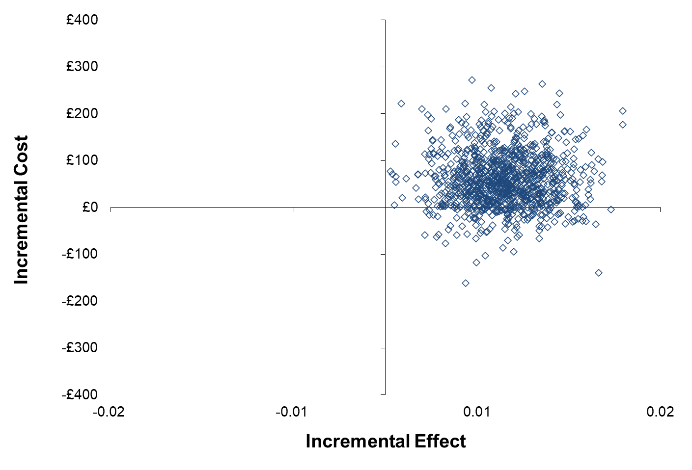  b | 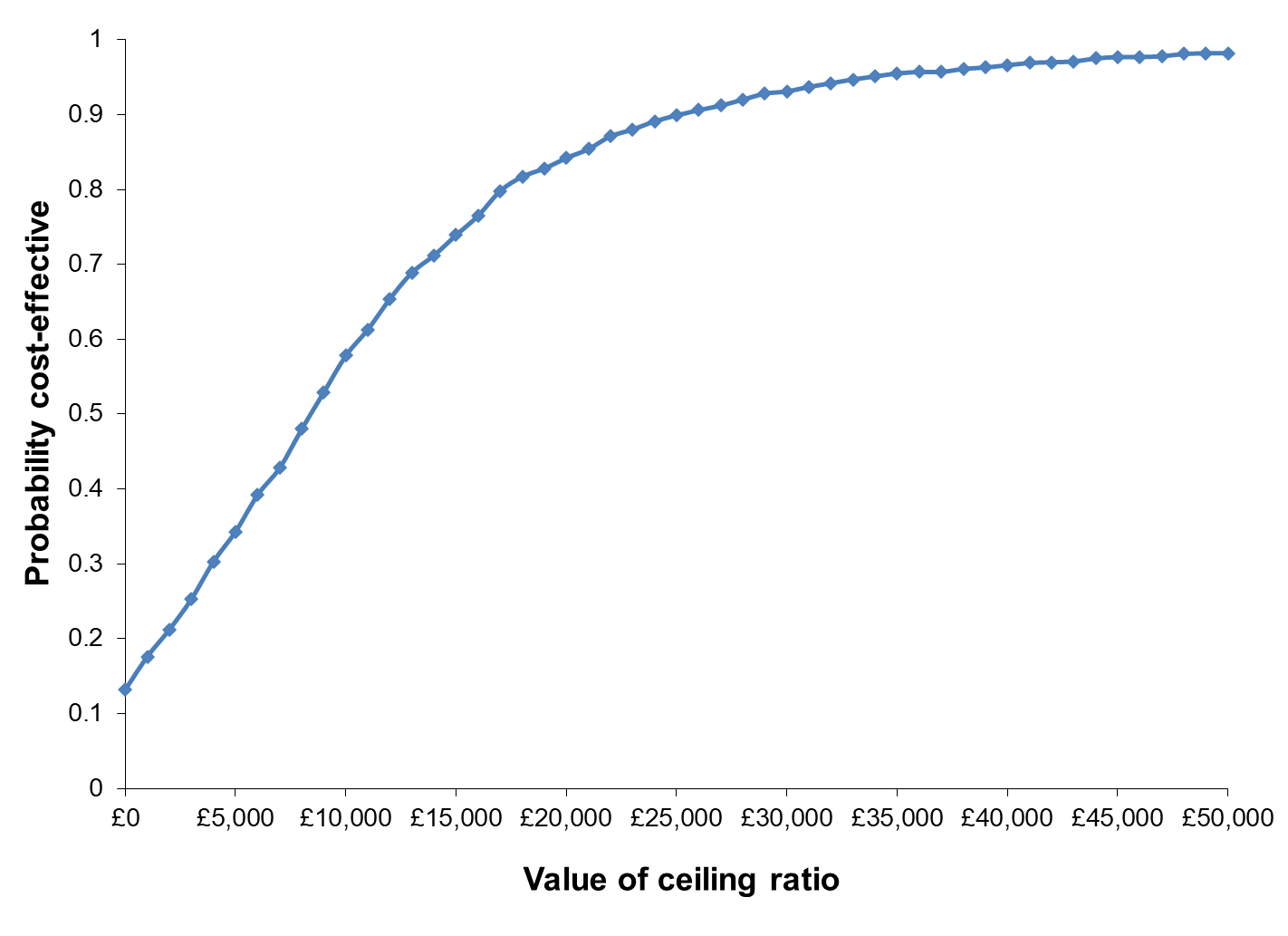 |
| --- | --- |
| 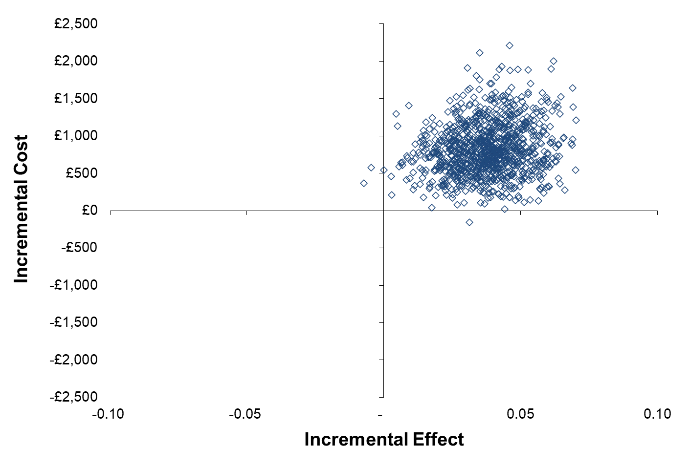 | 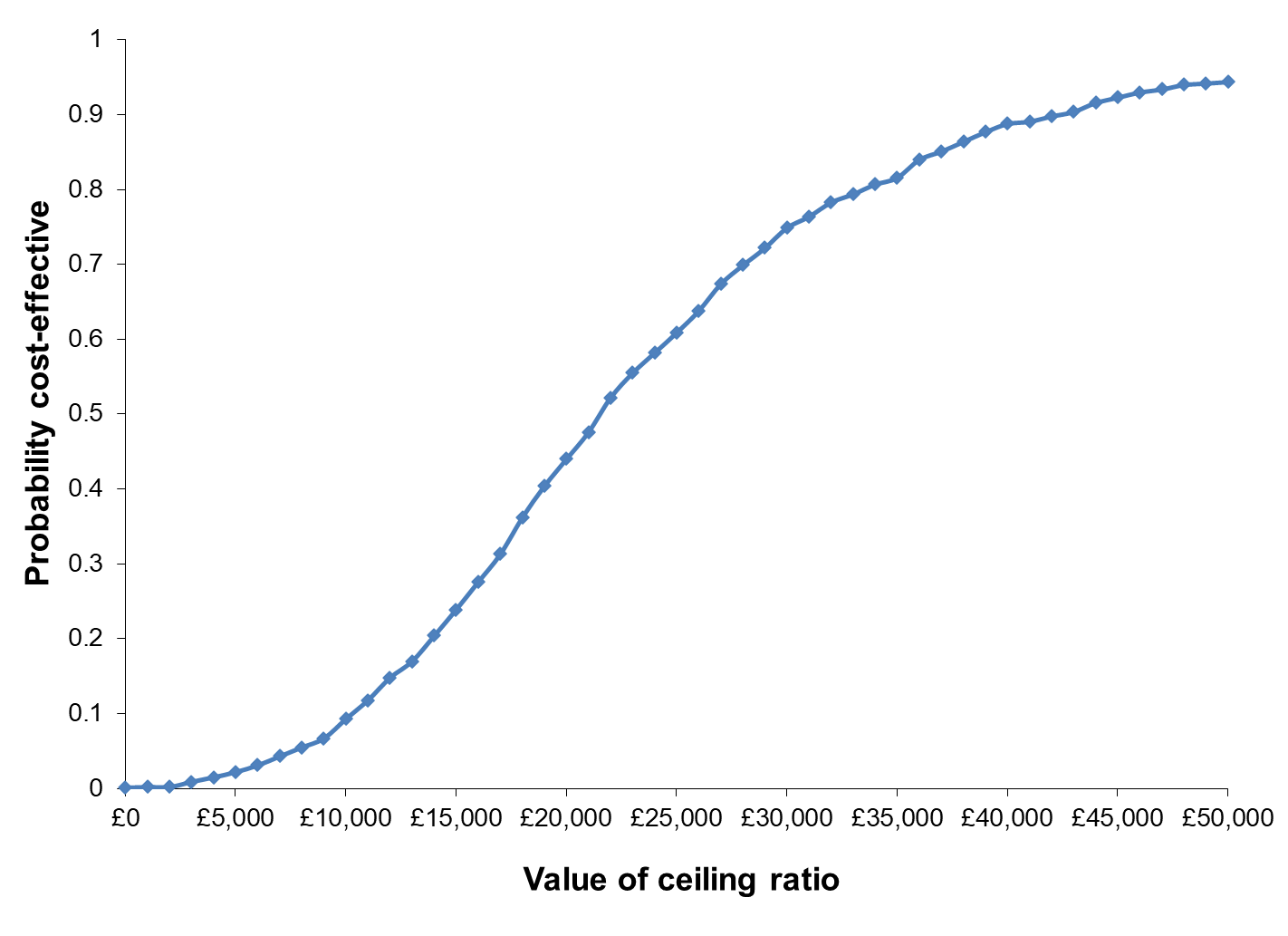 |

Appendix Figure 5. Secondary cost-effectiveness analysis for CWLY (NHS+PSS perspective)

Note: Left: Cost-effectiveness plane with bootstrapped ICERs for pramipexole against placebo treatment presenting cost from the NHS+PSS perspective per capability-weighted life year (CWLY) gained over a) 12 weeks, and b) 48 weeks;

Right: Cost-Effectiveness Acceptability Curve (CEAC) showing the probability of pramipexole being cost-effective in comparison to placebo treatment at different willingness-to-pay thresholds for CWLY gained.

| 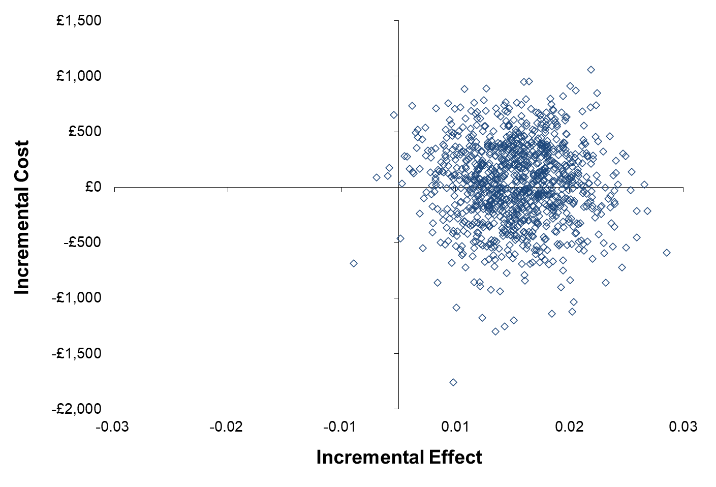 | 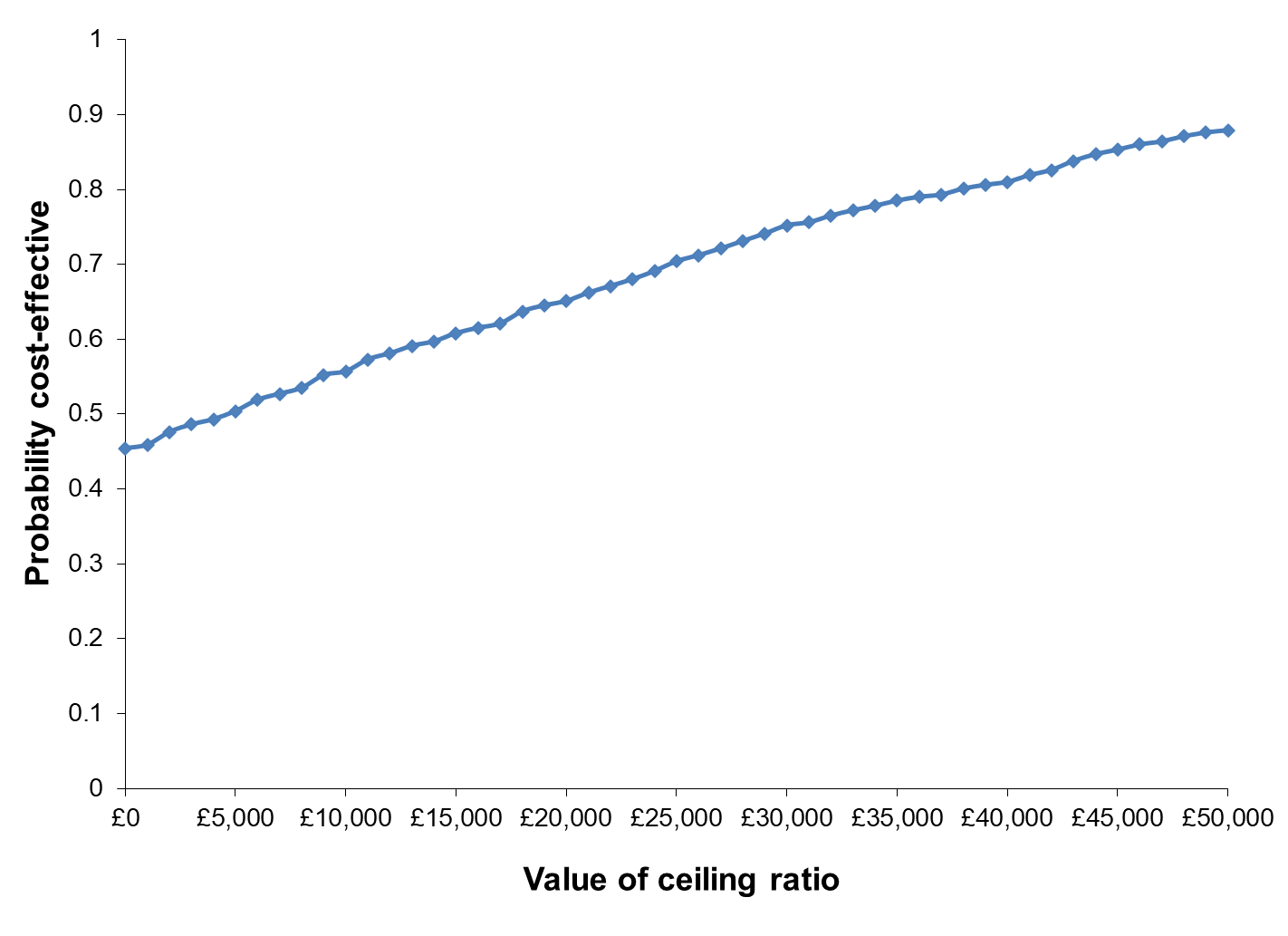 |
| --- | --- |
| 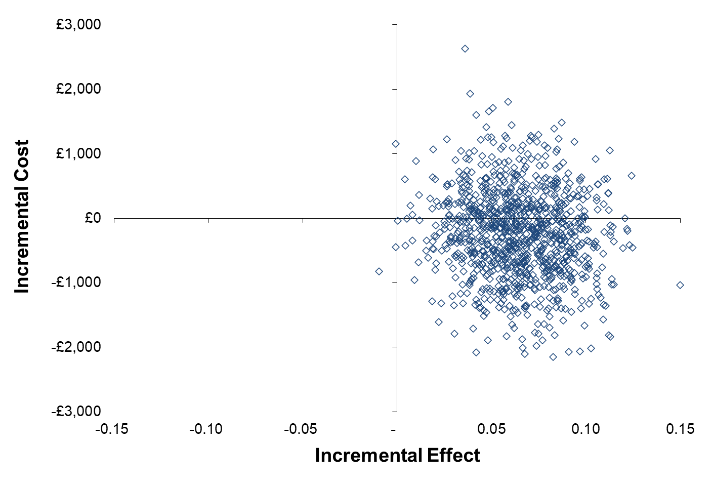 | 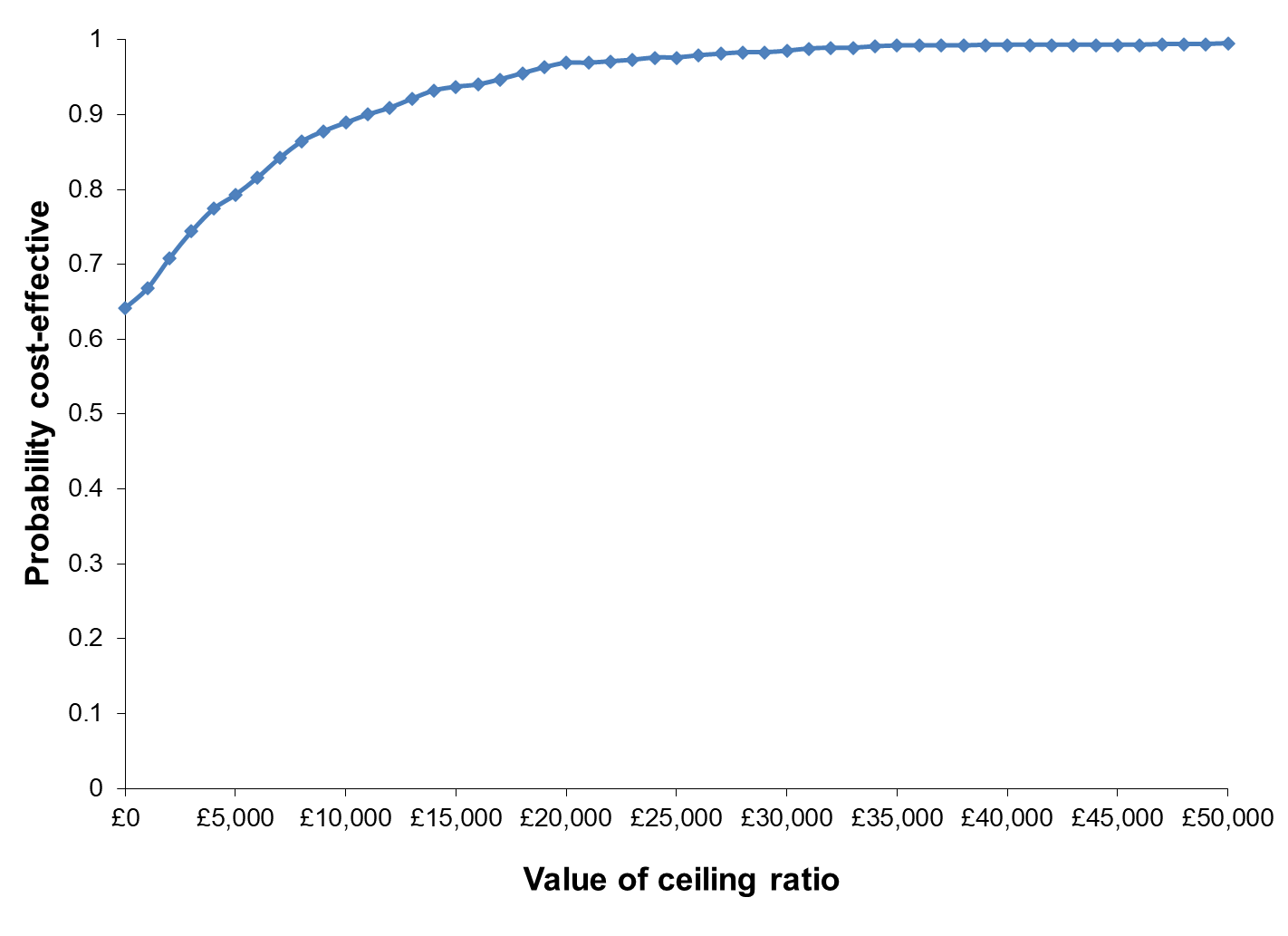 |

Appendix Figure 6. Secondary cost-effectiveness analysis for YFC (societal perspective)

a

b

Note: Left: Cost-effectiveness plane with bootstrapped ICERs for pramipexole against placebo treatment presenting cost from the societal perspective per year of full capability (YFC) gained over a) 12 weeks, and b) 48 weeks;

Right: Cost-Effectiveness Acceptability Curve (CEAC) showing the probability of pramipexole being cost-effective in comparison to placebo treatment at different willingness-to-pay thresholds for YFC gained.

| 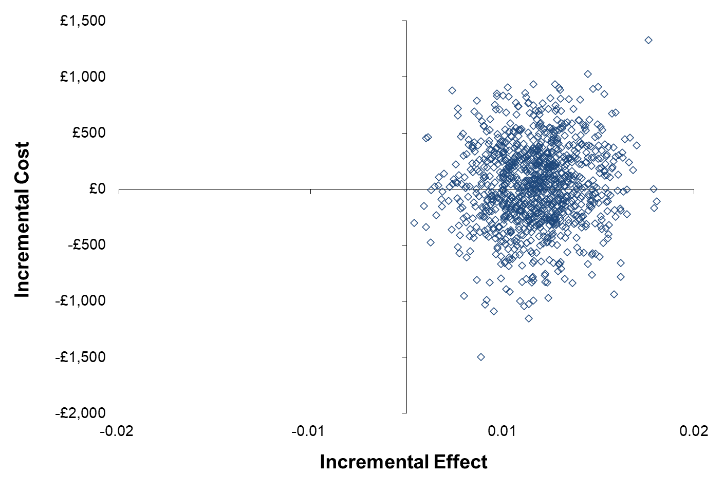  b | 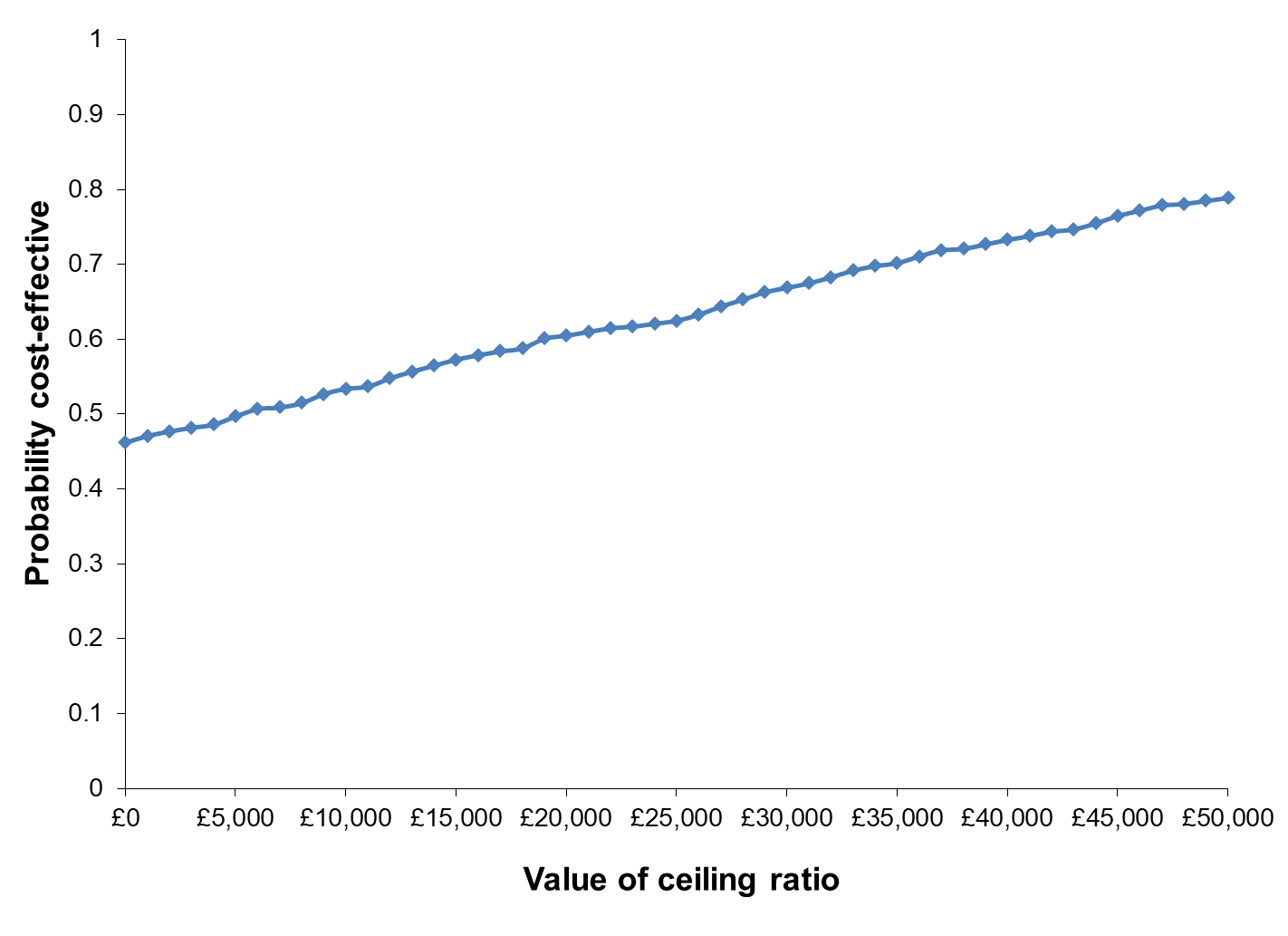 |
| --- | --- |
| 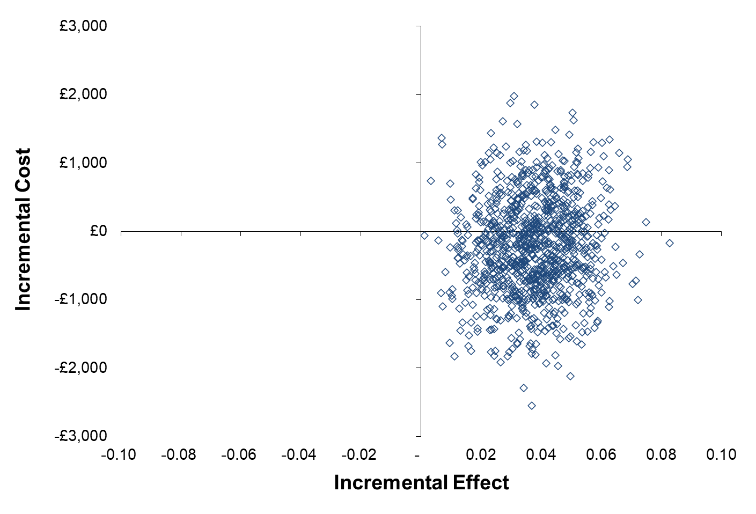 | 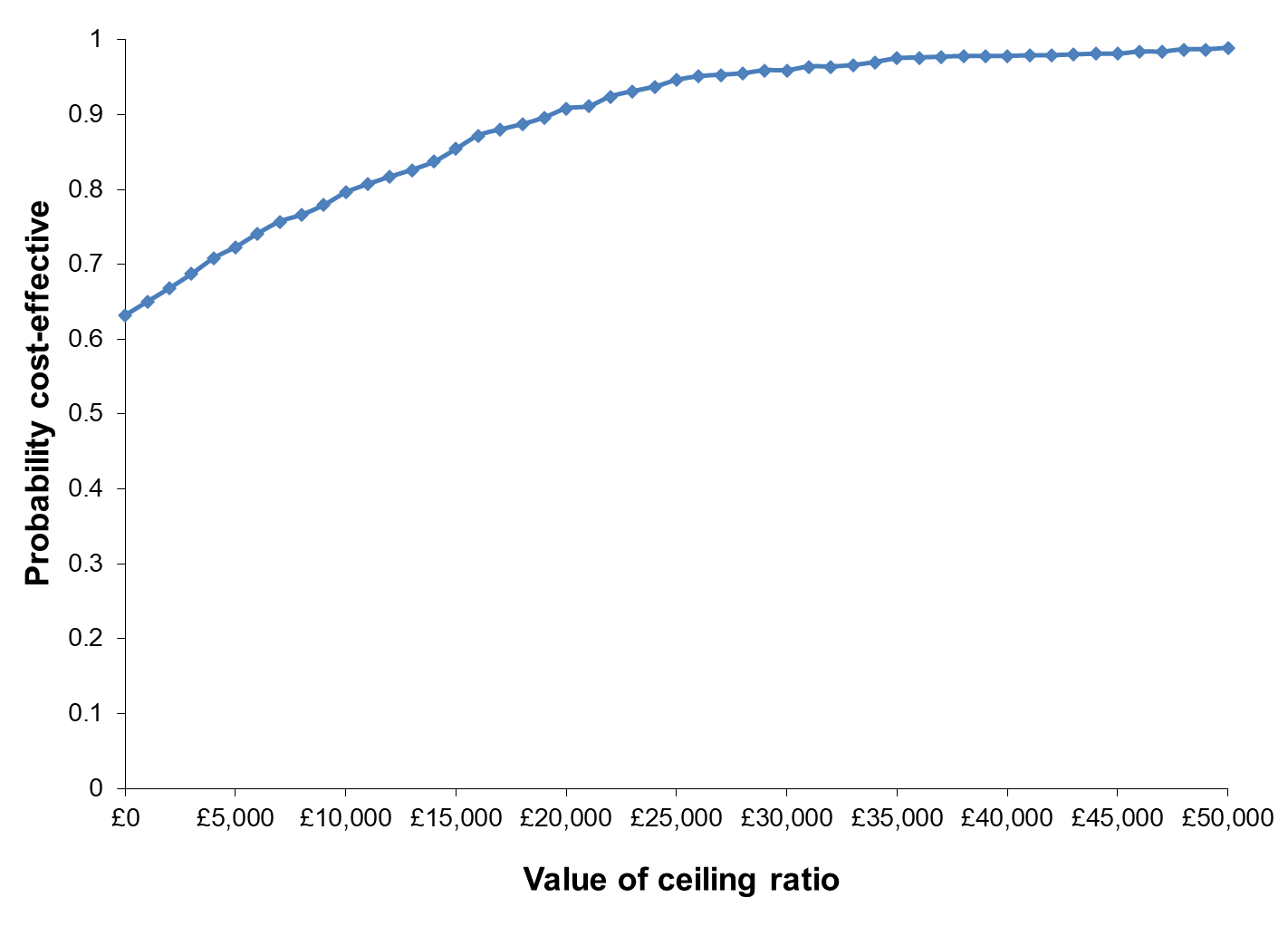 |

Appendix Figure 7. Secondary cost-effectiveness analysis for CWLY (societal perspective)

a

Note: Left: Cost-effectiveness plane with bootstrapped ICERs for pramipexole against placebo treatment

presenting cost from the societal perspective per capability-weighted life year (CWLY) gained over a) 12 weeks, and b) 48 weeks;

Right: Cost-Effectiveness Acceptability Curve (CEAC) showing the probability of pramipexole being cost-effective in comparison to placebo treatment at different willingness-to-pay thresholds for CWLY gained.

| 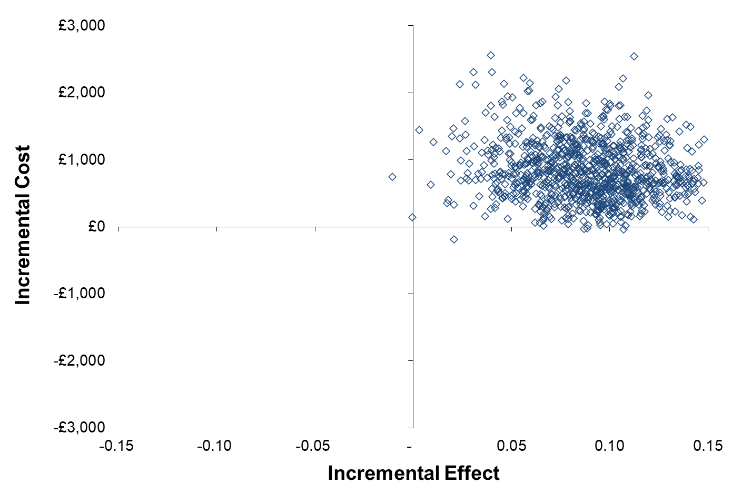  b | 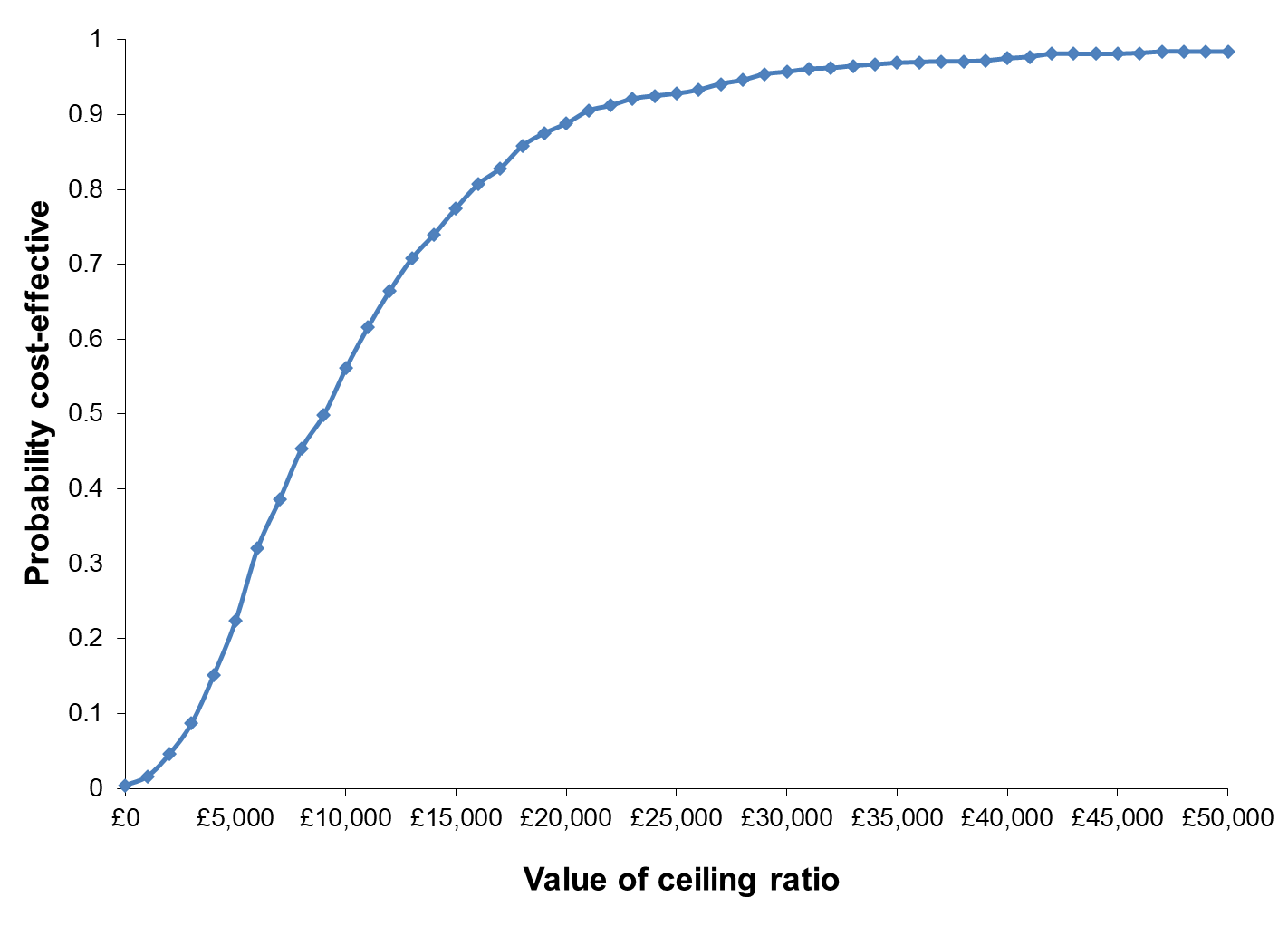 |
| --- | --- |
| 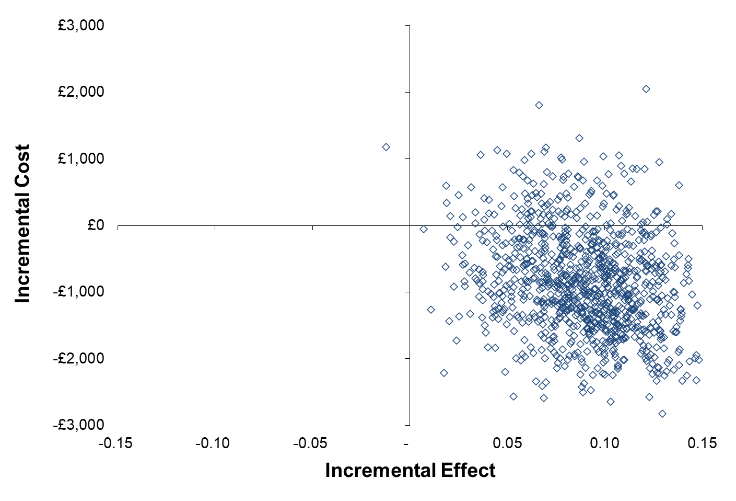 | 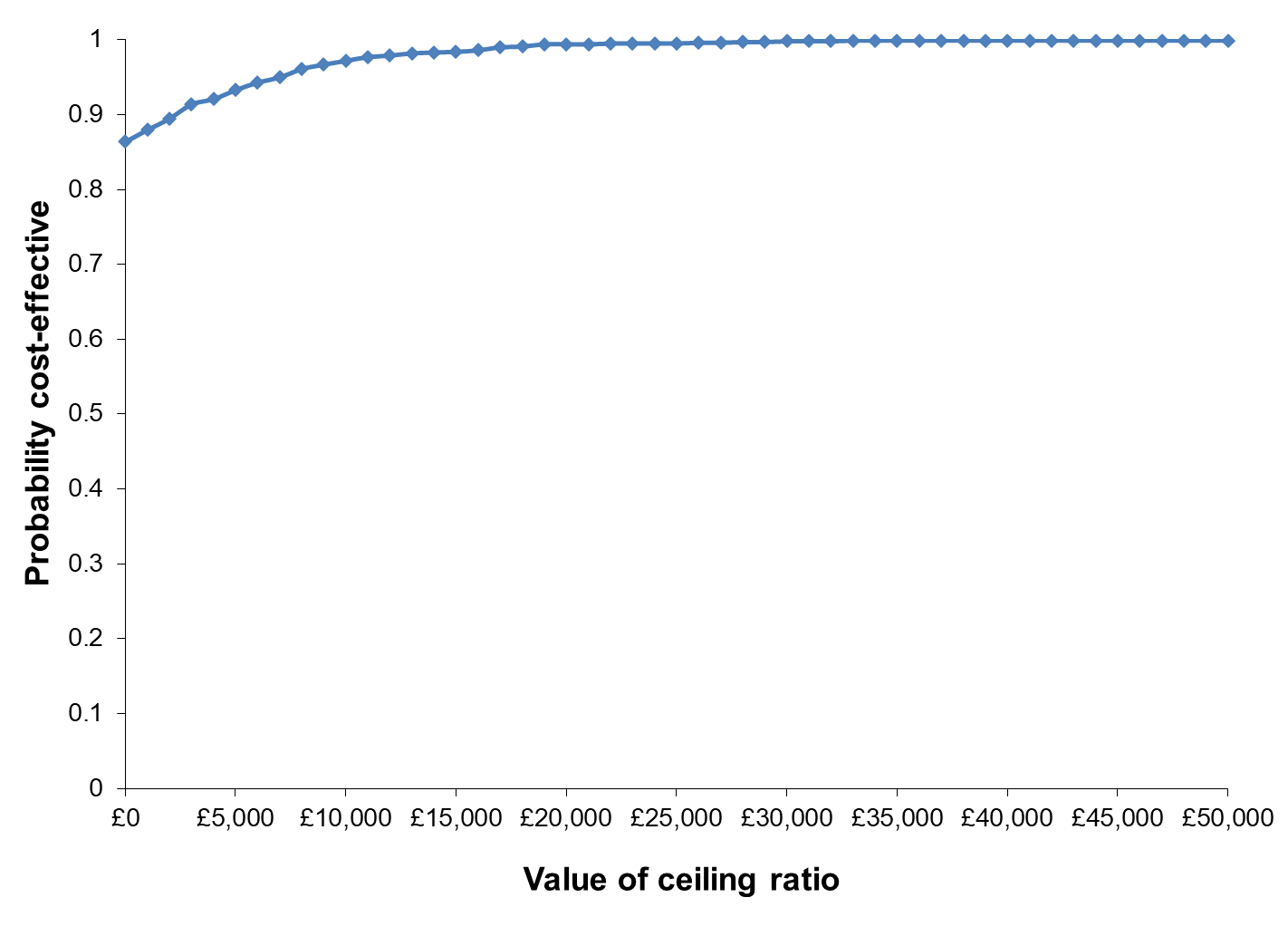 |

Appendix Figure 8. Sensitivity analysis: Cost-effectiveness analysis for QALY (per protocol)

a

Note: Left: Cost-effectiveness plane with bootstrapped ICERs for pramipexole against placebo treatment presenting results for the per protocol sample for quality-adjusted life year (QALY) gained over 48 weeks from the a) NHS+PSS perspective, and b) societal perspective;

Right: Cost-Effectiveness Acceptability Curve (CEAC) showing the probability of pramipexole being cost-effective in comparison to placebo treatment at different willingness-to-pay thresholds for QALY gained.

| 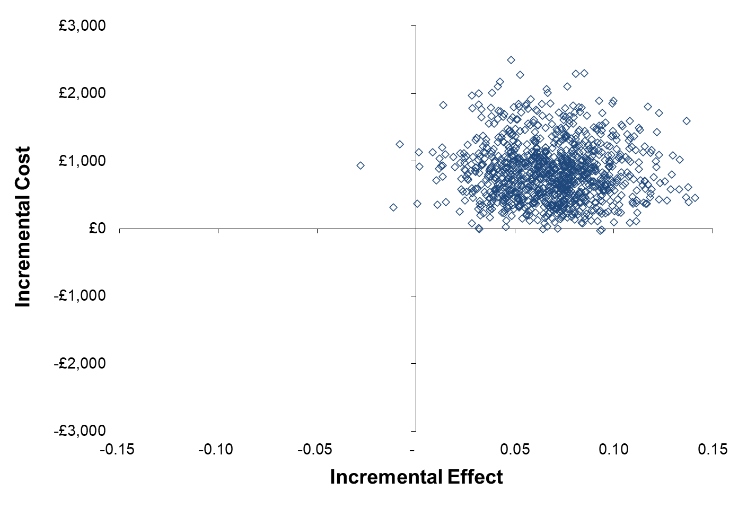  b | 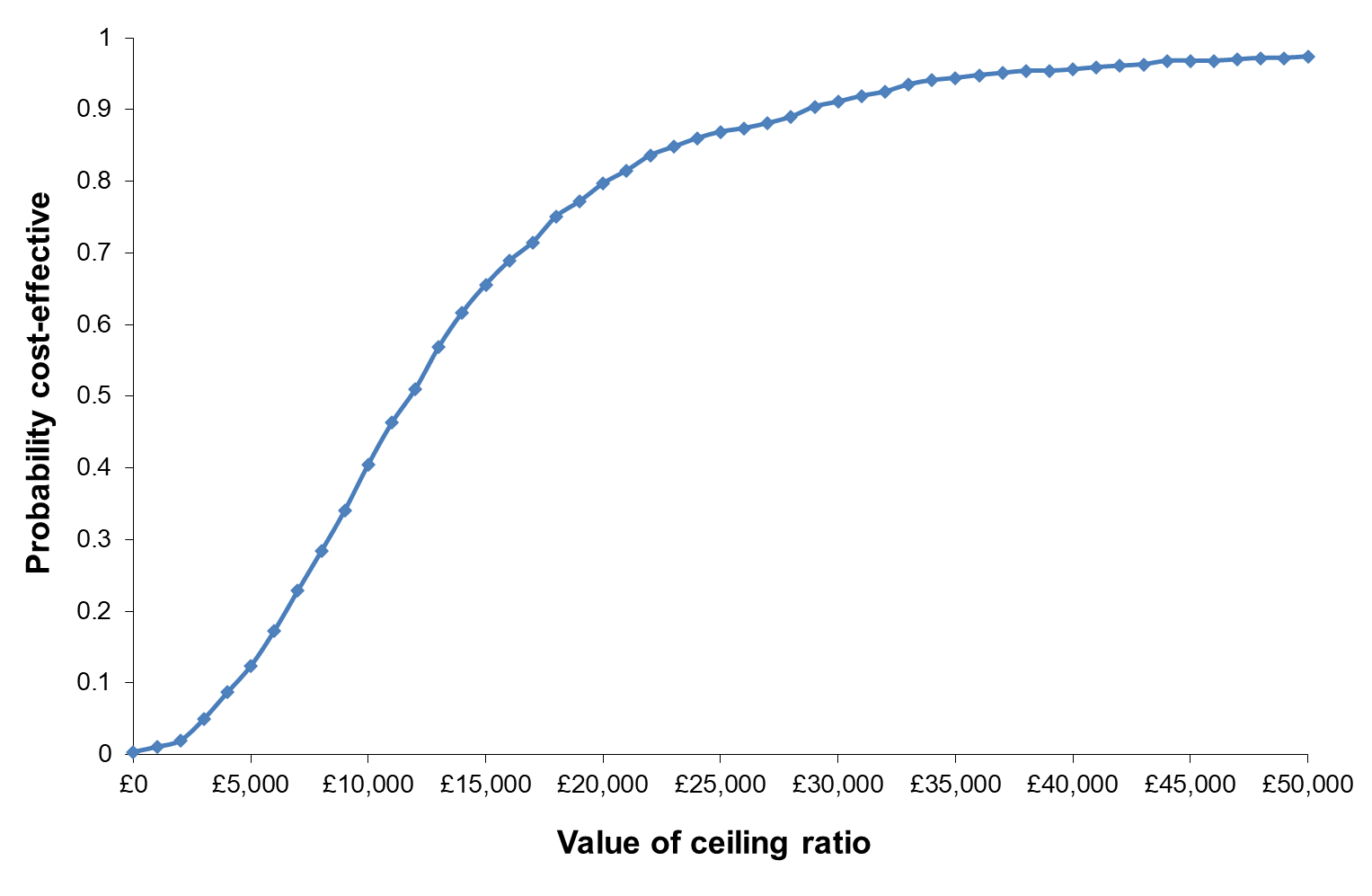 |
| --- | --- |
| 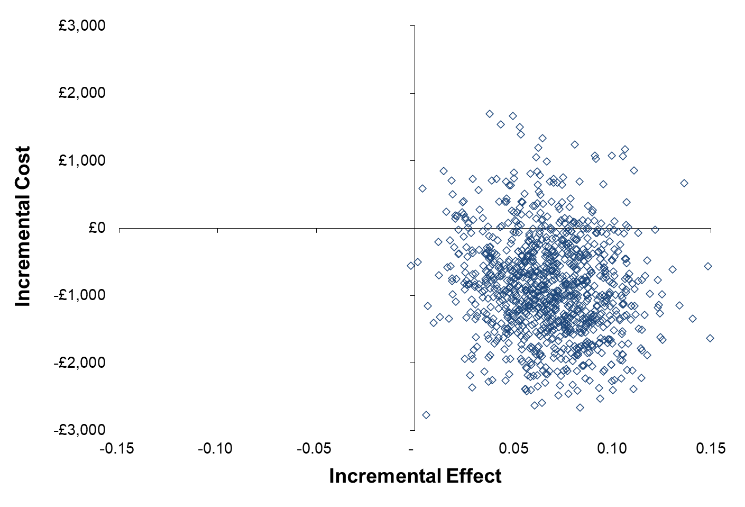 | 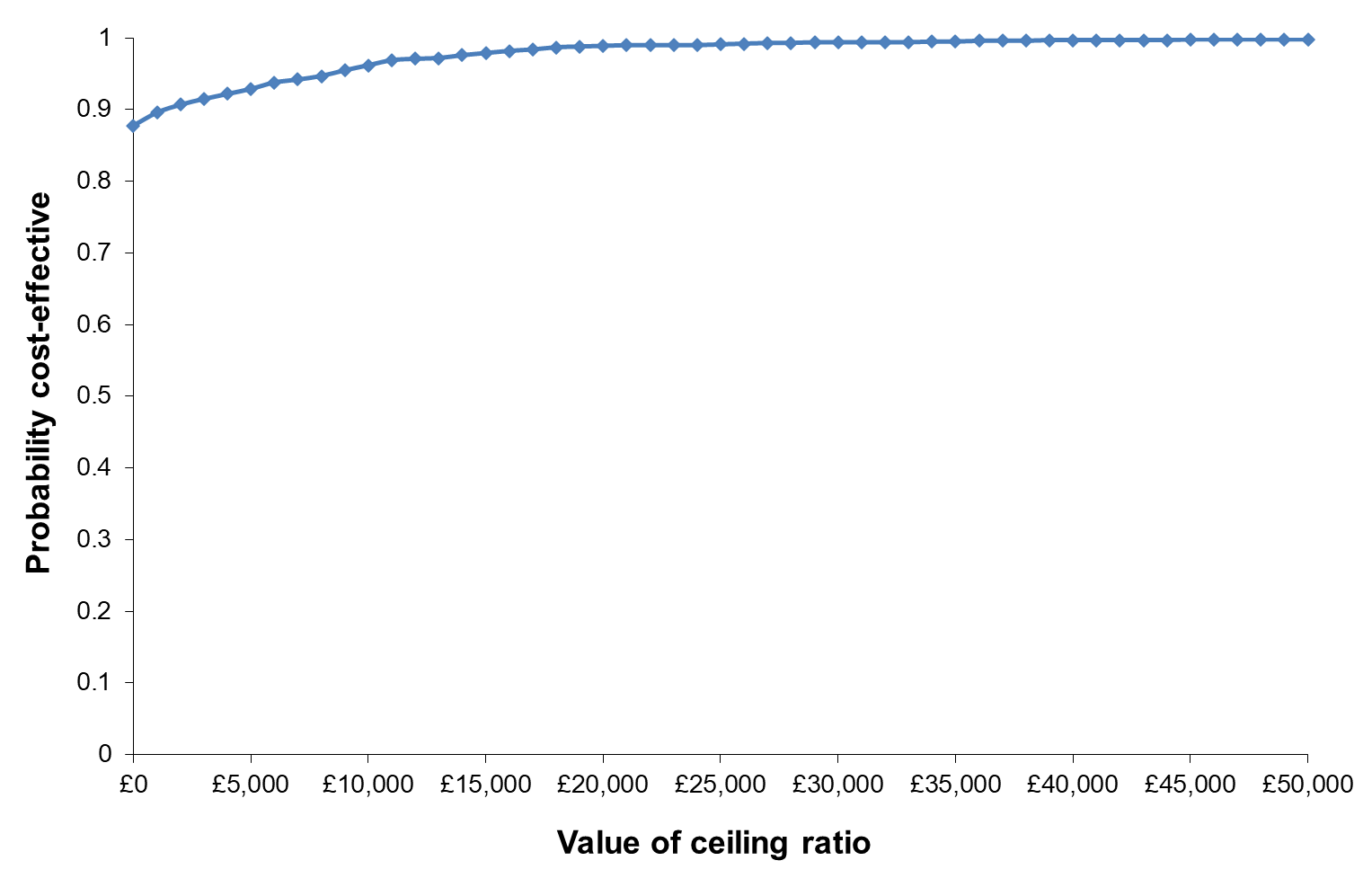 |

Appendix Figure 9. Sensitivity analysis: Cost-effectiveness for YFC (per protocol)

a

Note: Left: Cost-effectiveness plane with bootstrapped ICERs for pramipexole against placebo treatment presenting results for the per protocol sample for year of full capability (YFC) gained over 48 weeks from the a) NHS+PSS perspective, and b) societal perspective;

Right: Cost-Effectiveness Acceptability Curve (CEAC) showing the probability of pramipexole being cost-effective in comparison to placebo treatment at different willingness-to-pay thresholds for YFC gained.

| 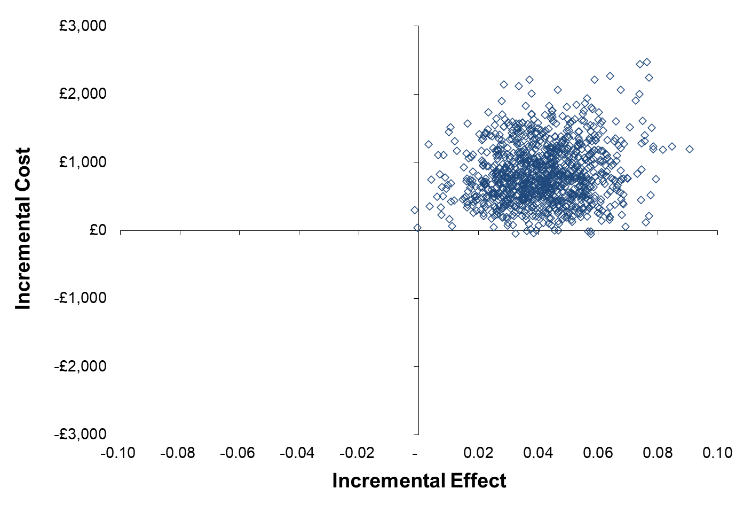  b | 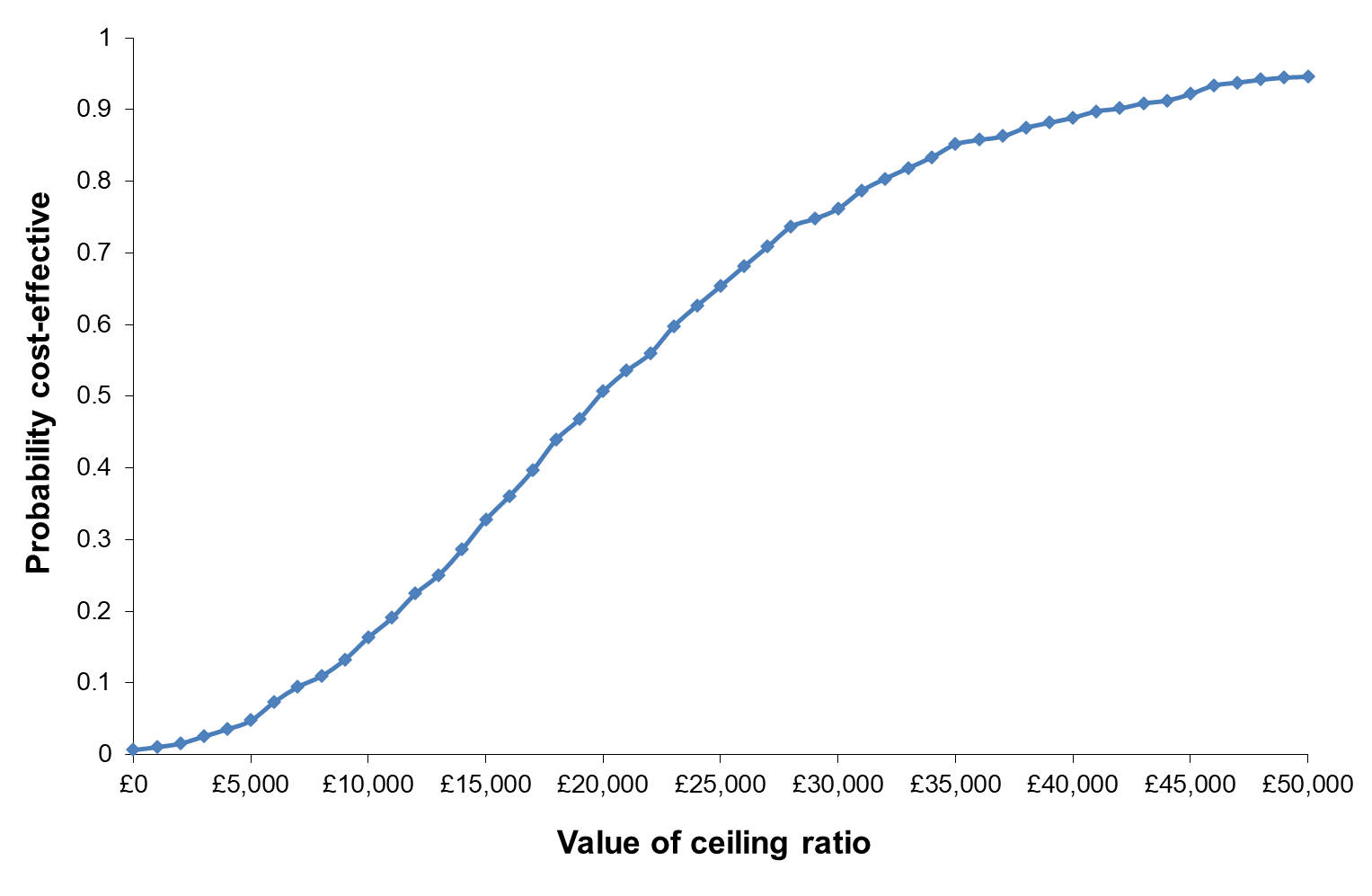 |
| --- | --- |
| 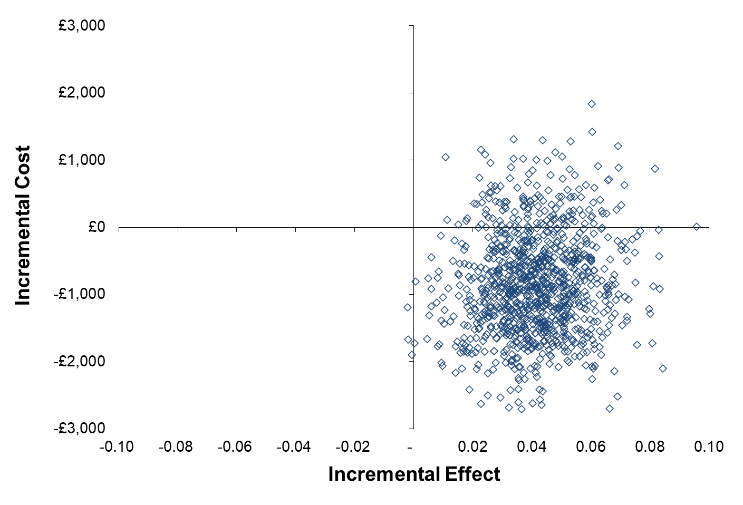 | 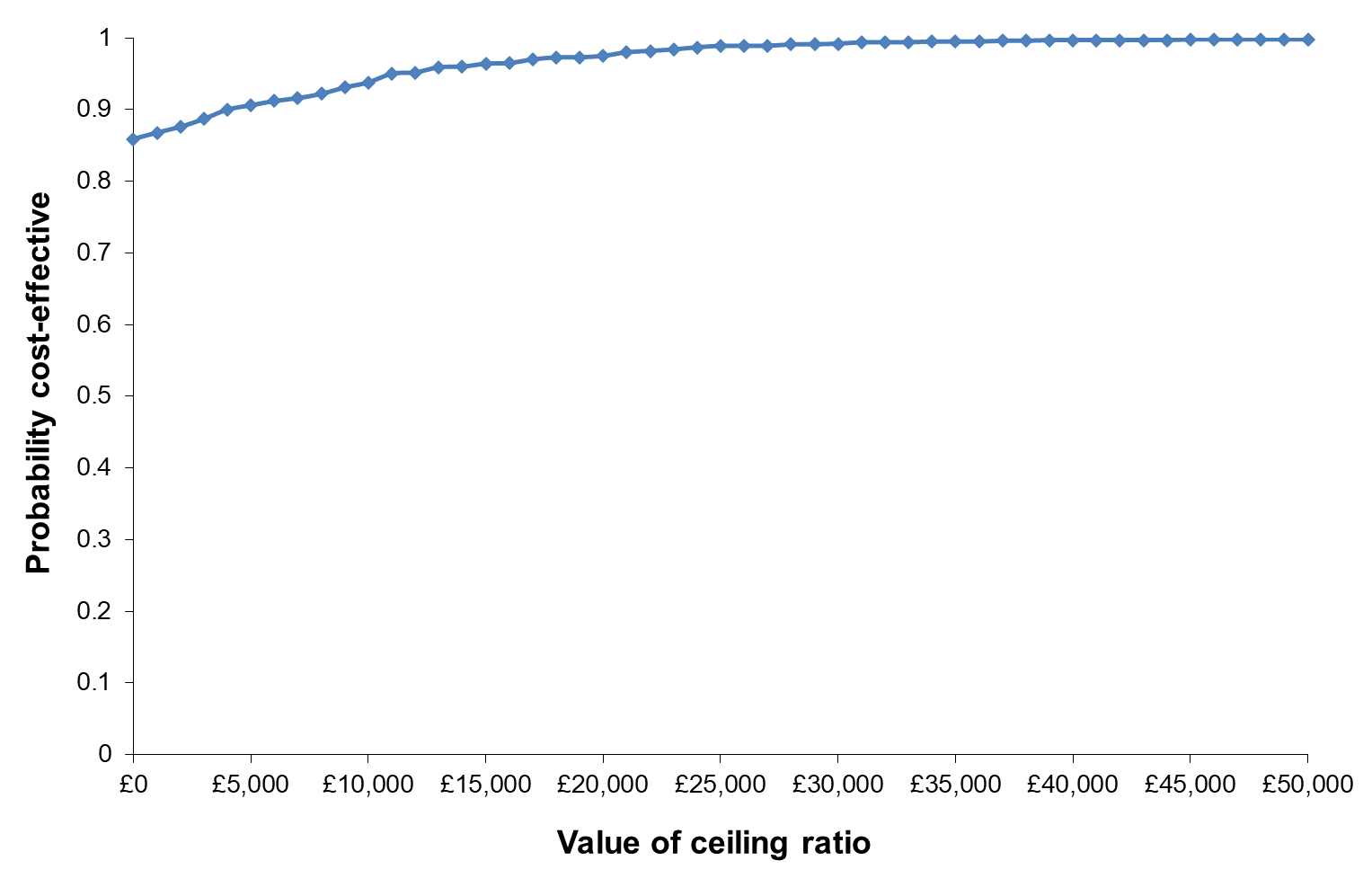 |

Appendix Figure 10. Sensitivity analysis: Cost-effectiveness for CWLY (per protocol)

a

Note: Left: Cost-effectiveness plane with bootstrapped ICERs for pramipexole against placebo treatment presenting results for the per protocol sample for capability-weighted life year (CWLY) gained over 48 weeks from the a) NHS+PSS perspective, and b) societal perspective;

Right: Cost-Effectiveness Acceptability Curve (CEAC) showing the probability of pramipexole being cost-effective in comparison to placebo treatment at different willingness-to-pay thresholds for CWLY gained.

| 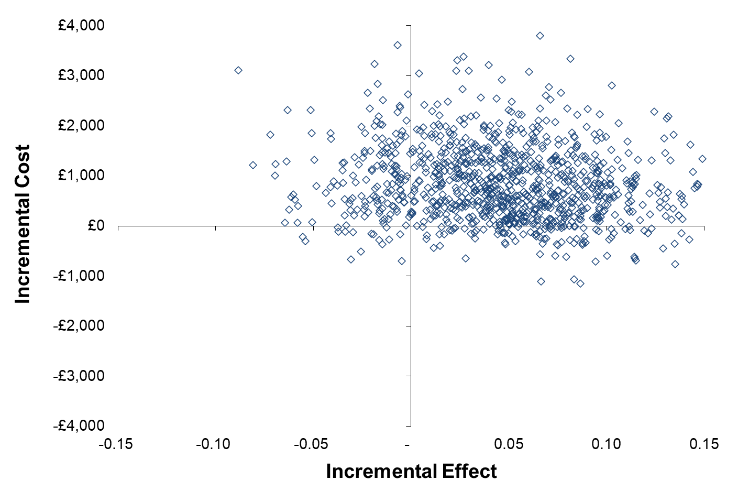  b | 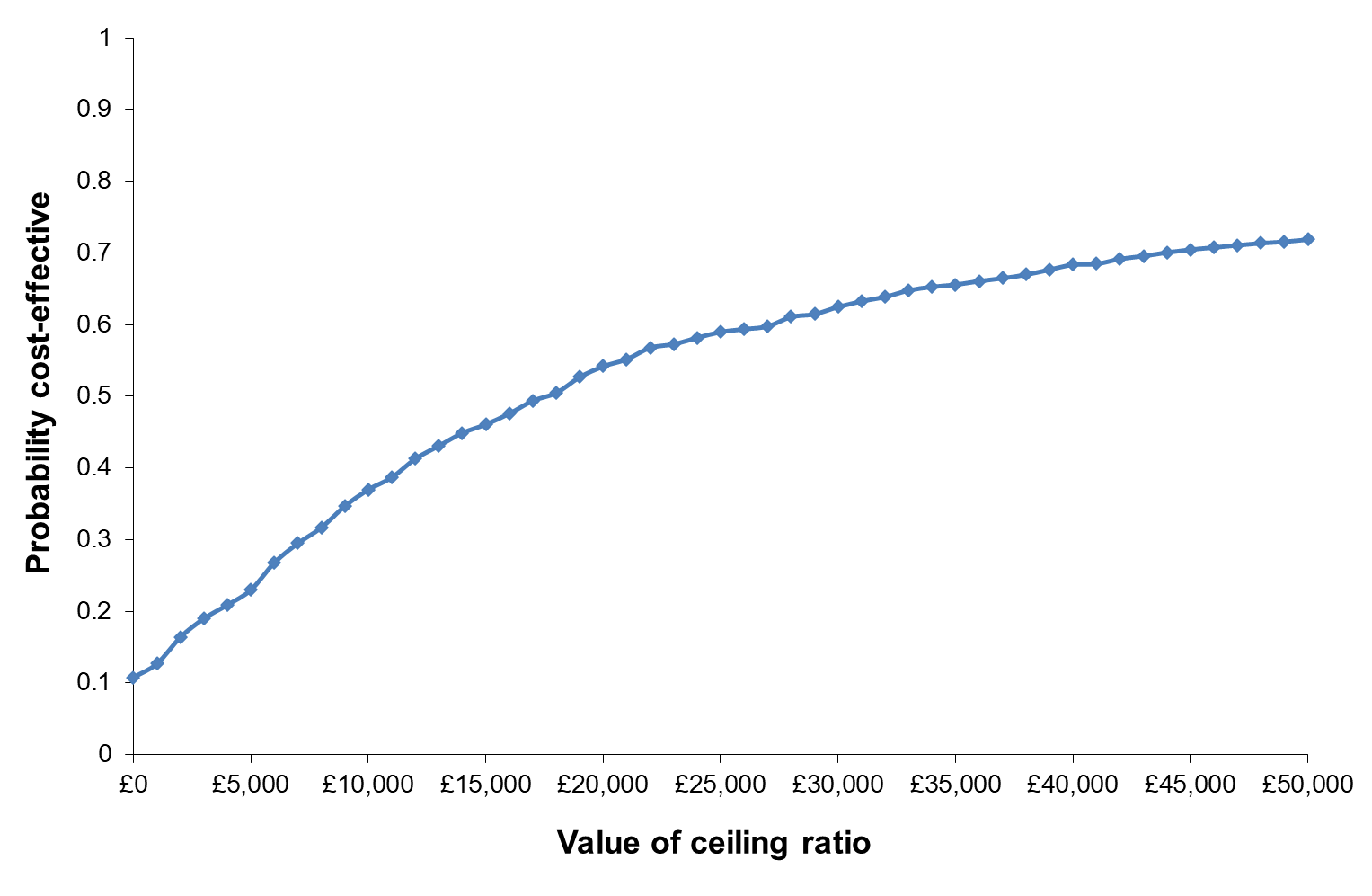 |
| --- | --- |
| 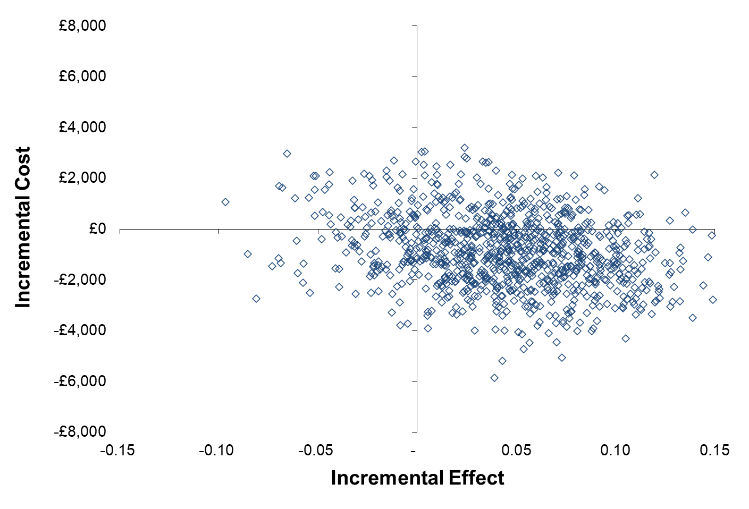 | 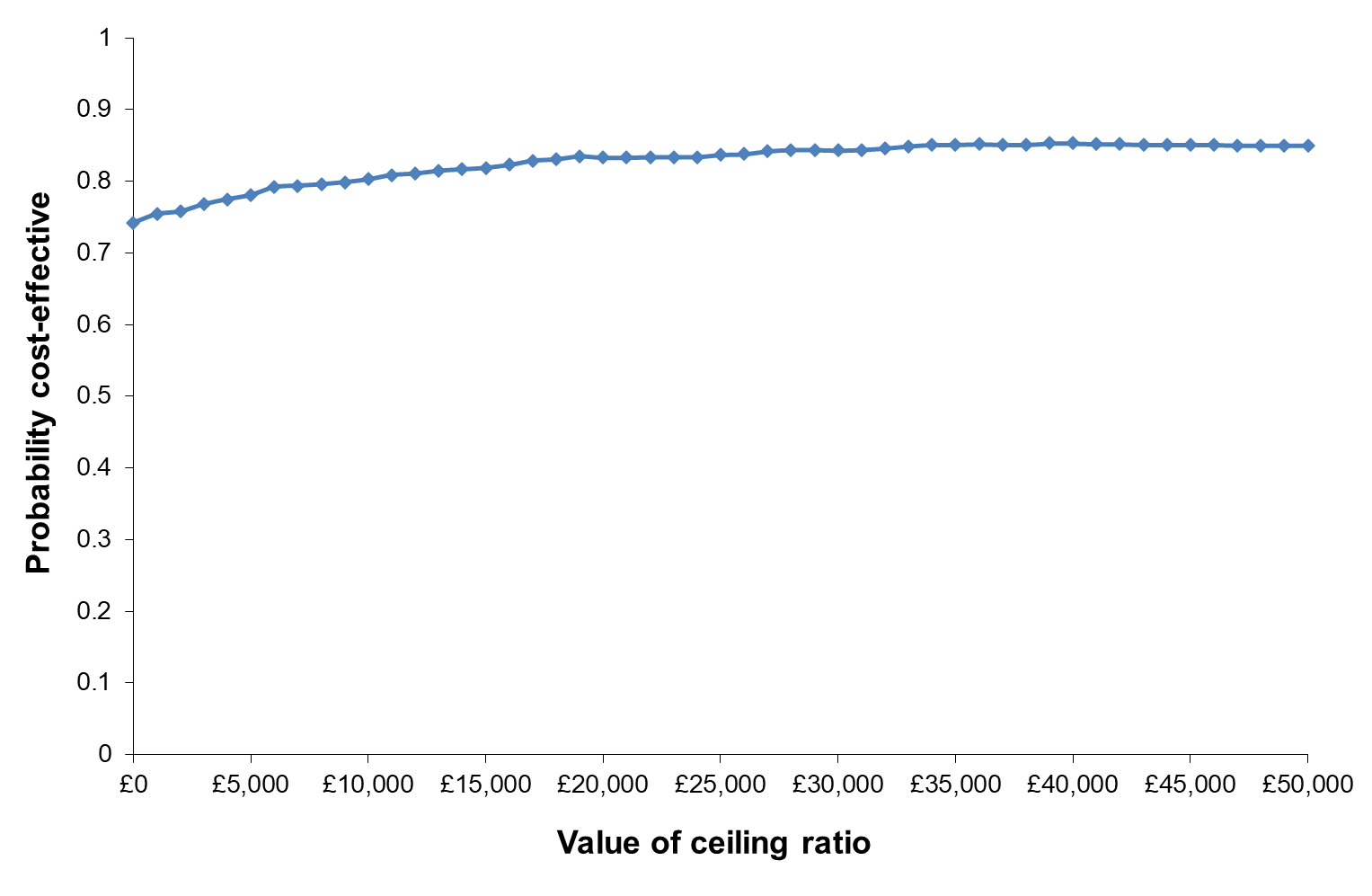 |

Appendix Figure 11. Sensitivity analysis: Cost-effectiveness for QALY (complete cases)

a

Note: Left: Cost-effectiveness plane with bootstrapped ICERs for pramipexole against placebo treatment presenting results for the complete cases sample for quality-adjusted life year (QALY) gained over 48 weeks from the a) NHS+PSS perspective, and b) societal perspective;

Right: Cost-Effectiveness Acceptability Curve (CEAC) showing the probability of pramipexole being cost-effective in comparison to placebo treatment at different willingness-to-pay thresholds for QALY gained.

| 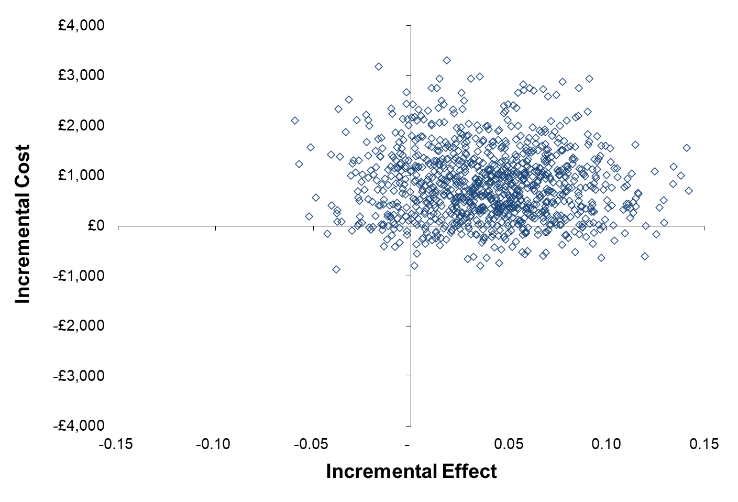  b | 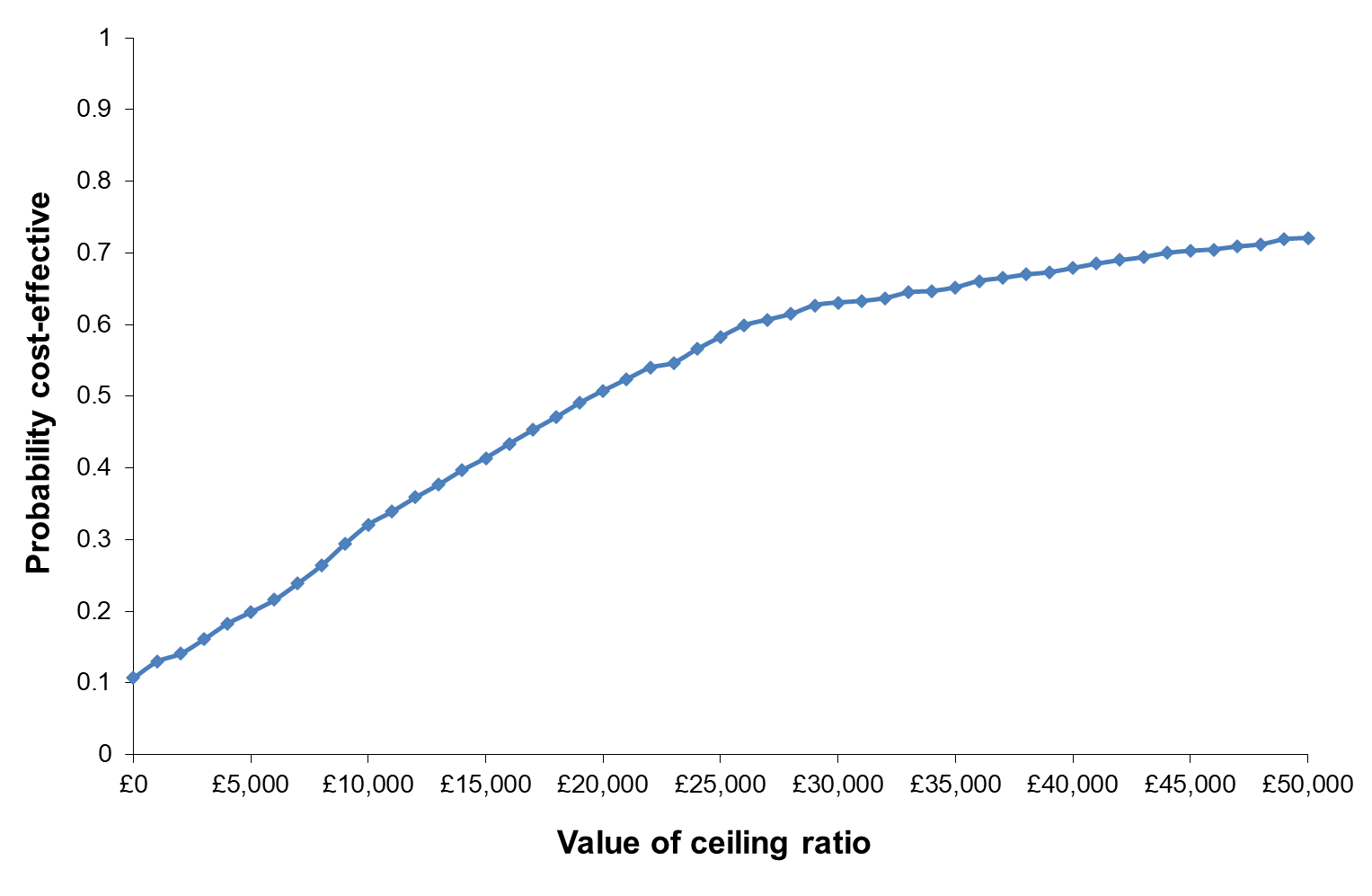 |
| --- | --- |
| 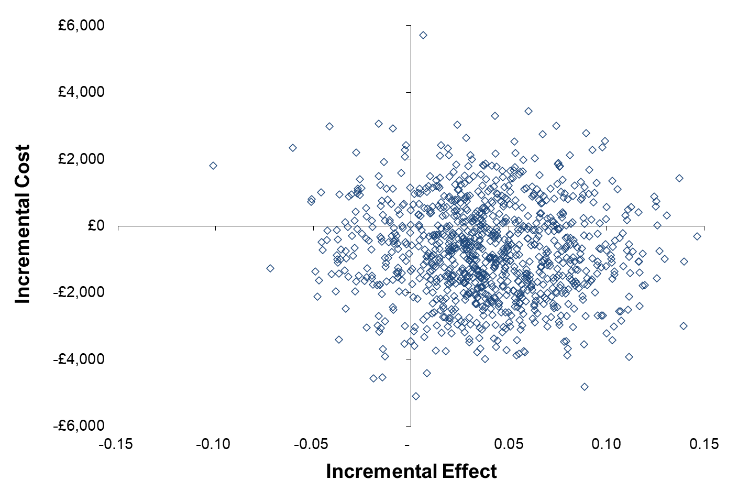 | 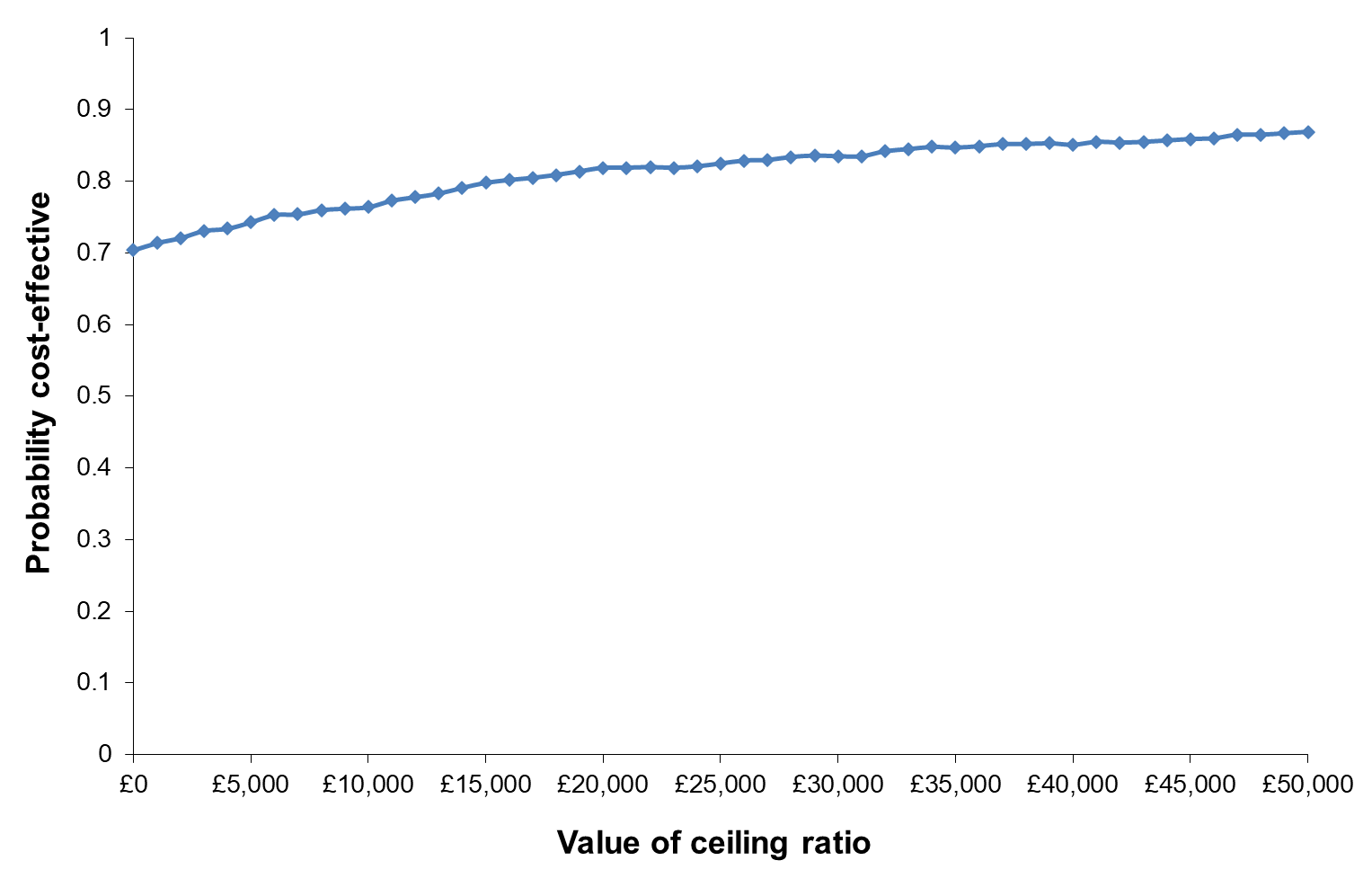 |

Appendix Figure 12. Sensitivity analysis: Cost-effectiveness for YFC (complete cases)

a

Note: Left: Cost-effectiveness plane with bootstrapped ICERs for pramipexole against placebo treatment presenting results for the complete cases sample for year of full capability (YFC) gained over 48 weeks from the a) NHS+PSS perspective, and b) societal perspectives;

Right: Cost-Effectiveness Acceptability Curve (CEAC) showing the probability of pramipexole being cost-effective in comparison to placebo treatment at different willingness-to-pay thresholds for YFC gained.

| 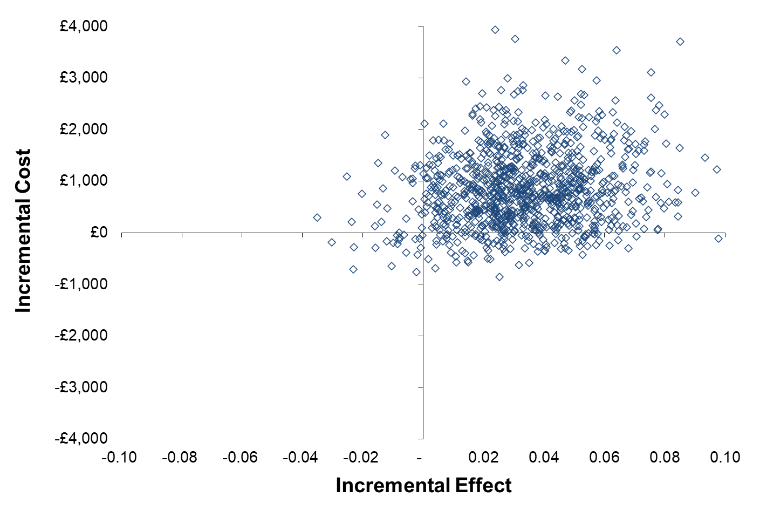  b | 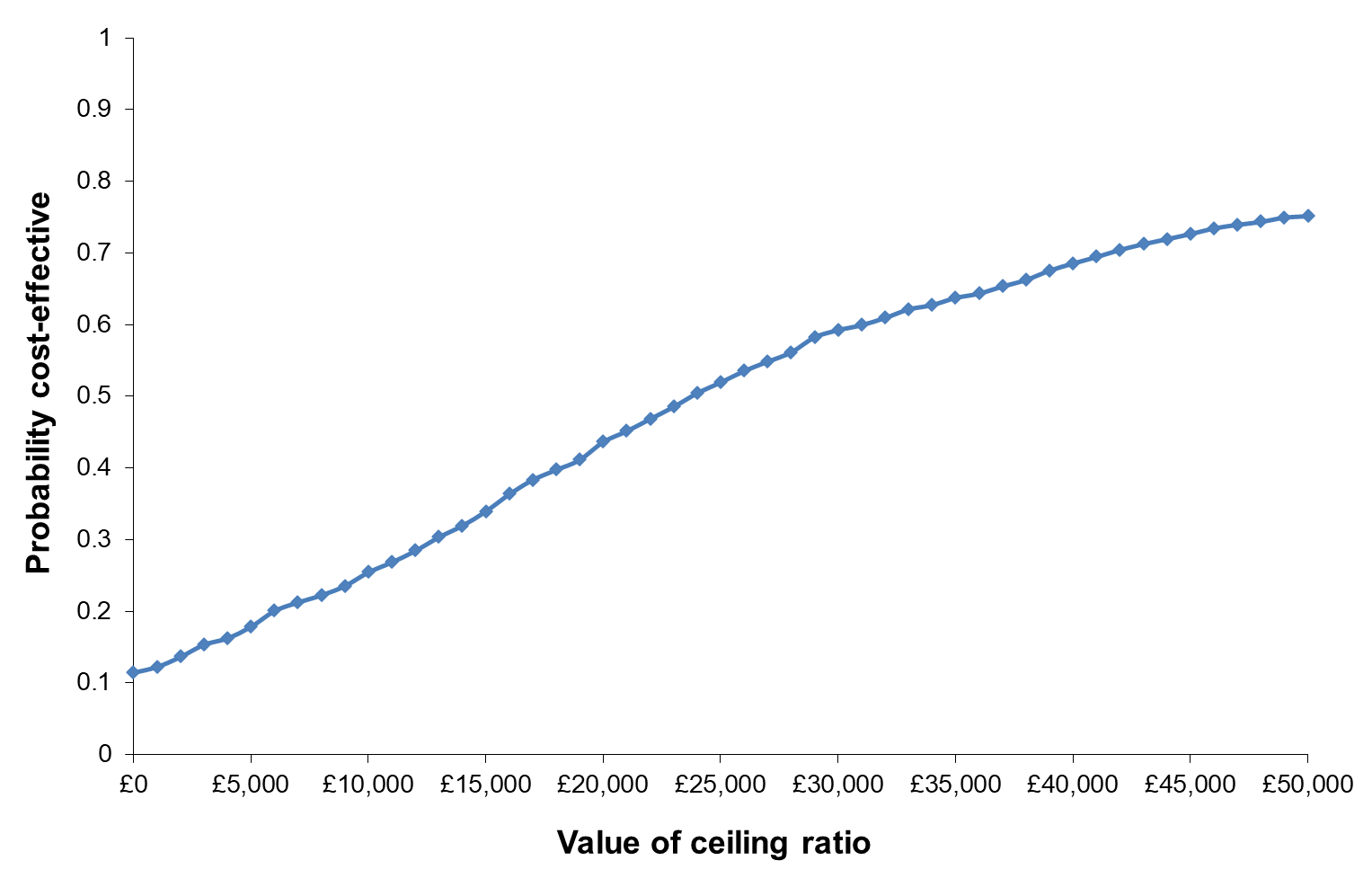 |
| --- | --- |
| 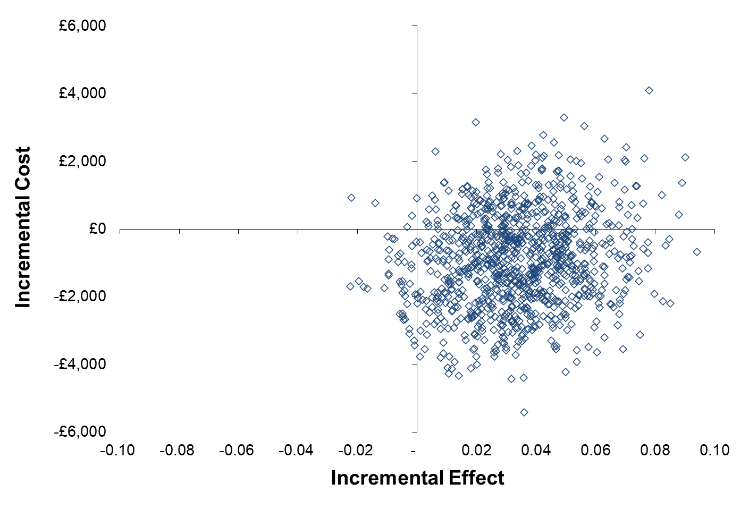 | 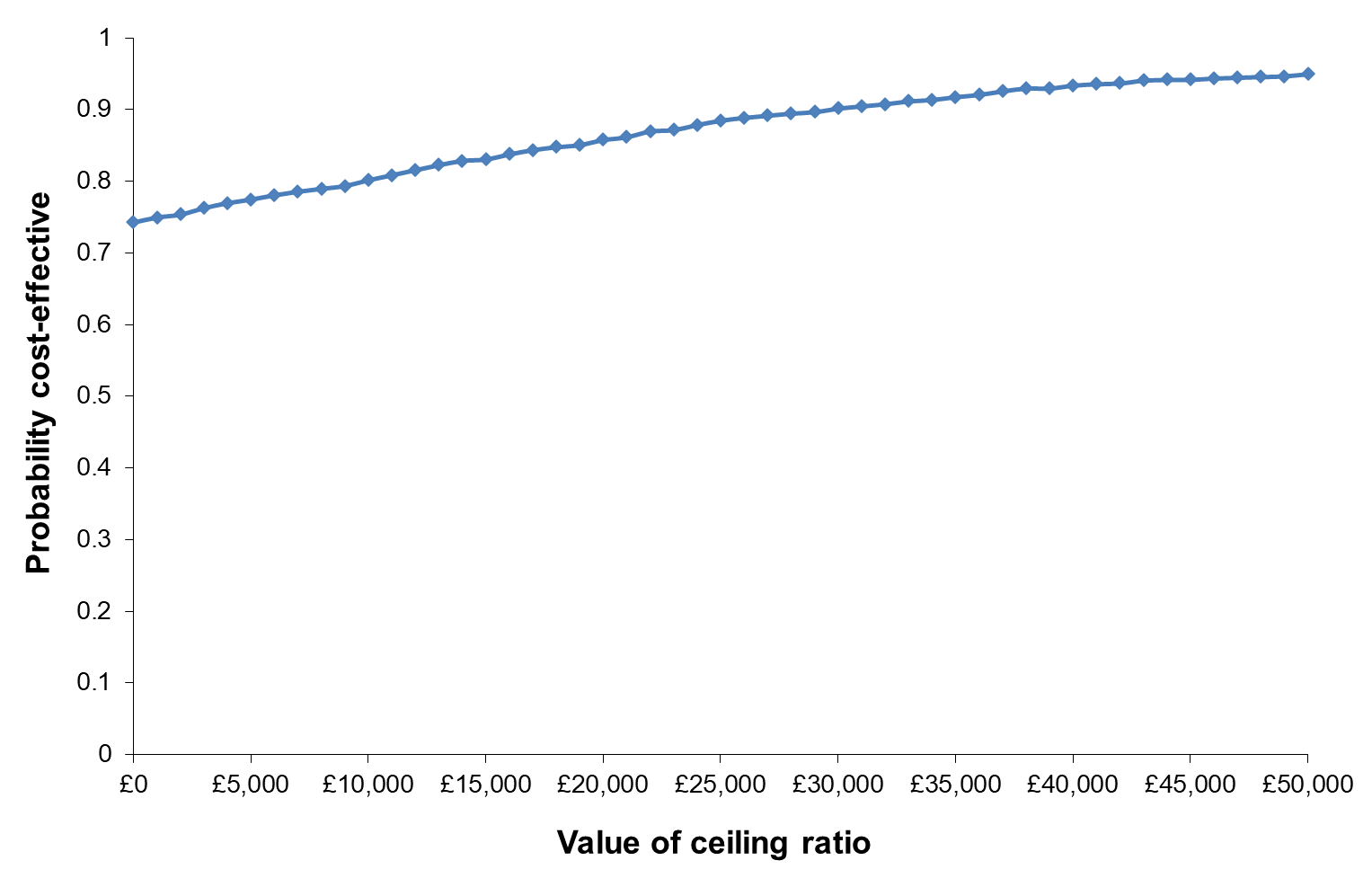 |

Appendix Figure 13. Sensitivity analysis: Cost-effectiveness for CWLY (complete cases)

a

Note: Left: Cost-effectiveness plane with bootstrapped ICERs for pramipexole against placebo treatment presenting results for the complete cases sample for capability-weighted life year (CWLY) gained over 48 weeks from the a) NHS+PSS perspective, and b) societal perspective;

Right: Cost-Effectiveness Acceptability Curve (CEAC) showing the probability of pramipexole being cost-effective in comparison to placebo treatment at different willingness-to-pay thresholds for CWLY gained.

| 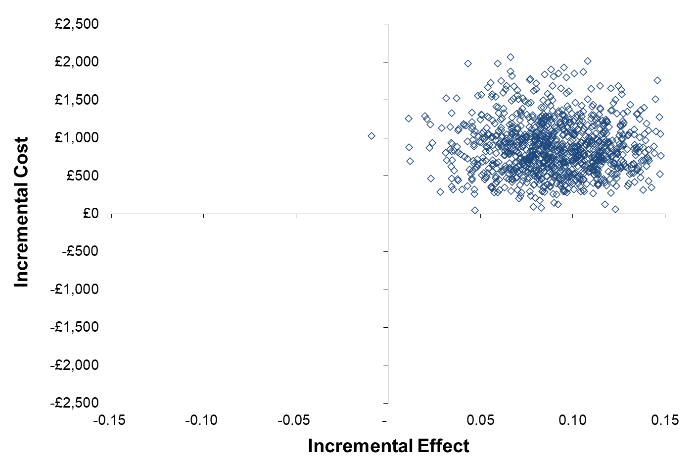  b | 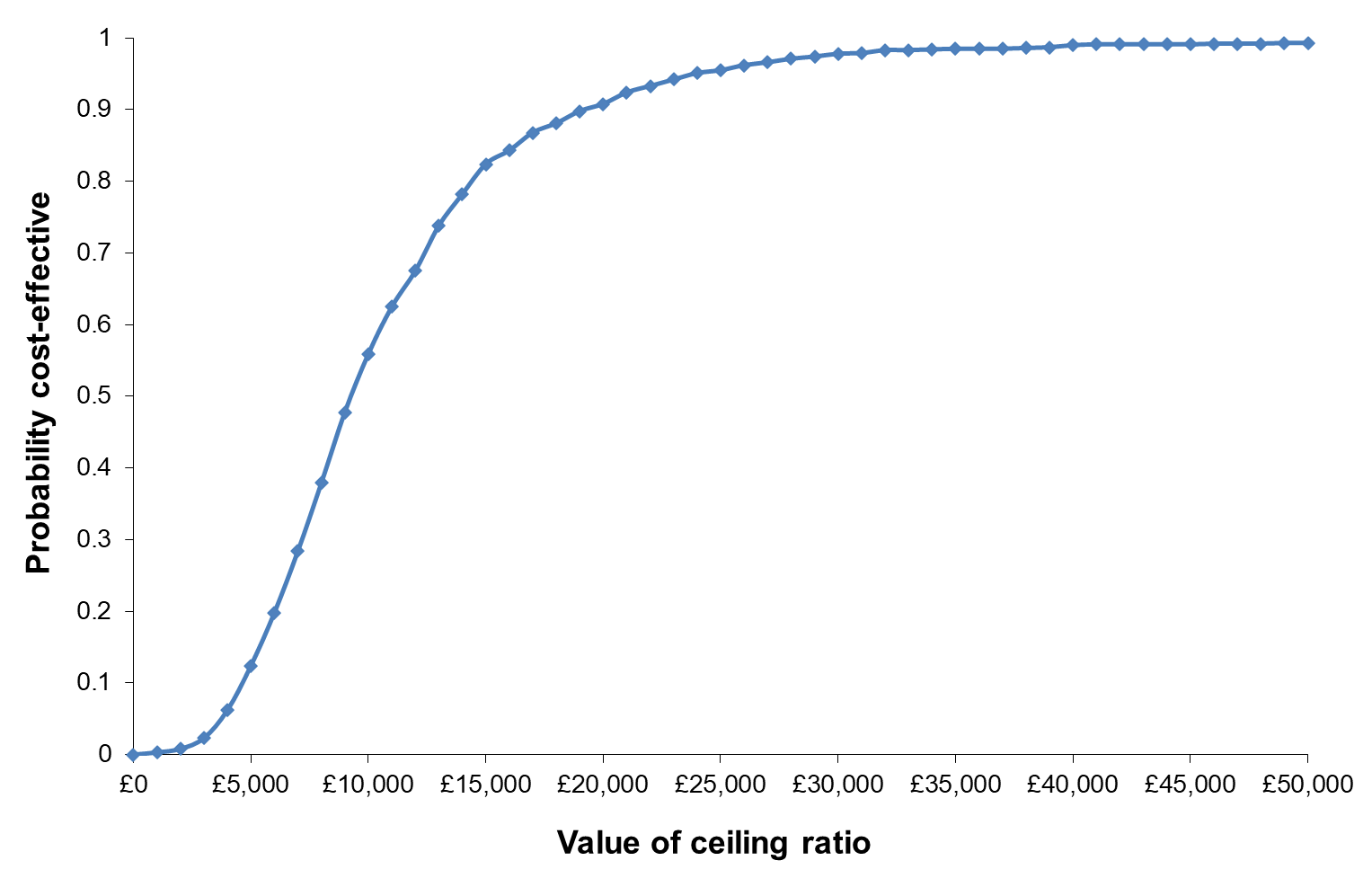 |
| --- | --- |
| 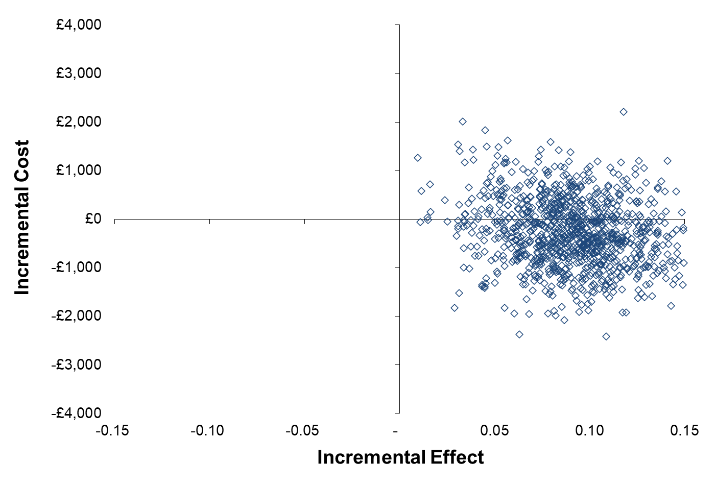 | 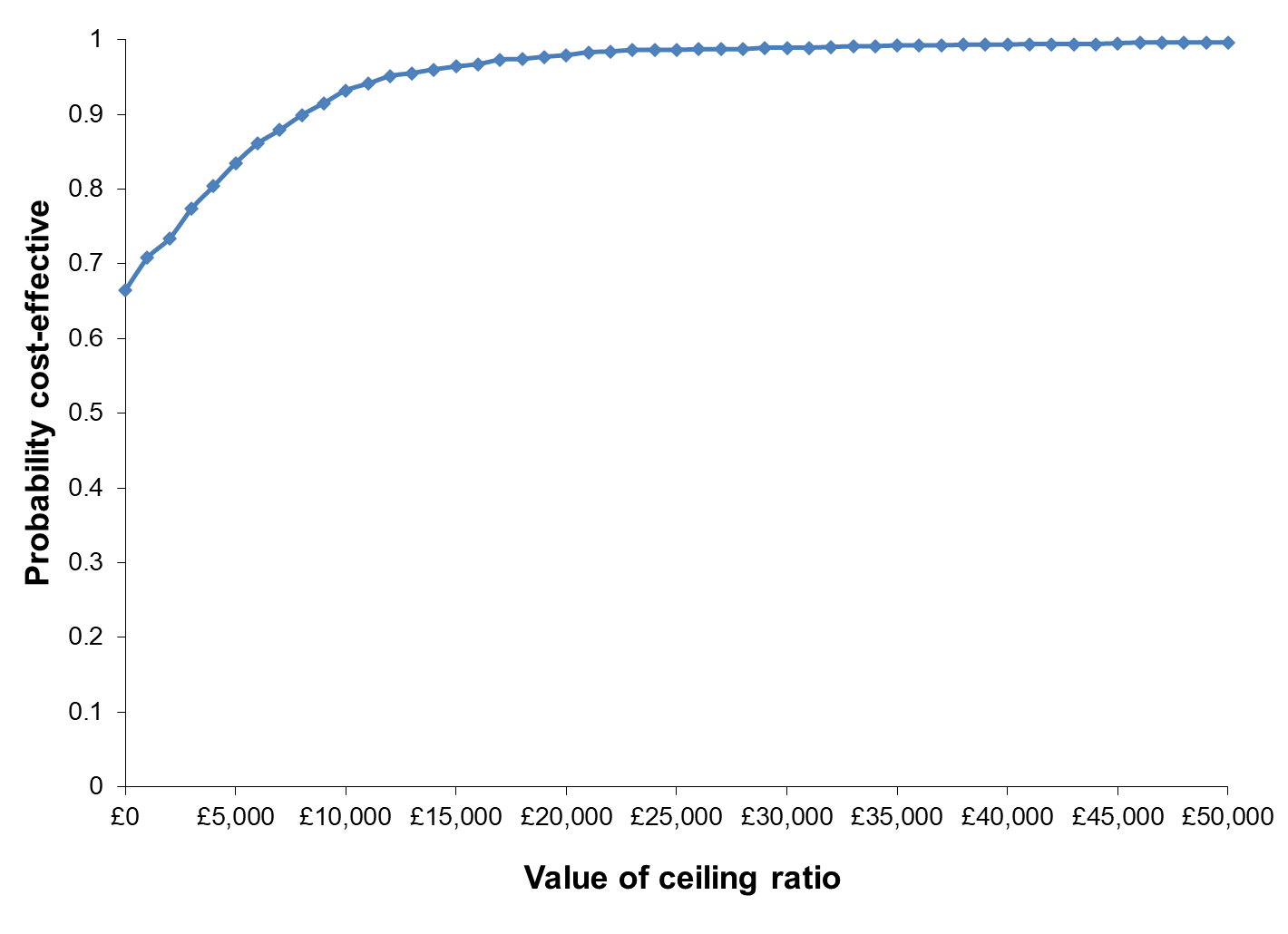 |

Appendix Figure 14. Sensitivity analysis: Cost-effectiveness for QALY (alternative cost)

a

Note: Left: Cost-effectiveness plane with bootstrapped ICERs for pramipexole against placebo treatment presenting results for alternative cost analysis for quality-adjusted life year (QALY) gained over 48 weeks from the a) NHS+PSS perspective, and b) societal perspective;

Right: Cost-Effectiveness Acceptability Curve (CEAC) showing the probability of pramipexole being cost-effective in comparison to placebo treatment at different willingness-to-pay thresholds for QALY gained.

| 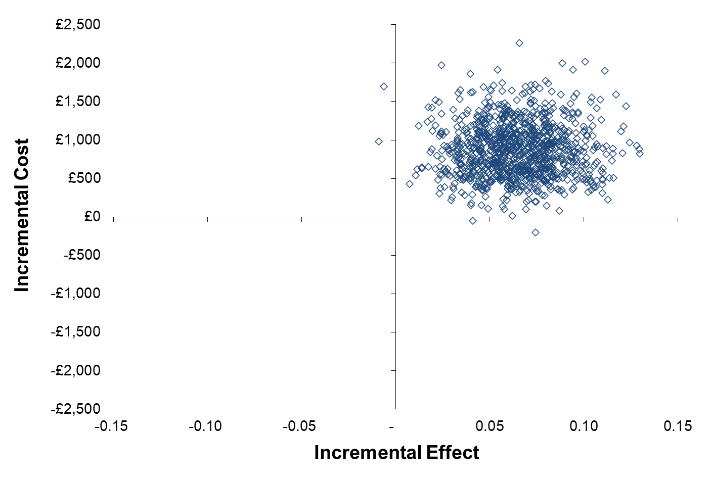  b | 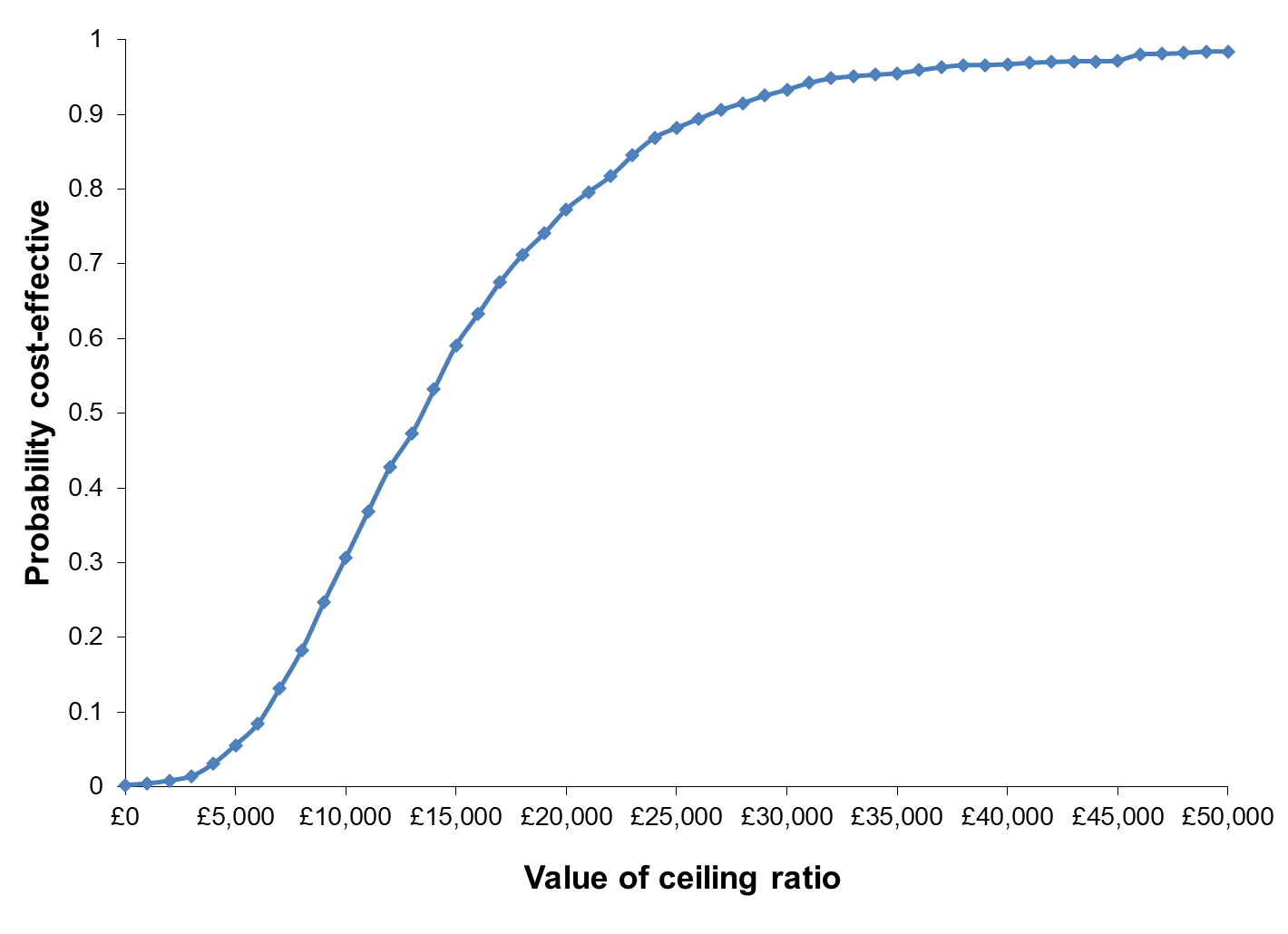 |
| --- | --- |
| 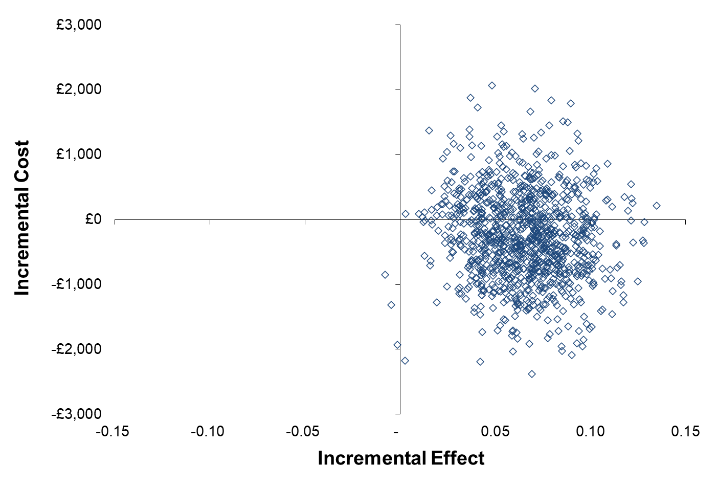 | 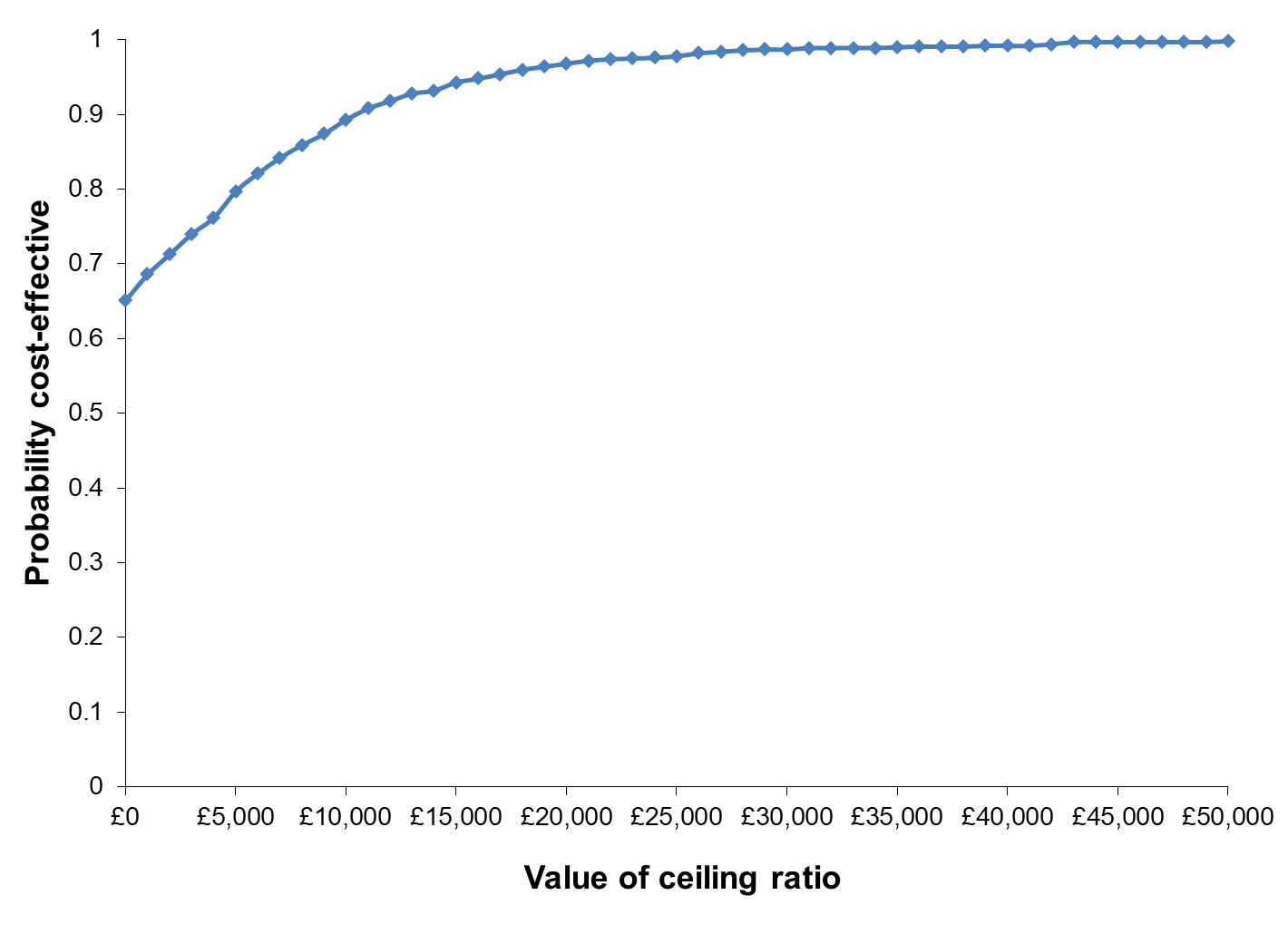 |

Appendix Figure 15. Sensitivity analysis: Cost-effectiveness for YFC (alternative cost)

a

Note: Left: Cost-effectiveness plane with bootstrapped ICERs for pramipexole against placebo treatment presenting results for the alternative cost analysis for year of full capability (YFC) gained over 48 weeks from the a) NHS+PSS perspective, and b) societal perspective;

Right: Cost-Effectiveness Acceptability Curve (CEAC) showing the probability of pramipexole being cost-effective in comparison to placebo treatment at different willingness-to-pay thresholds for YFC gained.

| 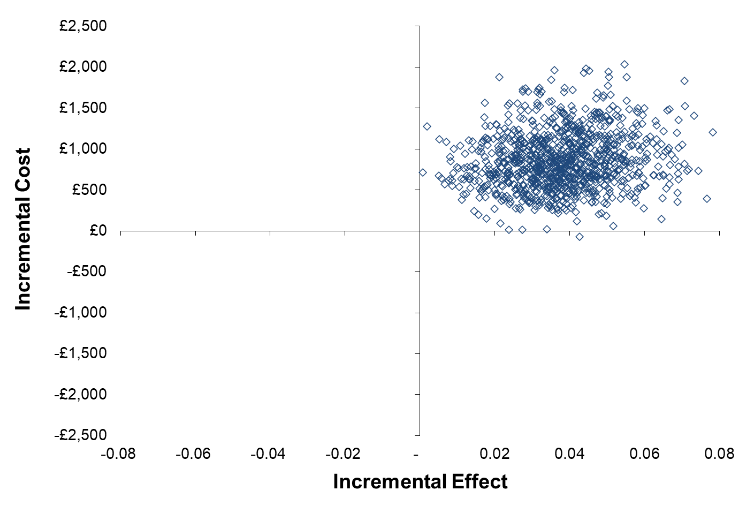  b | 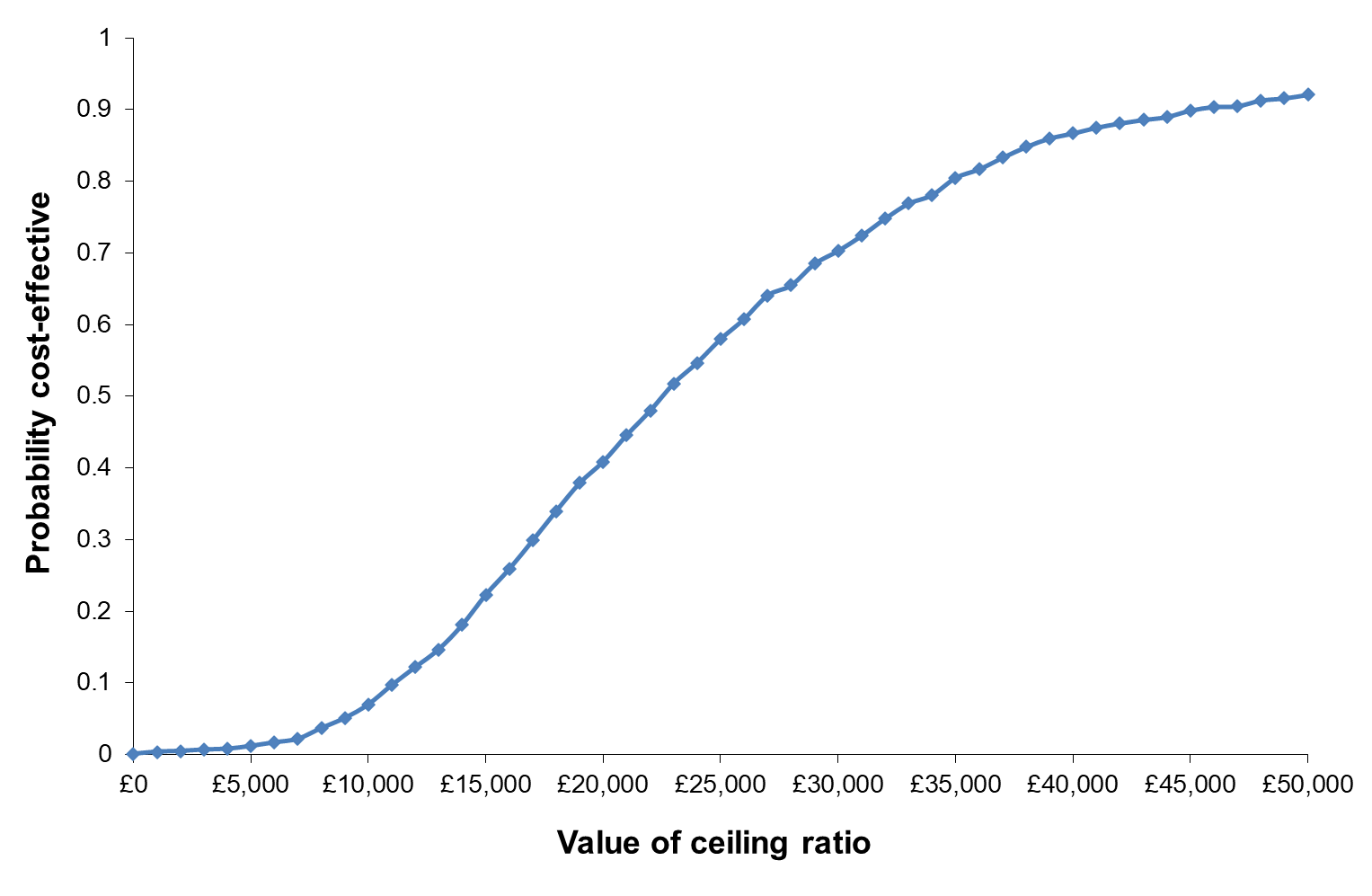 |
| --- | --- |
| 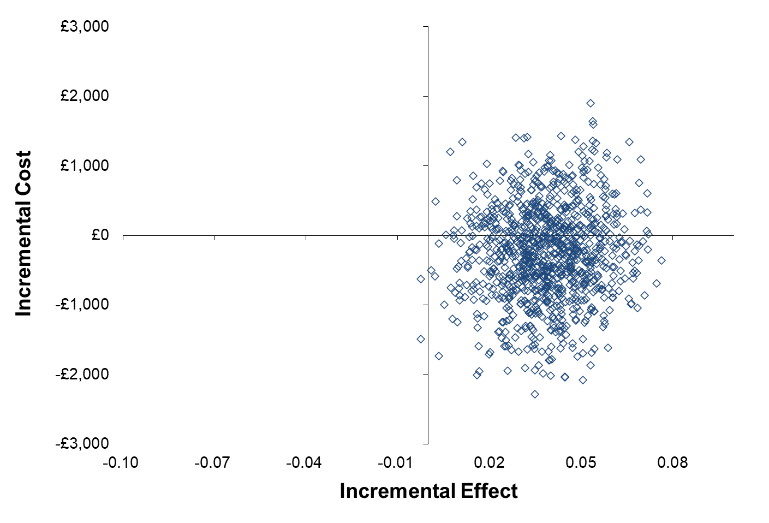 | 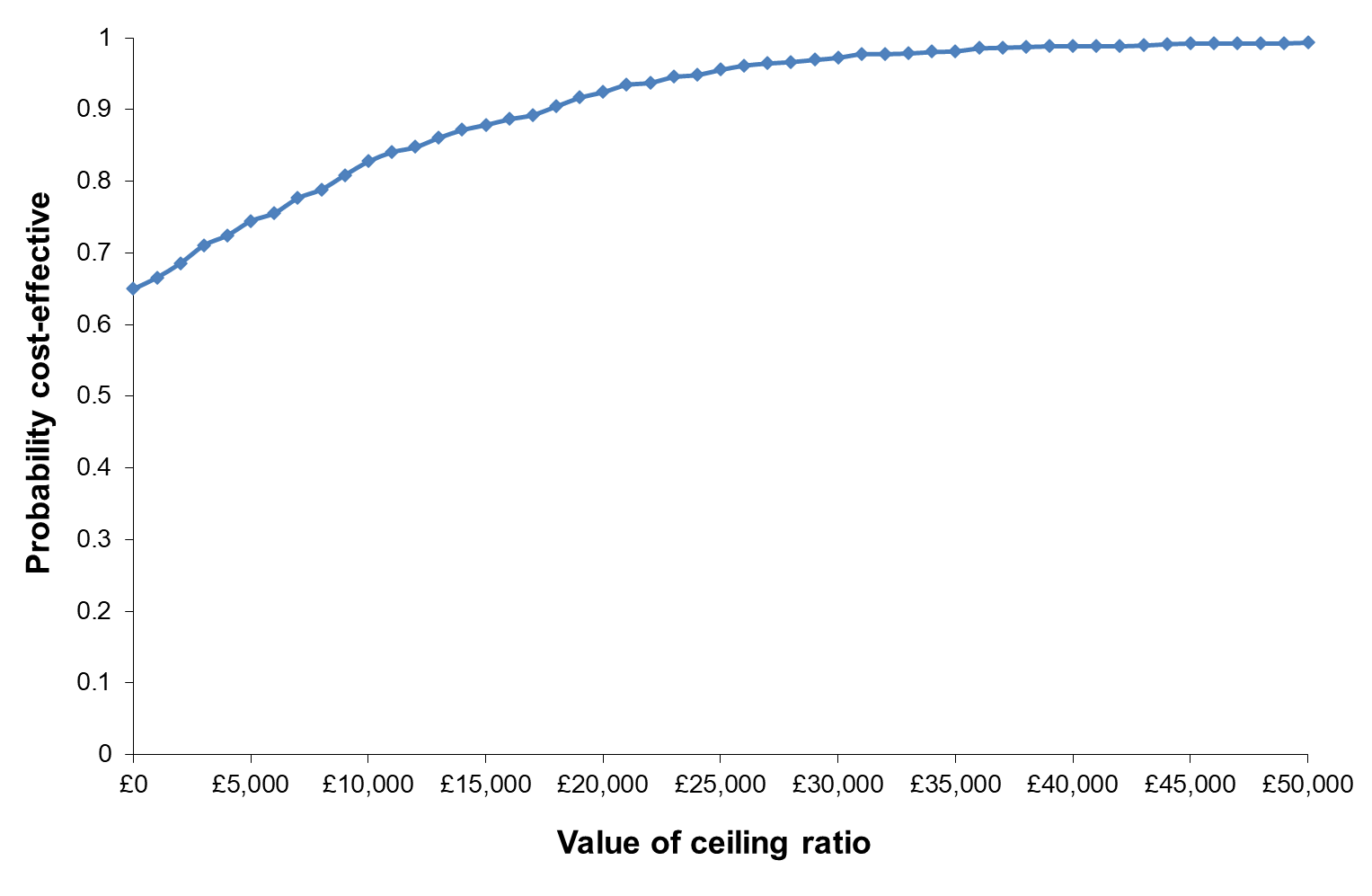 |

Appendix Figure 16. Sensitivity analysis: Cost-effectiveness for CWLY (alternative cost)

a

Note: Left: Cost-effectiveness plane with bootstrapped ICERs for pramipexole against placebo treatment presenting results for the alternative cost analysis for capability-weighted life year (CWLY) gained over 48 weeks from the a) NHS+PSS perspective, and b) societal perspective;

Right: Cost-Effectiveness Acceptability Curve (CEAC) showing the probability of pramipexole being cost-effective in comparison to placebo treatment at different willingness-to-pay thresholds for CWLY gained.

| 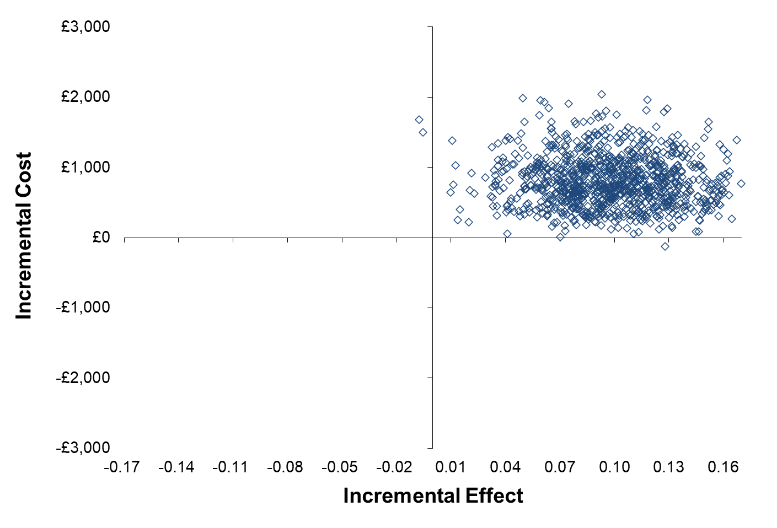  b | 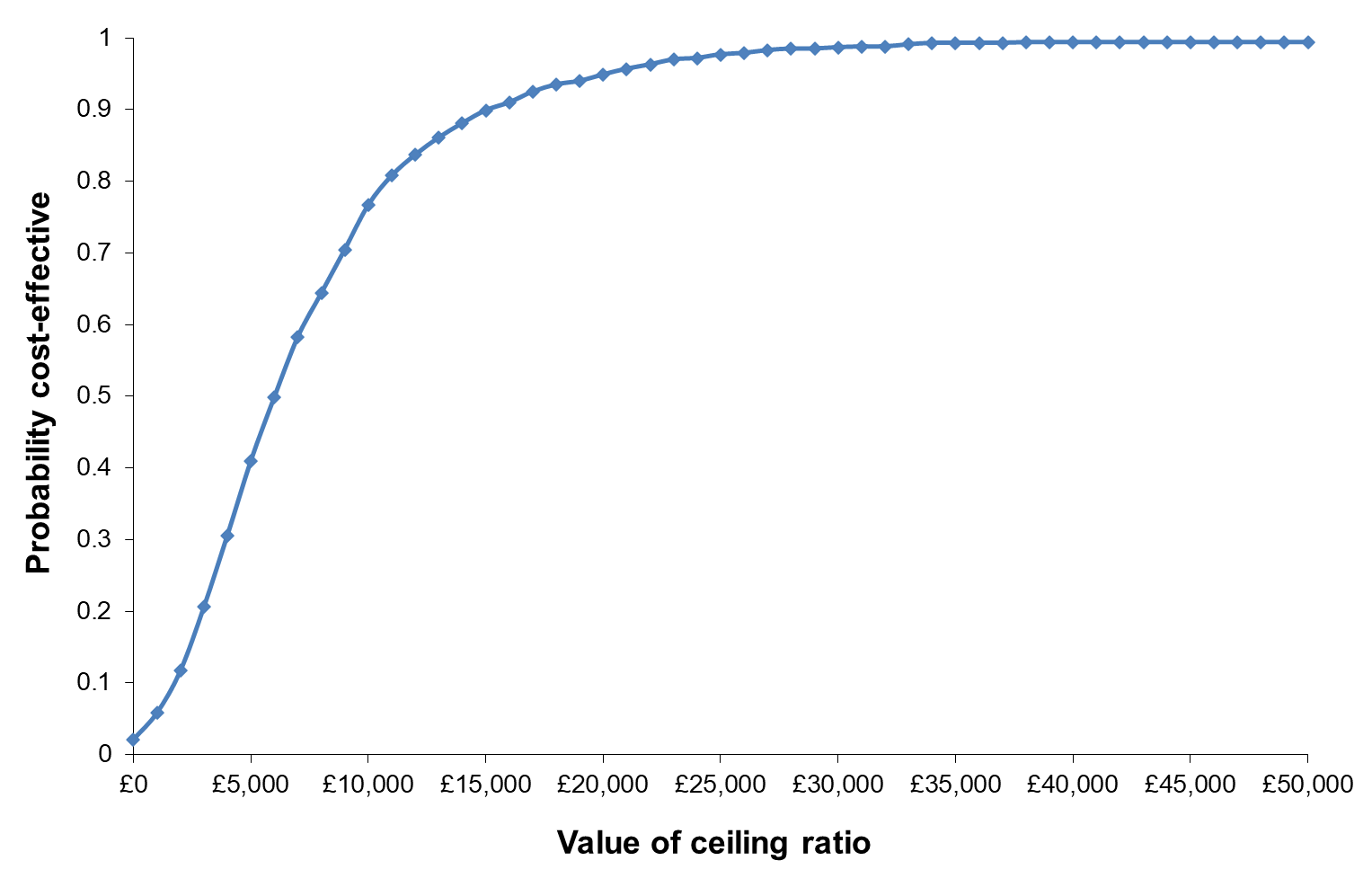 |
| --- | --- |
| 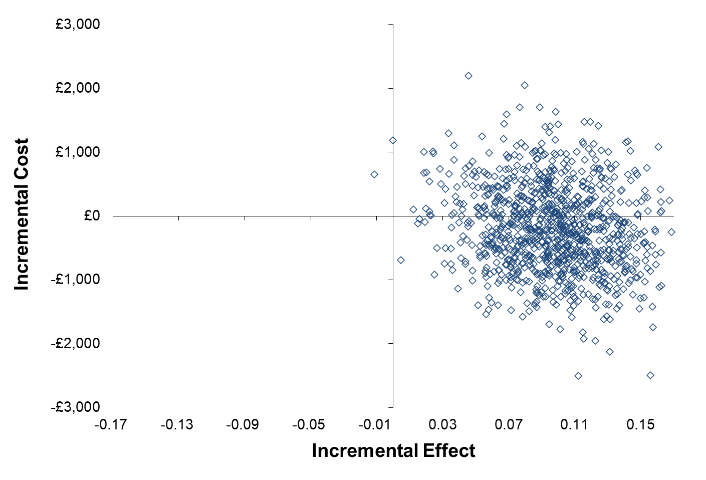 | 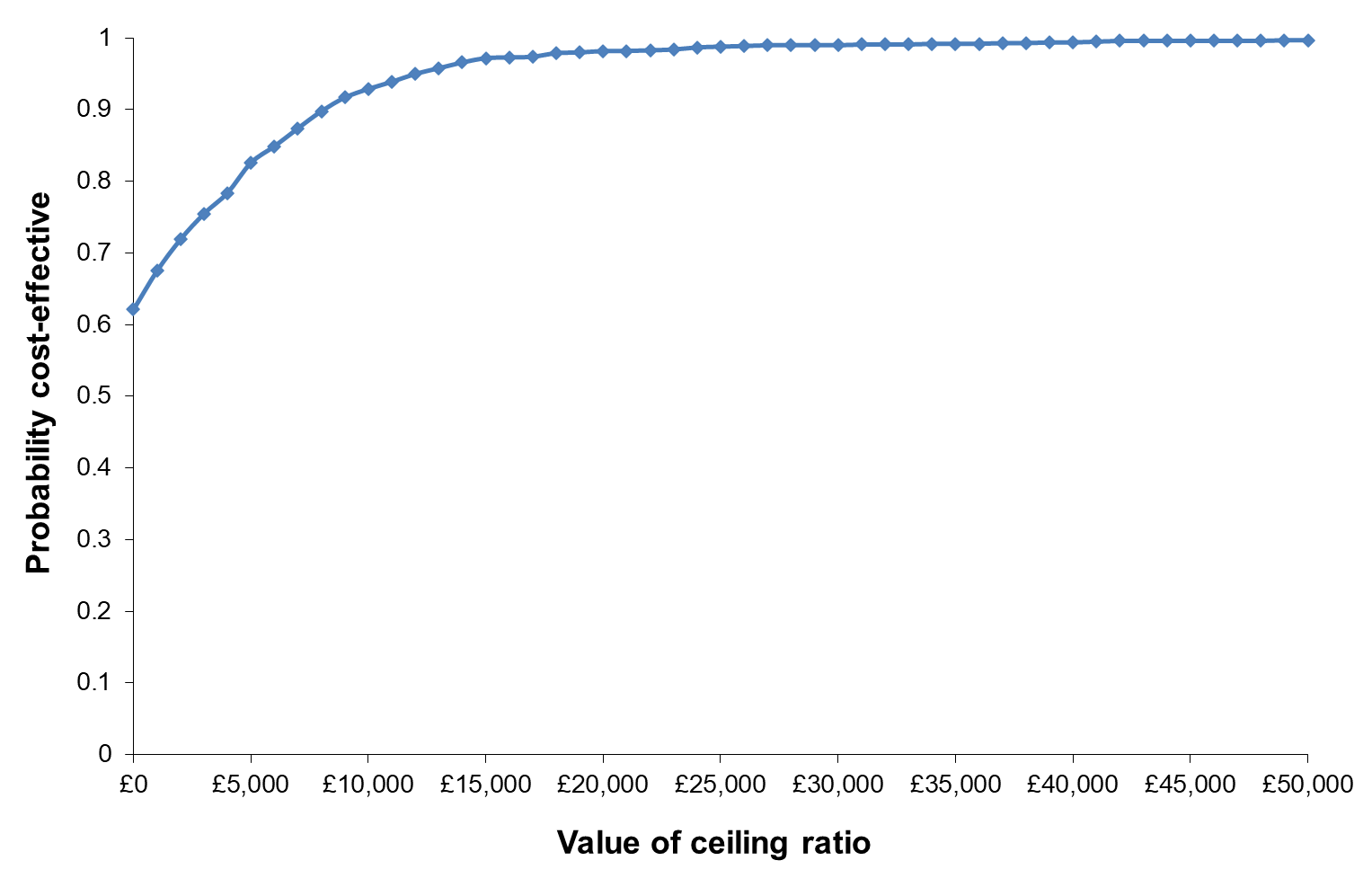 |

Appendix Figure 17. Sensitivity analysis: Cost-effectiveness for QALY (alternative outcome)

a

Note: Left: Cost-effectiveness plane with bootstrapped ICERs for pramipexole against placebo treatment presenting results for the alternative outcome analysis for quality-adjusted life year (QALY) gained over 48 weeks from the a) NHS+PSS perspective, and b) societal perspective;

Right: Cost-Effectiveness Acceptability Curve (CEAC) showing the probability of pramipexole being cost-effective in comparison to placebo treatment at different willingness-to-pay thresholds for QALY gained.

| 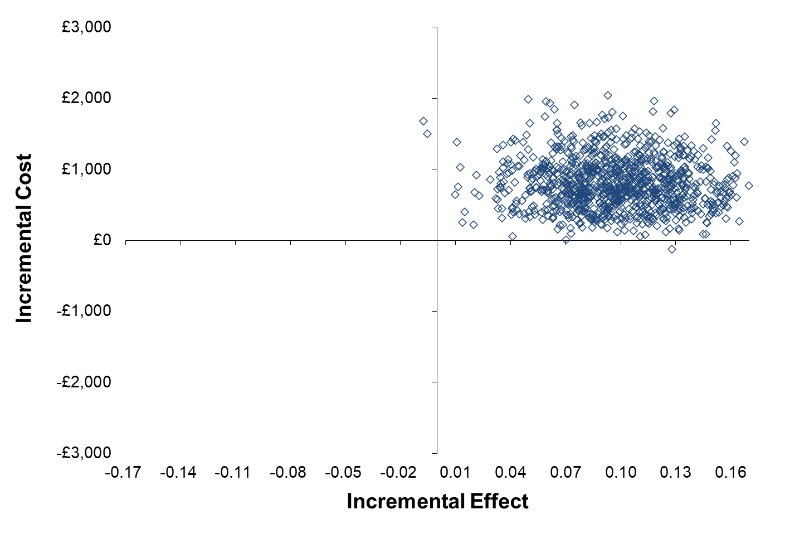  b | 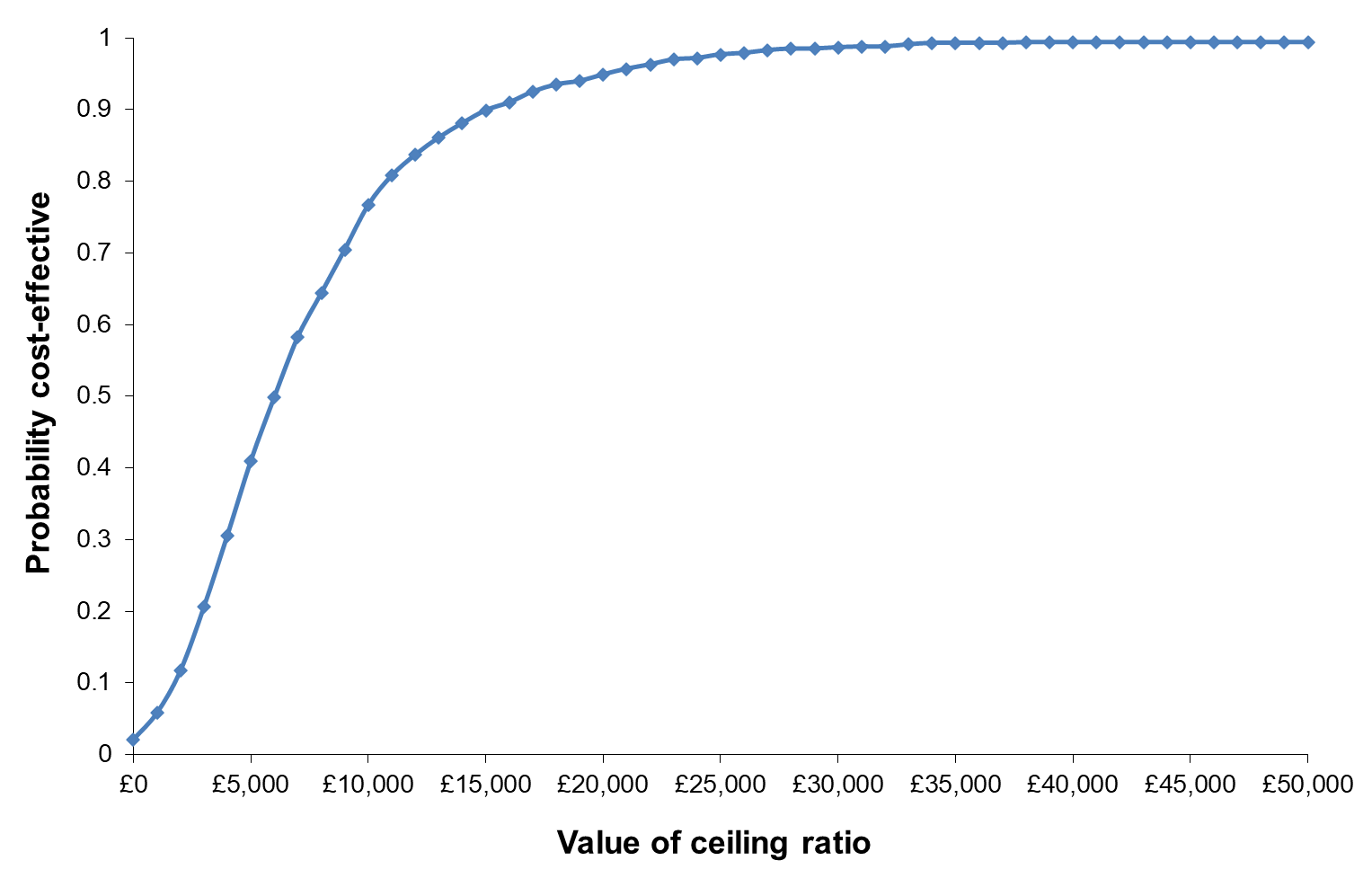 |
| --- | --- |
| 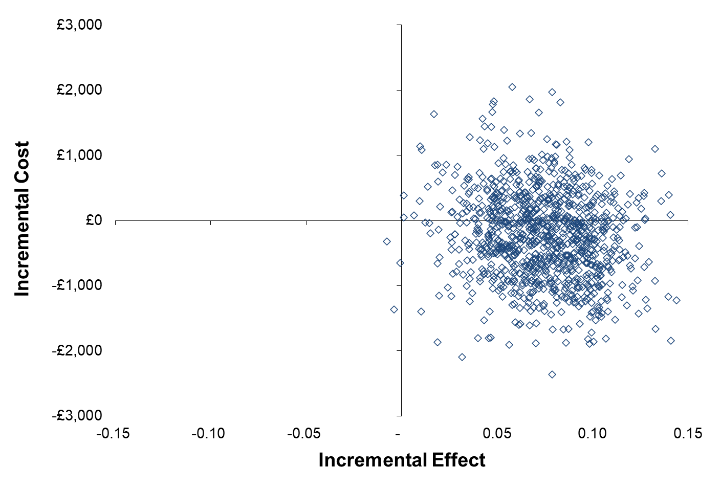 | 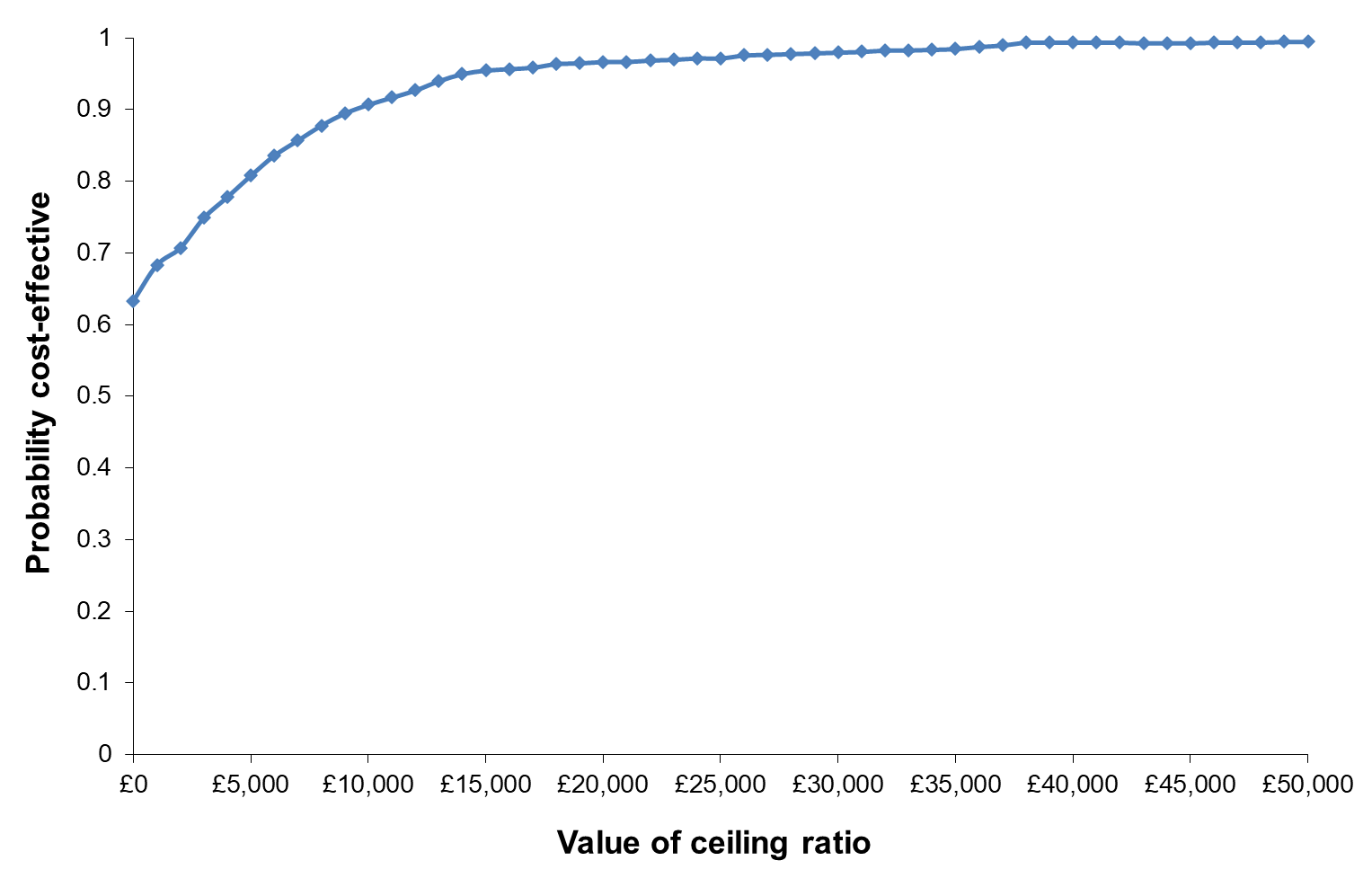 |

Appendix Figure 18. Sensitivity analysis: Cost-effectiveness for YFC (alternative outcome)

a

Note: Left: Cost-effectiveness plane with bootstrapped ICERs for pramipexole against placebo treatment presenting results for the alternative outcome analysis for year of full capability (YFC) gained over 48 weeks from the a) NHS+PSS perspective, and b) societal perspective;

Right: Cost-Effectiveness Acceptability Curve (CEAC) showing the probability of pramipexole being cost-effective in comparison to placebo treatment at different willingness-to-pay thresholds for YFC gained.

| 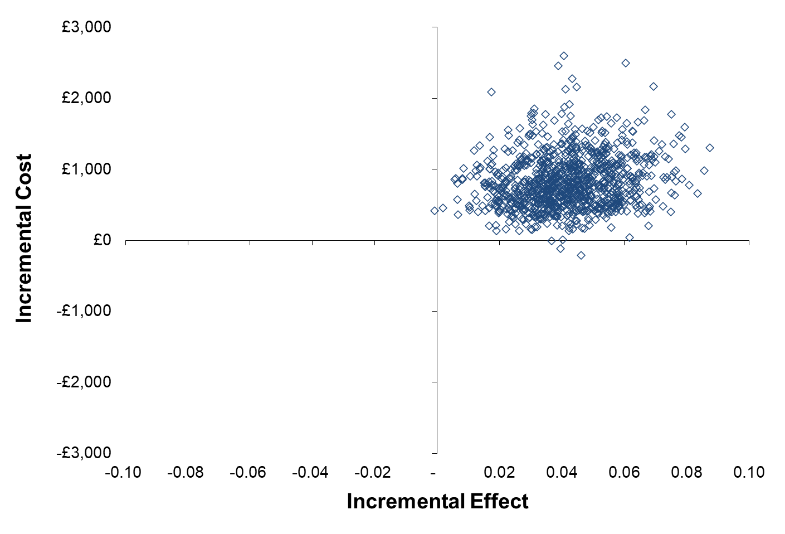  b | 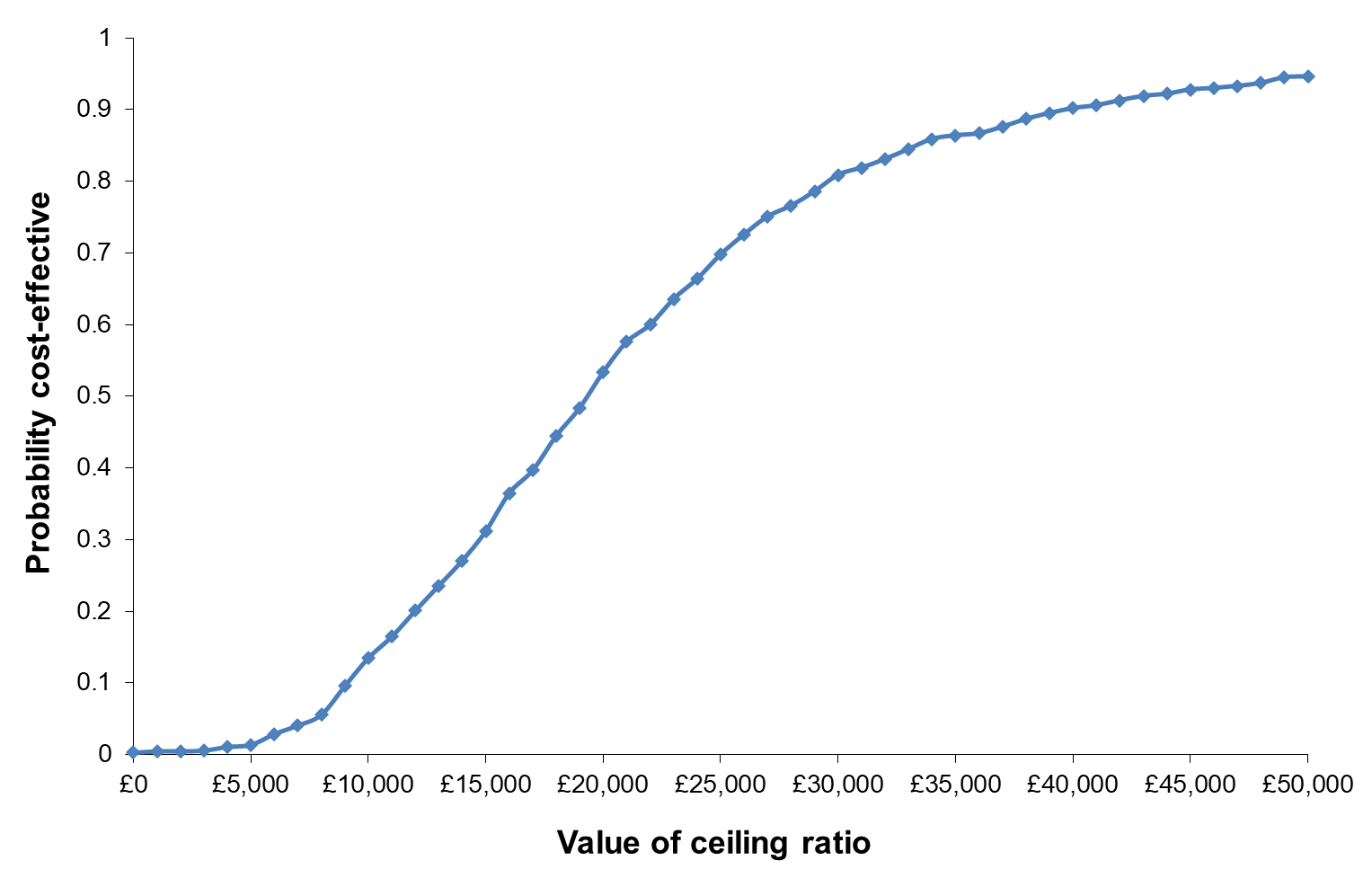 |
| --- | --- |
| 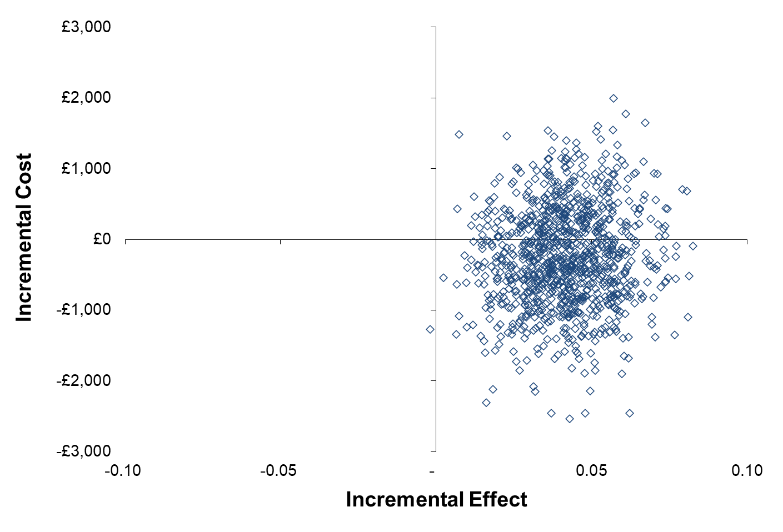 | 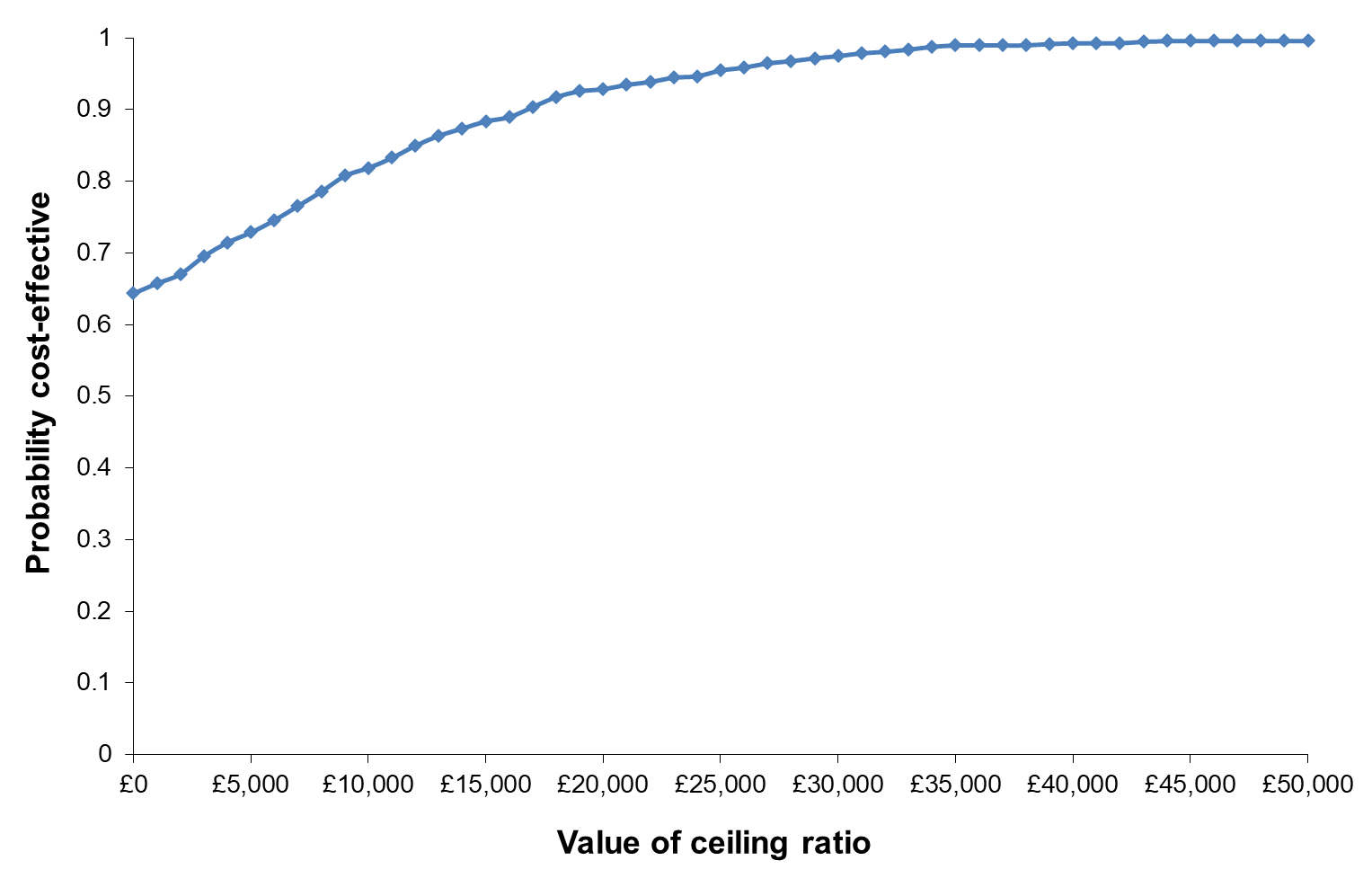 |

Appendix Figure 19. Sensitivity analysis: Cost-effectiveness for CWLY (alternative outcome)

a

Note: Left: Cost-effectiveness plane with bootstrapped ICERs for pramipexole against placebo treatment presenting results for the alternative outcome analysis for capability-weighted life year (CWLY) gained over 48 weeks from the a) NHS+PSS perspective, and b) societal perspective;

Right: Cost-Effectiveness Acceptability Curve (CEAC) showing the probability of pramipexole being cost-effective in comparison to placebo treatment at different willingness-to-pay thresholds for CWLY gained.

| 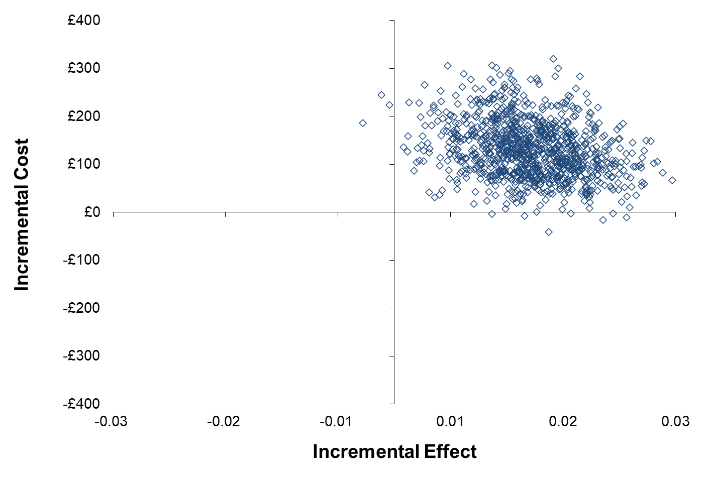  b | 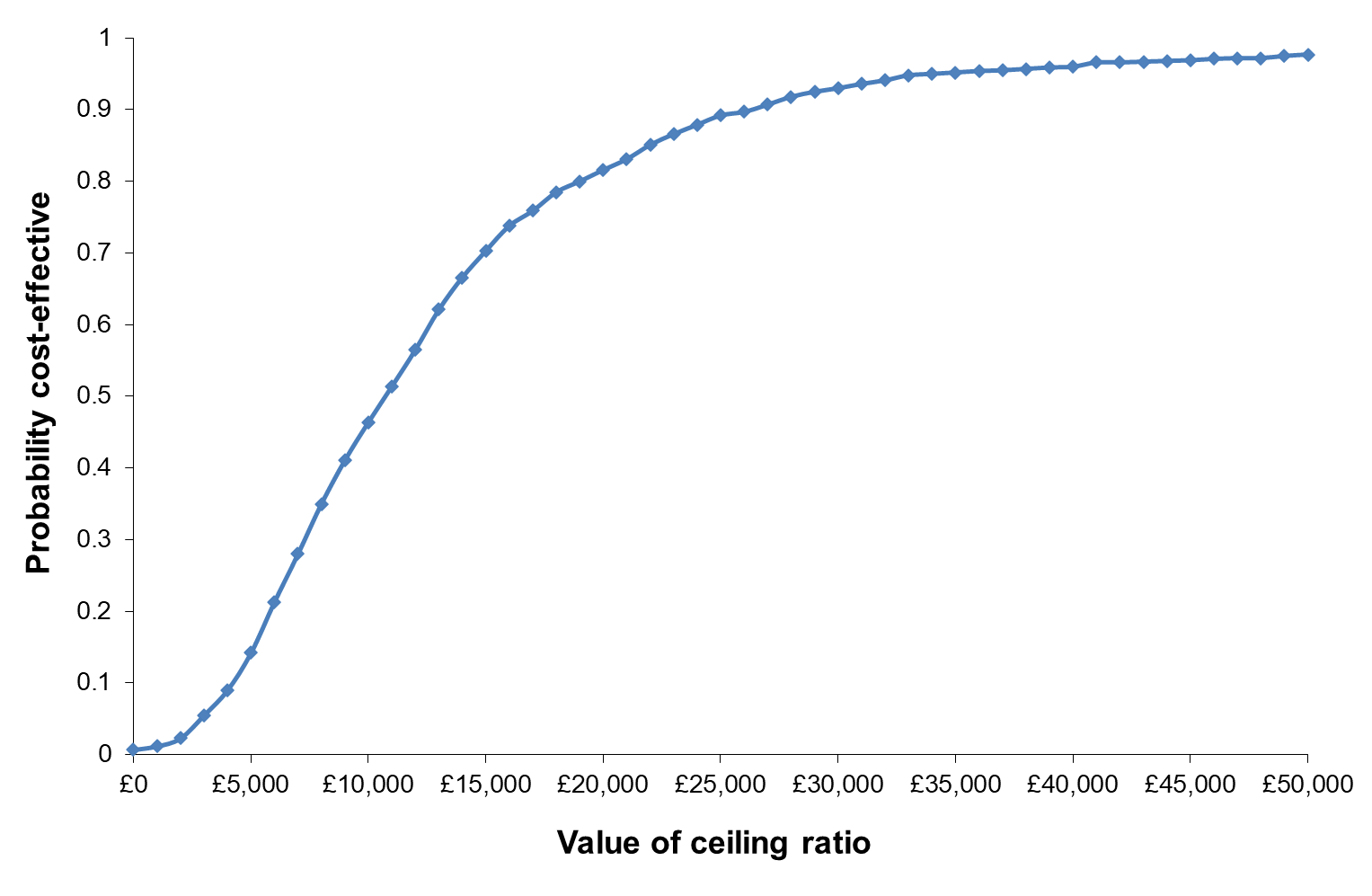 |
| --- | --- |
| 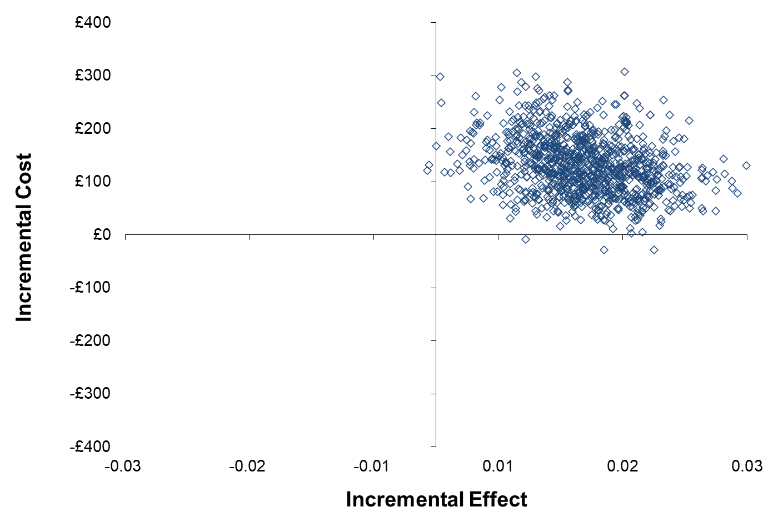 | 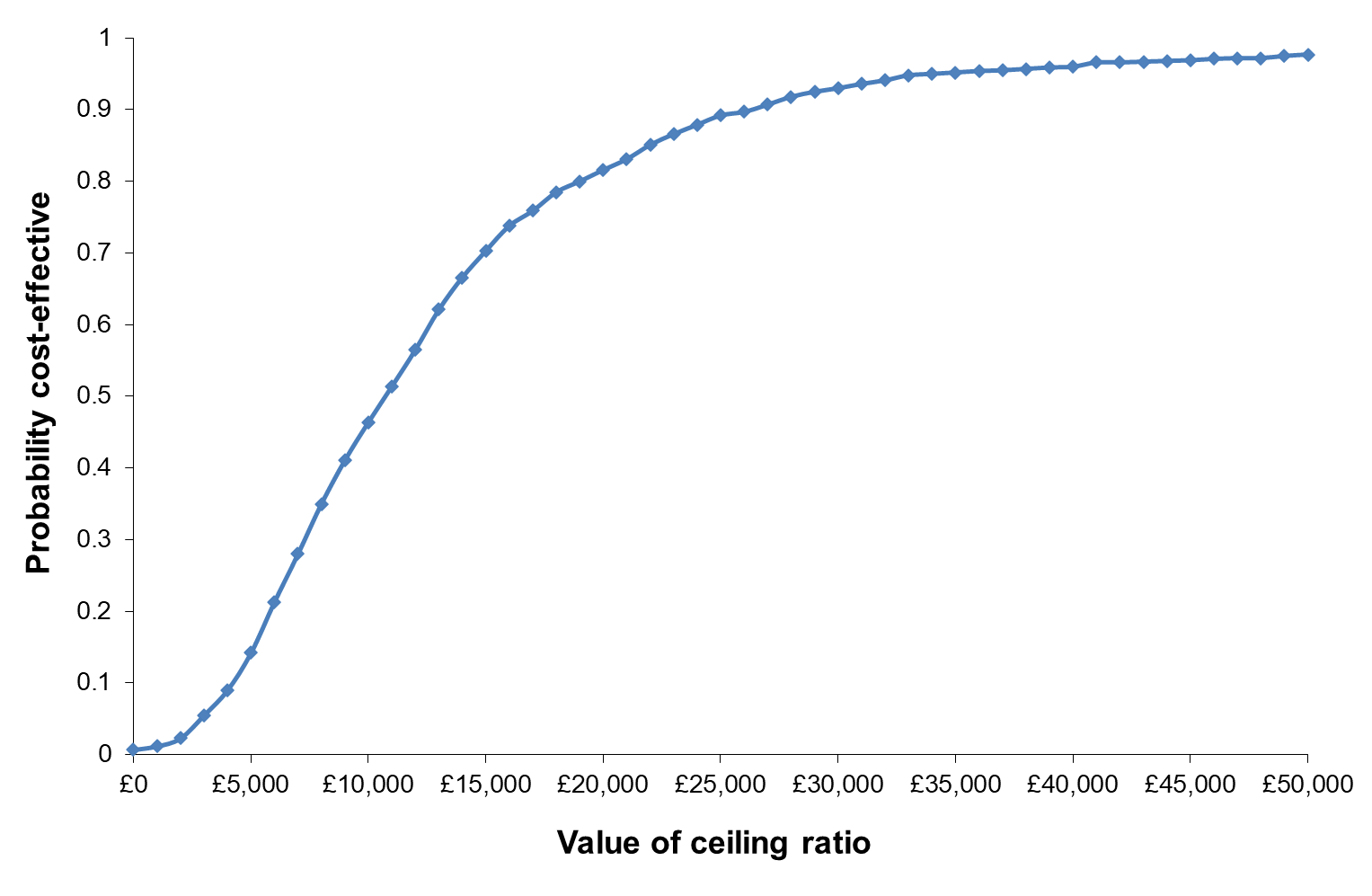 |

a

Appendix Figure 20. Sensitivity analysis: Cost-effectiveness for QALY over 12 weeks (current pramipexole cost)

Note: Left: Cost-effectiveness plane with bootstrapped ICERs for pramipexole against placebo treatment presenting results for the alternative pramipexole cost analysis for quality-adjusted life (QALY) gained over 12 weeks from the a) NHS+PSS perspective, and b) societal perspective;

Right: Cost-Effectiveness Acceptability Curve (CEAC) showing the probability of pramipexole being cost-effective in comparison to placebo treatment at different willingness-to-pay thresholds for QALY gained.

| 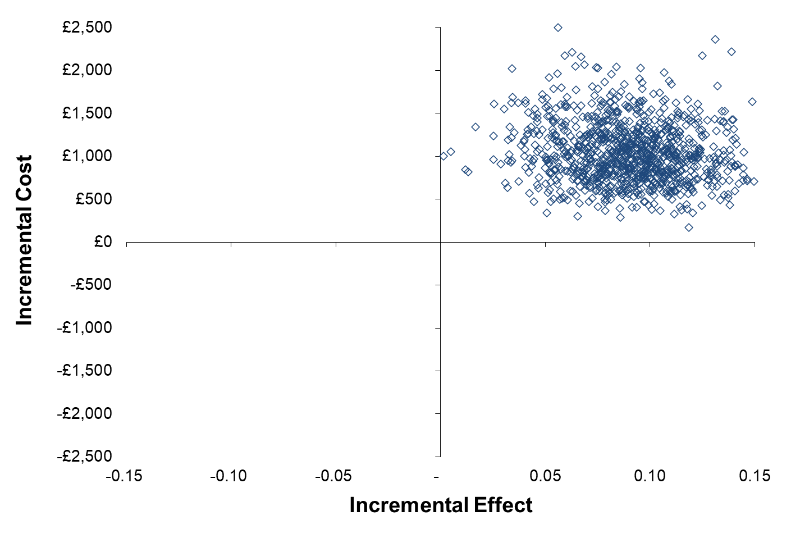  a | 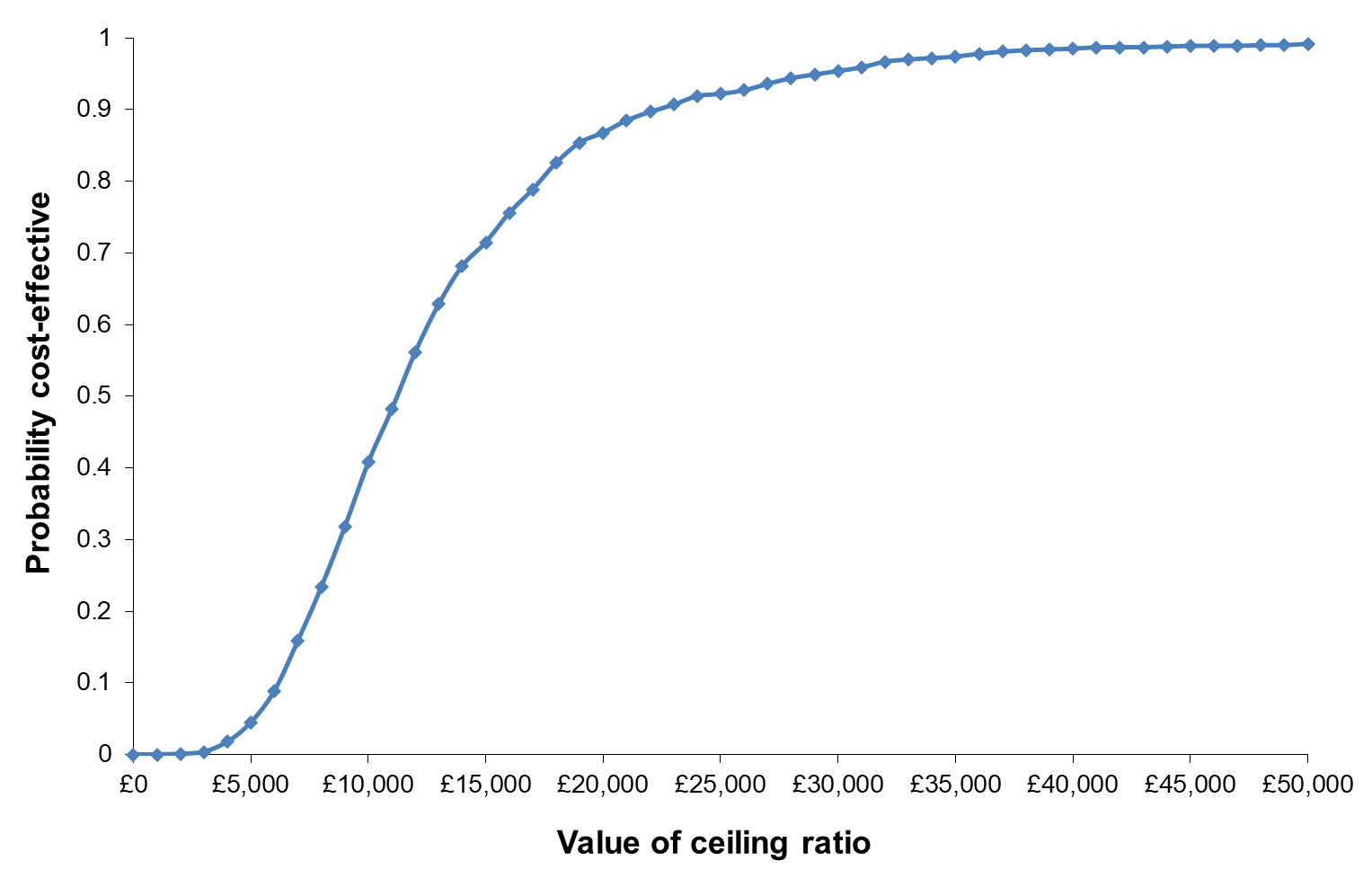 |
| --- | --- |
| 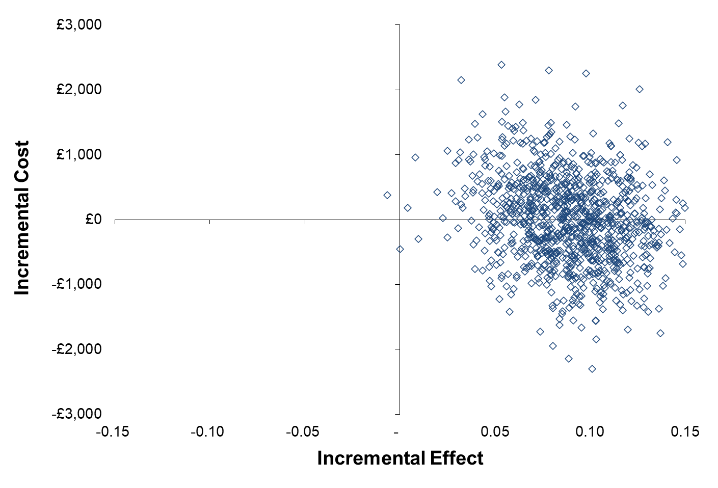  b | 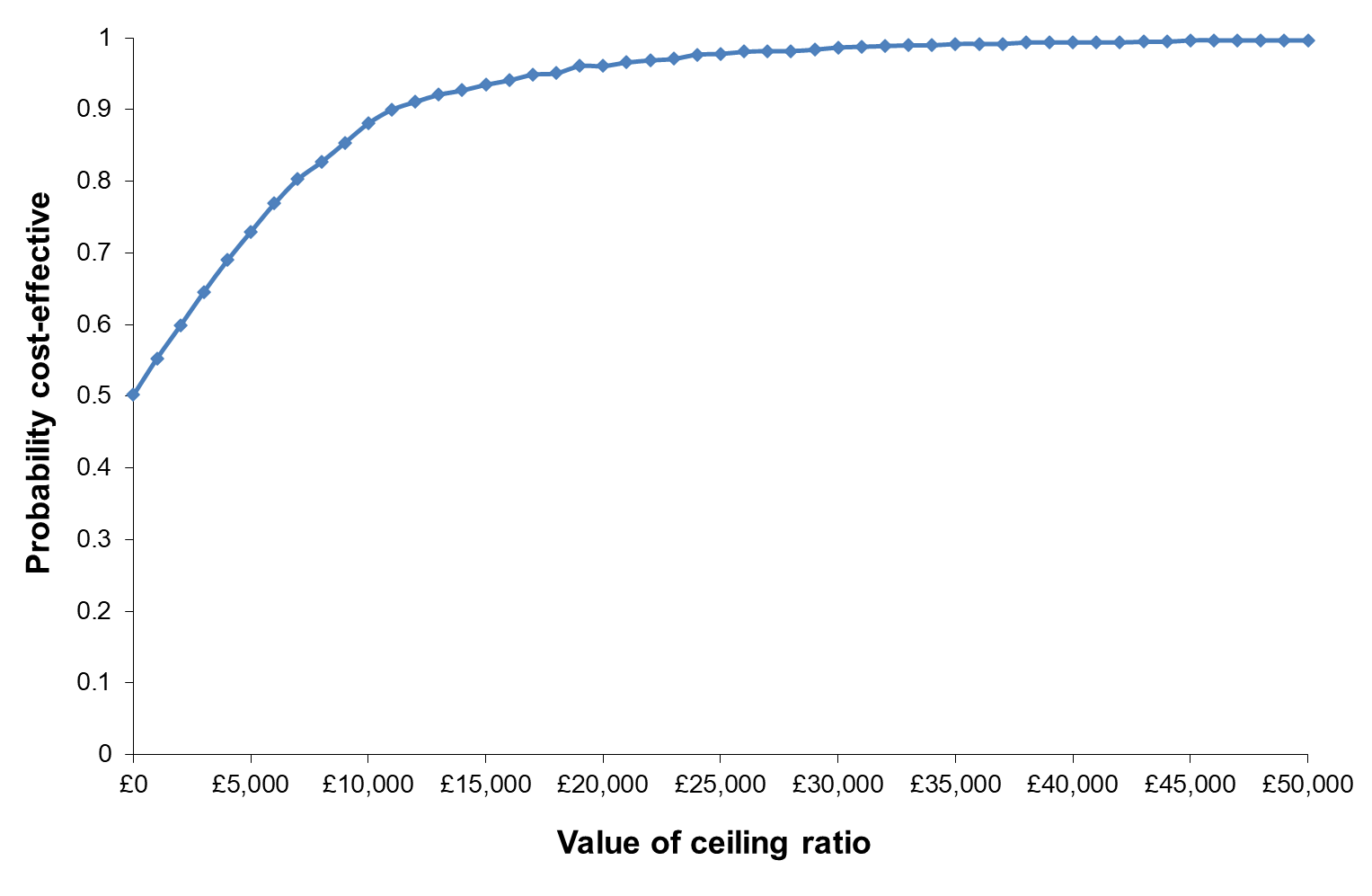 |

Appendix Figure 21. Sensitivity analysis: Cost-effectiveness for QALY over 48 weeks (current pramipexole cost)

Note: Left: Cost-effectiveness plane with bootstrapped ICERs for pramipexole against placebo treatment presenting results for the alternative pramipexole cost analysis for quality-adjusted life (QALY) gained over 48 weeks from the a) NHS+PSS perspective, and b) societal perspective;

Right: Cost-Effectiveness Acceptability Curve (CEAC) showing the probability of pramipexole being cost-effective in comparison to placebo treatment at different willingness-to-pay thresholds for QALY gained.
